# Supplementary material for: Abstracts from Rambam Research Day, December 11, 2014
Source: Rambam Maimonides Med J. 2015 Jan 29;6(1):e0009. doi: 10.5041/RMMJ.10184 (PMC4327325; doi:10.5041/RMMJ.10184)
Supplement: Related texts

# Sub article: About Rambam Research Day

**DOI:** 10.5041/RMMJ.10184

**Published:** 2015-01-29

We are proud to introduce you to the Eleventh Rambam Research Day, now established as a key annual event at Rambam Health Care Campus (Rambam), reflecting the diverse research activities here.

Integral to Rambam’s research activities are two competitive research funding programs that Rambam administers: The “Ofakim” program, which provides start-up and bridge research funding for young staff physicians with strong research credentials and plans, and the “Atidim” program, which is directed towards research training support for our residents, or as we call them in Israel “Mitmachim.” The Atidim program continues to receive wide recognition, and serves as an especially important example of Rambam’s dedication to medical trainees and the development of physician scientists.

We would like to acknowledge the excellent collaboration and the fruitful research ties between Rambam Health Care Campus and the Technion-Israel Institute of Technology, in particular The Ruth & Bruce Rappaport Faculty of Medicine. We would also like to thank Professor Chaim (Howard) Cedar from the Hebrew University of Jerusalem as the Chair of our Scientific Advisory Board.

We are particularly proud of the scientific abstracts that were presented at the meeting, and which compose this Supplement to *Rambam Maimonides Medical Journal*. The abstracts cover all areas of medicine. They reflect the rich spectrum of clinical, applied, and basic research endeavors that characterize our medical center’s activities. Indeed, our primary mission of bringing health care to the community is achieved as we combine these research discoveries together with excellence in teaching.

We would also like to recognize Rambam’s Research Division, including the Institutional Review Board (IRB), and the Office for Promotion of Research; together they are responsible for the coordination and oversight of all research activities at Rambam, including clinical research, applied research, competitive grants management, patent protection, institutional review board ethical approvals, and bringing forward innovative ideas to clinical practice. We value collaboration with industry, which serves as an integral part of health care progress; over the years, several Rambam-originated research projects have led to remarkable success in Israel, with practical application to health care.

We are particularly pleased to see this second Supplement to *Rambam Maimonides Medical Journal*, which has now become a tradition by *Rambam Maimonides Medical Journal*, for the awareness and promotion of research activities in Israel, particularly at Rambam. Supporting Rambam Research Day via *Rambam Maimonides Medical Journal* is another way we show commitment to our patients, via the furthering of health care facilitated by progress in science, innovation, and technology.

**Rafi Beyar, M.D., D.Sc.** ***Director, Rambam Health Care Campus*** ***and Associate Editor, Rambam Maimonides Med J******Karl Skorecki, M.D.*** ***Director, Medical & Research Development,*** ***Rambam Health Care Campus and Associate Editor, Rambam Maimonides Med J*****Michael Aviram, D.Sc.** *Director, Clinical Research Institute at Rambam,* *Rambam Health Care Campus and* *Deputy Editor, Rambam Maimonides Med J***Ehud Klein, M.D.** *Chair, Division of Psychaitry* *and Director, Research Division,* *Rambam Health Care Campus*

# Sub article: Rambam Research Day Organizing Committee

**DOI:** 10.5041/RMMJ.10184

**Published:** 2015-01-29

**Professor Ehud Klein, Chairman**

Director Division of Psychiatry, Head Research Division

**Professor Shraga Blazer**

Director, Department of Neonatology, and Editor-in-Chief, Rambam Maimonides Medical Journal

**Dr. Gil Bolotin**

Director, Department of Cardiac Surgery

**Professor Benjamin Brenner**

Director, Department of Hematology and Bone Marrow Transplantation

**Dr. Yael Eilon**

Nursing Research Coordinator

**Professor Ora Israel**

Director, Department of Nuclear Medicine

**Professor Yehuda Chowers**

Director, Gastroenterology Institute

**Professor Elon Isenberg**

Director, Pain Research Unit of the Pain Medicine Institute

**Dr. Orit Person**

Oncology Institute

# Sub article: Program for the Sixth Rambam Research Day

**DOI:** 10.5041/RMMJ.10184

**Published:** 2015-01-29

**Thursday, December 11, 2014**

Rambam Health Care Campus, Haifa, Israel

07.30 **Meet & Greet**

**Visit Posters Area**

08.00 **Opening Session: Greetings**

**Professor Rafael Beyar**

General Director, Rambam Health Care Campus

**Professor Eliezer Shalev**

Dean, The Ruth & Bruce Rappaport Faculty of Medicine, Technion-Israel Institute of Technology (IIT), Haifa, Israel

**Professor Karl Skorecki**

Director, Rappaport Research Institute, Technion-Israel Institute of Technology, Director of Medical and Research Development Rambam Health Care Campus

**Professor Michael Aviram**

Director, The Clinical Research Institute at Rambam (CRIR), at Rambam Health Care Campus

**Professor Ehud Klein**

Chairman, Research Division, Rambam Health Care Campus

**Honorary Lecturer**

08.30 **Professor Hermona Soreq**

Professor of Molecular Neuroscience The Edmond and Lily Safra Center for Brain Sciences Department of Biological Chemistry and The Alexander Silberman Institute of Life Sciences, The Hebrew University of Jerusalem, Jerusalem, Israel.

**From Brain to Body and Back: New Insights and Challenges in the Understanding of Cholinergic Signaling in Health and Disease**

**Panel Discussion**

09.15 **The Contribution and Role of Clinicians in Research**

**Moderator: Professor Karl Skorecki**

***Panel Participants***

Professor Rafael Beyar, Director, Rambam Health Care Campus

Professor Naim Ziv, Vice Dean for Research, The Ruth & Bruce Rappaport Faculty of Medicine, Technion-Israel Institute of Technology

Professor Gadi Shooster, Executive VP for Academic Affairs, Technion-Israel Institute of Technology

Professor Hermona Soreq, The Hebrew University

Dr. Ruth Perets, Division of Oncology, Rambam Health Care Campus

**Plenary Lectures**

09.45 **Professor Naim Shehedeh**

Director, Department Pediatrics A, Head, Pediatric Diabetes and Obesity Clinic, Rambam Health Care Campus

**New Infant Formula Enriched with Insulin: The Closest to Human Milk**

10.05 **Dr. Anat Aharon**

Thrombosis and Hemostasis Unit, Department of Hematology and Bone Marrow Transplantation, Rambam Health Care Campus, and The Ruth & Bruce Rappaport Faculty of Medicine, Technion-Israel Institute of Technology, Haifa, Israel

**Microvesicles in Health and Disease**

**Break**

**Honorary Lecturer**

11.00 **Professor Uri Sivan**

Bertoldo Badler Chair, Department of Physics, Technion-Israel Institute of Technology, Haifa, Israel

**The Interface between Nanotechnology and Medicine**

11.45 **Poster Presentations with Authors**

**Dr. Yoav Binenbaum**

Laboratory of Applied Cancer Science, Clinical Research Institute at Rambam (CRIR), Rambam Health Care Campus

**Exosomes Secreted by Macrophages Facilitate Chemotherapy Resistance in Pancreatic Ductal Adenocarcinoma**

**Dr. Inna Zoran**

Department of Internal Medicine C, and Hematology Institute, Thrombosis and Haemostasis Unit, Rambam Health Care Campus

**Microparticles Characterization in Patients with Acute Leukemia at Diagnosis and After Induction Remission Treatment**

**Dr. Tali Siman Tov**

Cognitive Neurology Institute, Rambam Health Care Campus

**Early Connectivity Decline in High-order Cognitive Networks**

**Awards for Rambam Research Day Oral Presentations**

**Pleanary Lecutres**

12.15 **Dr. Maty Tzukerman**

XXX, Rambam Health Care Campus

**The Role of Mesenchymal Stem Cells in Modulating the Behavior of Cancer Cells**

12.35 **Dr. Hannah Admi**

Director of Nursing, Rambam Health Care Campus

**Transition from Hospital to Community Care: The Experience of Cancer Patients**

14.15 **Closing Session**

Concluding Remarks

Awards for Rambam Research Day Poster Presentations

Awards for Rambam Atidim Graduates

Awards for Rambam Ofakim Graduates

Announcement of Rambam Atidim 2014 Recipients Rambam Ofakim 2014 Recipients

Announcement of Rambam Innovation Fund (Nitzoz) 2014 Awardees

# Sub article: From Brain to Body and Back: Checks and Balances of Cholinergic Signaling in Health and Disease

**DOI:** 10.5041/RMMJ.10184

**Published:** 2015-01-29

**Authors:** Hermona Soreq

**Affiliations:** The Edmond and Lily Safra Center for Brain Sciences and The Department of Biological Chemistry; The Hebrew University of Jerusalem; Safra Campus-Givat Ram; Jerusalem, Israel

## Abstract

One hundred years after the discovery of acetylcholine (ACh) by Otto Lowei, ACh receptors, transporters and synthesizing and degrading enzymes became well-recognized contributors to cognition, neuromuscular, metabolic and immune processes. However, newer technologies identified unexpected molecular controllers over ACh signaling, including the SLEEPLESS, Isl1 and Lynx1 genes. These regulators are responsible, among other effects to the fine-tuned identity, functioning modes, dynamics and inter-cellular interactions of cholinergic cell types in and out of the brain, changing our understanding of ACh’s roles in human health and wellbeing. Furthermore, Genome-Wide Association Studies identify new disease-associated mutations and single nucleotide polymorphisms in coding and non-coding sequences within these genes. These discoveries add autism, amyotrophic lateral sclerosis, acute cardiac events, narcolepsy and obesity to the established acquired and inherited neuromuscular, stress-induced, dementia and epilepsy disorders that were traditionally associated with impaired ACh functioning. At the molecular level, cholinergic signaling involves both up- and down-regulation events of transcription, epigenetic modulations, alternative splicing and microRNA suppression that together coordinate the multi-targeted ACh signaling in brain and body functions and are also responsible to the reactions of patients to anti-cholinesterase therapeutics of Alzheimer’s disease as well as to global exposure to agricultural pesticides and to individual tendencies for nicotine addiction, calling for new basic and translational research venues for regulating ACh signaling. Integrating these molecular ACh regulators into every discussion of cholinergic issues, should incorporate data obtained by clinicians and molecular geneticists, neuroscientists and structural biochemists over the past decades into a refreshed look at the intricate checks and balances over cholinergic signaling. Our understanding of the cholinergic regulators is incomplete, but time is ripe to summarize the recent reports on checks and balances of cholinergic signaling and their implications in health and disease.

# Sub article: The Role of Microvesicles in Health and Disease

**DOI:** 10.5041/RMMJ.10184

**Published:** 2015-01-29

**Authors:** Anat Aharon

**Affiliations:** Department of Hematology and Bone Marrow Transplantation, Rambam Health Care Campus, Haifa, Israel

## Abstract

There are millions of microvesicles (MVs) in the circulation of healthy persons, and their level may increase in a variety of pathologies. Microvesicles may be divided into sub-groups, i.e., exosomes, microparticles, and apoptotic bodies that are shed from both normal and malignant cells upon cell activation or apoptosis. Microvesicles promote clot formation, mediate pro-inflammatory processes, facilitate cell-to-cell interactions, transfer proteins and miRNA to cells, and induce cell signaling.

Over the last 10 years, we have studied the role of MVs in a variety of diseases including gestational vascular complications, diabetic vascular complications, cancer, i.e., solid tumors (breast, colon) and hematological malignancies (AML, myeloma, ALL) and in thalassemia. We have characterized MV structure, expression of membrane antigen, content of proteins and DNA/RNA, and evaluated their effects on cell cultures, including MV cell interaction, apoptosis/survival, migration, invasion, angiogenesis via signal transduction. The data acquired in these studies suggested that MVs may be used for wound healing and they may also serve as biomarkers for prothrombotic state and as a source for genetic diagnosis.

# Sub article: The Role of Mesenchymal Stem Cells in Modulating the Behavior of Cancer Cells

**DOI:** 10.5041/RMMJ.10184

**Published:** 2015-01-29

**Authors:** Liron Berger, Yeela Shamai, Karl Skorecki, Maty Tzukerman

**Affiliations:** 1 The Ruth & Bruce Rappaport Faculty of Medicine and Research Institute, Technion-Israel Institute of Technology, Haifa, Israel; 2 Molecular Medicine Laboratory, Rambam Health Care Campus, Haifa, Israel

## Abstract

**Introduction:**

Solid tumor associated nonneoplastic stromal cells also include mesenchymal stem cells (MSC) which are recruited by epithelial cancer cells from either the bone marrow or from adjacent tissue and become an integral cellular component of the tumor microenvironment where they modulate tumor progression. Nevertheless, there is insufficient information about MSC derived from human malignant tumors and the modulation of these cells by cancer cells. MSC can differentiate into multiple lineages, including adipocytes, chondrocytes and osteoblasts and they express specific cell surface markers such as CD29, CD73, CD90 and CD105. MSC tropism into tumor sites is mediated by extrinsic signals released into the tumor microenvironment and in turn promotes further cell recruitment through secretion of chemokines, cytokines and growth factors.

**Materials and Methods:**

To elucidate the mechanism whereby non-neoplastic stromal cells elicit their functional impact on tumor progression and modulate the behavior of the epithelial cancer cells, we have harvested cells from various anonymous patient tumor samples. In addition to the epithelial cancer cells, the harvested tumor-derived cells included stromal cells, identified as non-neoplastic tumor-derived MSC by their multi-potential capacity to differentiate *in vitro* into adipocytes, osteocytes and chondrocytes and by their surface expression profile of MSC specific markers signature. These procedures yielded also epithelial cancer cells and their counterpart MSC from gastric carcinoma (GSC1) and lung carcinoma (LC2).

**Results:**

While the LC2 cancer cell growth is independent of their LC-MSC, the GSC1 cancer cell growth is critically dependent on the presence of their counterpart GSC-MSC or their conditioned medium (CM). The fact that none of the various other TD-MSC were able to restore the specific effect of GSC-MSC on GSC1 cancer cell growth suggests specificity of TD-MSC, which is specifically recruited and ‘educated’/reprogrammed by the cancer cells to support tumor growth. Using cytokine array analysis, we were able to demonstrate that GSC1 cell growth is mediated through HGF/c-MET signaling pathway which is activated exclusively by HGF secreted from GSC-MSC. An innovative approach demonstrates GSC1-mediated specific tropism of ‘naïve’ MSC from the adjacent tissue a tumor specific manner to support tumor progression.

**Conclusions:**

The results suggest that specific tumor tropic ‘naïve’ MSC are reprogrammed in a tumor-specific manner to support gastric tumor progression. Understanding the mechanisms involved in the interactions of the tumor cancer cells and TD-MSC will constitute the basis for developing multimodal anti-cancer therapeutic strategies that will also take into account the specific tumor tropism properties of MSC and their reprogramming.

# Sub article: Transition from Hospital to Community Care: The Experience of Cancer Patients

**DOI:** 10.5041/RMMJ.10184

**Published:** 2015-01-29

**Authors:** Hanna Admi, Ella Muller, Lea Ungar, Nurit Naveh, Michael Kaffman, Nosaiba Rayan, Shmuel Reis, Efrat Shadmi

**Affiliations:** 1 Nursing Directorate, Rambam Health Care Campus, Haifa, Israel;; 2 Department of Family Medicine, Clalit Health Services, Haifa and the Western Galilee Region, Haifa, Israel;; 3 School of Public Health, Faculty of Social Welfare and Health Sciences, Haifa University, Haifa, Israel; 4 The Cheryl Spencer Department of Nursing, Faculty of Social Welfare and Health Sciences, Haifa University, Mount Carmel, Israel

## Abstract

**Background:**

This study examines the care transition experience of cancer patients and assesses barriers to effective transitions.

**Materials and Methods:**

Participants were adult Hebrew, Arabic, or Russian speaking oncology patients at Rambam’s Oncology Center. The study used qualitative and quantitative methods. Qualitative information was obtained from 33 healthcare providers (physicians, nurses, social workers, and administrative managers) from the hospital and community settings and 25 hospitalized patients (with their families)—all of which participated in focus groups and/or in-depth interviews. Quantitative information was obtained from 422 patients via validated questionnaire that assessed patients’ perceptions of the quality of their primary care using the Primary Care Assessment Survey (PCAS), patients’ care transition experience using the Care Transition Measure (CTM), as well as structured observations of the discharge explanations and language used during the hospital discharge process.

**Results:**

Qualitative analysis showed that patients face a complex and fragmented system with multiple providers and services. Difficulties in navigating the healthcare system are exacerbated for minorities with language and cultural barriers. Mechanisms to overcome barriers included undertaking of informal routes such as personal relationships, coordinating roles by nurse coordinators, and of the patients’ general practitioners (GPs). The involvement of GPs was found to be the most significant variable affecting the quality of the transition process as rated on the CTM (p<0.001).

**Conclusions:**

Our findings point to the important interpersonal role of the oncology nurses in coordinating and facilitating the care transition process. Interventions targeted towards supporting the care transition process should emphasize ongoing counseling throughout the course of a patient’s care during and after hospitalization.

# Sub article: Poster #035: The Molecular and Cellular Changes Occurring During Craniomaxillofacial Bone Distraction

**DOI:** 10.5041/RMMJ.10184

**Published:** 2015-01-29

**Authors:** Adi Rachmiel, Yoav Leiser

**Affiliations:** 1 Departments of Oral and Maxillofacial Surgery, Rambam Health Care Campus, Haifa, Israel; 2 The Ruth & Bruce Rappaport Faculty of Medicine, Technion-Israel Institute of Technology, Haifa, Israel

## Abstract

**Introduction:**

Gradual bone lengthening using distraction osteogenesis principles is the gold standard for the treatment of hypoplastic facial bones. However, the long treatment time is a major disadvantage of the lengthening procedures.

**Aim:**

the aim of the present study is to review the current literature and summarize the cellular and molecular events occurring during membranous craniofacial distraction osteogenesis.

**Results and Discussion:**

Mechanical stimulation by distraction induces biological responses of skeletal regeneration that is accomplished by a cascade of biologic processes that may include differentiation of pluripotential tissue, angiogenesis, osteogenesis, mineralization, and remodeling. There are complex interactions between bone-forming osteoblasts and other cells present within the bone microenvironment, particularly vascular endothelial cells that may be pivotal members of a complex interactive communication network in bone. Studies have implicated number of cytokines that are intimately involved in the regulation of bone synthesis and turnover. The gene regulation of numerous cytokines (transforming growth factor-b, bone morphogenetic proteins, insulin-like growth factor-1, fibroblast growth factor-2) and extracellular matrix proteins (osteonectin, osteopontin) during distraction osteogenesis has been best characterized and are discussed. Understanding the bio-molecular mechanisms that mediate membranous distraction osteogenesis may guide the development of targeted strategies designed to improve distraction osteogenesis and accelerate bone regeneration that may lead to shorten the treatment duration.

# Sub article: Poster #036: Mouse Model for the Study of Hepranase Role in Oral Cancer Formation

**DOI:** 10.5041/RMMJ.10184

**Published:** 2015-01-29

**Authors:** Yoav Leiser, Dekel Shilo, Imad Abu El Naaj

**Affiliations:** 1 Department of Oral and Maxillofacial Surgery, Rambam Health Care Campus, Haifa, Israel;; 2 The Ruth & Bruce Rappaport Faculty of Medicine, Technion–IIT, Haifa, Israel; 3 The Oral Cancer Research Laboratory, Oral and Maxillofacial Department, Rambam Health Care Campus, Haifa, Israel

## Abstract

**Introduction:**

Numerous studies have shown that metastases formation depends on the ability of tumor cells to invade basement membranes and tissue barriers in a process involving enzymes capable of degrading extracellular matrix (ECM) components. One of these enzymes is heparanase, an endoglycosidase that degrades heparan sulfate.

**Research Hypothesis:**

In the past we have shown that the nuclear localization of heparanase is found in all oral verrucous carcinomas, i.e., very well differentiated non-metastesizing tumors, as opposed to only 28% nuclear localization detected in oral squamous cell carcinomas. Heparanase expression level also significantly correlated with the degree of tumor differentiation. Moreover, while cytoplasmic localization of heparanase was associated with high grade carcinomas, nuclear localization of the enzyme was found primarily in low grade, well differentiated tumors. The aim of the present study was to use an in vivo model to study the role of heparanase in cancer formation and progression.

**Materials and Methods:**

4NQO is a water soluble carcinogen that induces tumors, predominantly in the oral cavity. It results in all the stages of oral carcinogenesis and evidence suggests that similar histological as well as molecular changes are observed. We used this carcinogen to establish a mouse model of oral tongue SCC (squamous cell carcinoma). Heparanase expression and activity was evaluated using immunohistochemical analysis and real time PCR (for protein and mRNA expression levels). Heparanase enzymatic activity was evaluated for both cytosolic and nuclear fractions of total proteins using a radio-labeled ECM (extracellular matrix) and a scintillation counter for the degradation products.

**Results:**

Heparanase was over expressed in tongue cancer from the hyperkeratosis stage through to invasive SCC. Heparanase enzymatic activity was significantly higher in the nucleus of normal tongue tissues as opposed to developing cancer, which exhibited a higher cytoplasmic activity of the enzyme.

**Conclusion:**

Heparanase nuclear expression and enzymatic activity is higher in normal tongue tissue. When carcinogenesis is initiated and progresses, heparanase translocates from the nucleus to the cytoplasm. The overall expression of heparanase increases as the cancer progress from the pre-malignant stage to invasive SCC. Hence, the expression level and cellular localization of heparanase could serve as reliable predictive indicators of oral carcinoma development, metastatic potential, and patient prognosis.

# Sub article: Poster #072: Cytoplasmic Function of HDAC4 Controls Ossification

**DOI:** 10.5041/RMMJ.10184

**Published:** 2015-01-29

**Authors:** Omer Shkedi, Lilac Caspi, Izhak Kehat

**Affiliations:** 1 The Ruth & Bruce Rappaport Faculty of Medicine, Technion-Israel Institute of Technology, Haifa, Israel; 2 Department of Cardiology, Rambam Health Care Campus, Haifa, Israel

## Abstract

**Introduction:**

Aortic valve stenosis caused by valvular calcification, a form of extra-skeletal ossification, is the most prevalent indication for valves replacement and is associated with high morbidity. Recent studies have shown that extra-skeletal ossification is a highly regulated process that involves the differentiation of mature vascular and stromal cells into osteochondrogenic cells and recapitulates embryonic endochondral ossification. Histone deacetylases (HDAC’s) are enzymes that are known to play a major role in chondrocytes and osteoblasts differentiation and maturation yet the role of HDACs in extra-skeletal ossification is still unknown.

**Materials and Methods:**

MOVAS cells, originated from mouse vascular aortic smooth muscle, were transfected with pcDNA plasmids encoding different HDAC’s (1, 3, 4, 7). RNA was extracted from the cells and RT-qPCR was performed to detect an increase in several known ossification markers. In addition, MOVAS and HeLa cells were transfected with pGFP plasmid for over expression of HDAC4 and observed under florescent microscope to assess the subcellular localization of HDAC4 in both cell types.

**Results:**

MOVAS cells were previously induced to ossify in culture. Gene expression analysis showed that the expression levels of HDAC4 were up-regulated, while HDAC’s 1, 3 and 7 levels did not change significantly. Over expression of HDAC4 in an aortic smooth muscle cells model (MOVAS cells) showed a significant increase in ossification markers (especially the transcription factor Osterix) at the mRNA level. HDAC4 is known to shuttle between the nucleus and cytoplasm and is presumed to function in the nucleus is deacetylating histone tails. Assessment of subcellular localization of HDAC4 in control HeLa cells demonstrated presence of the protein both in the cytoplasm and nuclear whereas in MOVAS cells HDAC4 was located almost exclusively in the cytoplasm.

**Conclusions:**

We showed that in aortic smooth muscle cells HDAC4 is up-regulated during ossification and positively contributes to the ossification process. Despite the believed role of HDAC4 as histone deacetlylase in the nucleus, we show that in aortic smooth muscle cells HDAC4 is found almost exclusively in the cytoplasm, and presumably deactylates cytoplasmic proteins. HDAC4 may be a target for future therapies aiming to limit valve and vascular calcifications.

# Sub article: Poster #081: TLR9 and PD1 Signaling in Chronic Lymphocytic Leukemia Cells Cooperate in the Generation of a Regulatory and Inhibitory Phenotype

**DOI:** 10.5041/RMMJ.10184

**Published:** 2015-01-29

**Authors:** Shimrit Ringelstein-Harlev, Irit Avivi, Shoham Arad, Tami Katz

**Affiliations:** 1 Department of Hematology and Bone Marrow Transplantation, Rambam Health Care Campus, Haifa, Israel; 2 The Ruth & Bruce Rappaport Faculty of Medicine, TechnionIIT, Haifa, Israel

## Abstract

**Introduction:**

Chronic lymphocytic leukemia (CLL) patients demonstrate impaired effector T-cell activity and a general exhausted T-cell phenotype. The mechanisms by which CLL cells inflict these changes are a subject of active investigation. We have previously shown that TLR9-activated CLL cells can inhibit autologous CD4^+^ T-cell proliferation and induce regulatory T-cells. Moreover, this activation induces PD1 and PDL1 expression on CLL B-cells, along with IL10, CD25, CD38, and CD23, markers characterizing regulatory B-cells (Bregs). The current study aimed to determine whether PD1/PDL1 increased expression contributes to the cells’ inhibitory activity.

**Materials and Methods:**

B-cells were isolated from peripheral blood mononuclear cells of untreated CLL patients. Cells were stimulated with ODN (TLR9 agonist) and co-cultured with autologous CD4^+^ T cells. T-cell proliferation was evaluated using the CFSE method following stimulation with anti-CD3/CD28 antibodies and IL2. Involvement of the PD1/PDL1 axis was examined by incubating B-cells with antiPD1 neutralizing antibodies. PD1 blockage effects on ODN regulated gene expression were tested by Real-time PCR. Inhibition of TLR9 signaling was accomplished by adding an IRAK4 (critical downstream kinase) inhibitor to B-cell cultures.

**Results:**

ODN-activated CLL cells inhibit autologous T-cell proliferation in 82% of patients (p<0.001), however in the presence of neutralizing PD1 antibodies inhibition is observed in only 71% of patients, with loss of statistical significance (p=0.128) [N=7]. Blocking the PD1 signal on CLL cells reverses the TLR9 mediated gene expression attenuation of CD5 and TLR9 itself, [fold 2.35± 0.58, fold 1.65±0.28, respectively, N=7]. Among all proteins upregulated by ODN activation, only IL10 and PDL1 show reduced expression [fold 0.78±0.1, fold 0.72±0.2, respectively, N=7] following IRAK4 inhibition.

**Conclusion:**

CLL cells “acquire” Breg characteristics, elicited by TLR9 activation, most probably promoting immune regulation and cancer evasion. The involvement of B-cell-associated PD1 in the inhibition of T-cell proliferation and in transcription of TLR9 regulated genes implies that TLR9 regulated effects depend on proper PD1 signaling. The role of the PD1/PDL1 axis and its cooperation with TLR9-elicited signals is further emphasized by the attenuation of IL10 and PDL1 expression by IRAK4inhibition, demonstrating the importance of these specific factors in promoting regulatory activity. The current study underscores the proficiency of CLL cells to recruit separate pathways operating cooperatively to support immune modulation and promote survival.

# Sub article: Poster #082: *In Vitro* Antimicrobial Activity of Vancomycin-eluting Bioresorbable β-TCP-polylactic Acid Nanocomposite Material for Load-bearing Bone Repair

**DOI:** 10.5041/RMMJ.10184

**Published:** 2015-01-29

**Authors:** Ayelet Raz-Pasteur, Carina Makarov, Israela Berdicevsky, Irena Gotman

**Affiliations:** 1 Internal Medicine A Department and Infectious Diseases Unit, Rambam Health Care Campus, Haifa, Israel;; 2 Department of Materials Science and Engineering, Technion-Israel Institute of Technology, Haifa, Israel; 3 Department of Microbiology, The Ruth & Bruce Rappaport Faculty of Medicine, Technion-Israel Institute of Technology, Haifa, Israel

## Abstract

**Introduction:**

Infection is a common complication of orthopedic implant procedures. The average overall infection rate in the US for fracture fixation and joint prostheses is estimated at 5%, but may be as high as 30% for open fracture reductions. Local delivery of antibiotics to the implant site is considered advantageous over systemic treatments, since increased drug concentrations can be delivered to the infected tissues while avoiding systemic side-effects. Release of antimicrobial agents from bone-healing devices can dramatically reduce the risk of implant-associated infection. We report on the fabrication and antimicrobial activity of a multifunctional load-bearing bioresorbable material that provides mechanical support to healing bone tissue along with slow release of an antibiotic drug.

**Objective:**

This study evaluated the effect on antibiotic activity of high pressure consolidation of high-strength beta-tricalcium phosphate (β-TCP)-polylactic acid (PLA) nanocomposites, with incorporated vancomycin at room temperature and at 120°C.

**Materials and Methods:**

Dense beta-tricalcium phosphate (β-TCP)-40 vol.% polylactic acid (PLA) nanocomposite containing 1 wt.% vancomycin (VH) was high pressure consolidated at 2.5 GPa, at room temperature, or at 120°C.

**Results:**

Over the course of 5 weeks in TRIS solution, the β-TCP-PLA-VH nanocomposite released approximately 90% of its drug load. Specimens consolidated at 120°C had the highest initial mechanical properties, maintaining 85% of their compressive strength and 30% of their bending strength after 5 weeks of drug release. Study of *in vitro* growth inhibition showed significant antimicrobial efficacy of VH-impregnated β-TCP-PLA against Methicillin-resistant S aureus, when exposed to both high (2×105 CFU/mL) and very high (1×108 CFU/mL) bacterial concentrations. Total eradication of the microorganisms was achieved after one week

**Conclusion:**

The results indicate that the developed high strength antibiotic-eluting β-TCPPLA nanocomposite may be a promising material for orthopedic surgical devices.

# Sub article: Poster #084: Excess Bleeding after Open Heart Surgery when Using a Third Generation Starch-Based Volume Expander, Tetraspan, as Compared with Human Albumin Solution

**DOI:** 10.5041/RMMJ.10184

**Published:** 2015-01-29

**Authors:** Shani Liran, Duek Ori, Adler Zvika, Kadry Esam, Rony-Reuven Nir, Gil Bolotin

**Affiliations:** 1 Department of Cardiac Surgery, Rambam Health Care Campus, Haifa, Israel; 2 The Ruth & Bruce Rappaport Faculty of Medicine, Technion-Israel Institute of Technology, Haifa, Israel

## Abstract

**Introduction:**

The current non-inferiority, prospective, randomized study compared the efficacy and safety of the third generation, starch-based volume expander tetraspan (TS) to human albumin solution (ALB). Following the FDA’s declaration doubting the safety of starch solutions used as volume expanders in critically ill patients (2013), we discontinued recruiting patients and performed a data analysis. Postoperative bleeding during the first 48 hours was defined as the primary outcome measure.

**Methods:**

Patients admitted for open-heart surgery were prospectively randomized to perioperative volume replacement therapy with TS or ALB. Preoperative variables were demographics, aspirin and plavix use, baseline creatinine, and procedure type. Pre- and postoperative variables were coagulation profiles, thromboelastography (TEG) and creatinine. Intraoperatively, bypass time and minimal temperature were monitored. Intra- and postoperative volumes of used TS and ALB were recorded. Postoperative bleeding, RBC, and blood products use during ICU stay, acute kidney injury, hospitalization length, and 30-day in-hospital mortality were assessed.

**Results:**

Thirty patients fulfilled the study protocol (TS group: n=18; ALB group: n=12). Perioperative TS and ALB volumes were comparable (5095±288cc vs. 5174±332cc, respectively; P= 0.923). The TS group demonstrated excess postoperative bleeding compared to the ALB group (median [25th–75th percentiles]: 965 [560–1312.5] vs. 610 [488.7–937] cc, respectively; P=0.038). Both groups were comparable in terms of all other variables (Ps>0.1). No renal replacement therapy or mortality cases were recorded.

**Conclusion:**

Increased bleeding was detected in patients treated with TS as compared to ALB after open heart surgery, although used volumes were comparable. This justifies vigilance before resumption of starch-based solutions ALB volume expanders in patients undergoing open-heart surgery.

# Sub article: Poster #085: Novel Emboli Protection System During Cardiac Surgery: A Multi-Center, Randomized, Clinical Trial

**DOI:** 10.5041/RMMJ.10184

**Published:** 2015-01-29

**Authors:** Gil Bolotin, Christoph Hans Huber, Liran Shani, Friedrich Wilhelm Mohr, Thierry P. Carrel, Michael Andrew Borger, Volkmar Falk, David Taggart, Rony-Reuven Nir, Lars Englberger, Joerg Seeburger, Etem Caliskan, Christoph Thomas Starck

**Affiliations:** 1 Department of Cardiac Surgery, Rambam Health Care Campus, and Faculty of Medicine, Technion-Israel Institute of Technology, Haifa, Israel; 2 University Clinic for Cardiovascular Surgery Inselspital, Bern, Switzerland.; 3 Heart Center Leipzig, University of Leipzig, Leipzig, Germany.; 4 University Hospital Zurich, Zurich, Switzerland.; 5 University of Oxford, Oxford, United Kingdom

## Abstract

**Introduction:**

Stroke is a major cause of morbidity and mortality during open-heart surgery. Up to 60% of intraoperative cerebral events are emboli induced. This randomized, controlled, multicenter trial is the first human study evaluating the safety and efficacy of a novel aortic cannula producing simultaneous forward flow and backward suction for extracting solid and gaseous emboli from the ascending aorta and aortic arch upon their intraoperative release.

**Methods:**

Sixty-six patients (25 females; 68±10 years) undergoing elective aortic valve replacement surgery, with or without coronary artery bypass graft surgery, were randomized to the use of the CardioGard (CardioGard Medical, Or-Yehuda, Israel) Emboli Protection cannula (“treatment”) or a standard (“control”) aortic cannula. The primary endpoint was the volume of new brain lesions measured by diffusion-weighted magnetic resonance imaging (DW-MRI), performed preoperatively and postoperatively. Device safety was investigated by comparisons of complications rate, namely neurologic events, stroke, renal insufficiency and death.

**Results:**

Of 66 patients (34 in the treatment group), 51 completed the presurgery and post-surgery MRI (27 in the treatment group). The volume of new brain lesion for the treatment group was (mean±standard error of the mean) 44.00± 64.00 versus 126.56±28.74 mm3 in the control group (p=0.004). Of the treatment group, 41% demonstrated new postoperative lesions versus 66% in the control group (p=0.03). The complication rate was comparable in both groups.

**Conclusions:**

The CardioGard cannula is safe and efficient in use during open-heart surgery. Efficacy was demonstrated by the removal of a substantial amount of emboli, a significant reduction in the volume of new brain lesions, and the percentage of patients experiencing new brain lesions.

# Sub article: Poster #086: Does Lack of Daylight Exposure Lead to Postoperative Delirium?

**DOI:** 10.5041/RMMJ.10184

**Published:** 2015-01-29

**Authors:** Beckerman Ziv, Kisselman Elvira, Rony-Reuven Nir, Adler Zvika, Cohen Oved, Gil Bolotin

**Affiliations:** Department of Cardiac Surgery, Rambam Health Care Campus, and The Ruth & Bruce Rappaport Faculty of Medicine, Technion-Israel Institute of Technology, Haifa, Israel

## Abstract

**Introduction:**

Postoperative delirium constitutes a range of neuropsychological disturbances with incidence of up to 57% of cardiac surgery patients. Lack of natural daylight exposure in the intensive-care setting induces a circadian rhythm disturbance, which may result in delirium. We evaluated the effect of lack of daylight exposure on the prevalence, intensity, and duration of postoperative delirium.

**Methods:**

Altogether, 942 patients were enrolled and constituted two groups: (i) Group D (Dark) of 485 patients, operated from October 10, 2010, to October 30, 2011, while the Department of Cardiac Surgery resided in the hospital’s underground floor, with no daylight exposure; (ii) Group L (Light) of 457 patients, operated from November 1, 2011, to October 1, 2012, while the department resided on the hospital’s 6th floor, with normal daylight exposure. The prevalence, intensity, and duration of delirium were retrospectively assessed by means of (i) haloperidol prescribed [yes/no]; (ii) its amount [mg]; and (iii) administration length [days], respectively.

**Results:**

During both periods, 190 patients developed delirium: 88 (19.2%) were from group L and 102 (21%) from group D (P=NS). Among females, delirium in group D lasted longer (3.7±4.2 vs. 2.4±3.9 days; P=0.028). The average haloperidol dose was larger in group D both in males (19.1±19.1 vs. 13.6±3.5; P=0.021) and females (19.0±14.4 vs. 10.5±3.9, P=0.004). The average postoperative length of stay was longer in group D (15.4±11.9 vs. 13.6±6.9 days; P=0.003).

**Conclusion:**

The study demonstrates that among patients with lack of daylight exposure: (i) both females and males experienced more intense delirium; (ii) females also suffered of longer delirium; and (iii) length of stay was longer. These findings emphasize the importance of daylight exposure for maintaining circadian rhythm and thus reducing the risk of developing postoperative delirium among cardiac patients.

# Sub article: Poster #024: Patient and Operator Radiation Dose using a Pelvic Lead Shield during Trans Radial Angiography: Three-fold Decrease to Operator but Double Exposure to the Patient

**DOI:** 10.5041/RMMJ.10184

**Published:** 2015-01-29

**Authors:** Anees Musallam, Ina Volis, Sveta Dedaiev, Eitan Abergel, Amit Soni, Sergey Yalonetsky, Arthur Kerner, Ariel Roguin

**Affiliations:** The Department of Cardiology, Rambam Health Care Campus, Haifa, Israel; 2 The Ruth & Bruce Rappaport Faculty of Medicine, TechnionIIT, Haifa, Israel

## Abstract

**Background:**

Cardiac angiography, using the radial access compared to the femoral approach, is associated with reduced complication rate and improved patient comfort but has significantly increased radiation dose. Improvements in radiation protection are needed. We aimed to determine the efficacy of a 0.5-mm lead apron across the patient’s abdomen in addition to standard operator protection for the reduction of scatter radiation on operator and patient radiation exposure.

**Methods and Results:**

We randomly assigned 332 patients undergoing coronary angiography to a group with pelvic lead shielding and a group without. In each procedure 8 digital dosimeters were used to measure operator radiation dose (under the lead apron, outside the thyroid shield, and at the left side of the head), patient dose at the level of the umbilicus (above and beneath the lead apron), and two on the acrylic shielding and one on the image receptor to measure scattered radiation.

Both groups were similar in BMI, procedures performed, and number of sequences. Usage of lead shielding statistically significantly reduced the radiation dose of the operator at all three sites measured: under lead apron (all in μSv): 0.53±1.4 Vs. 0.17±0.6, on thyroid collar 5.9±7.7 vs. 2.9±3.4 and left side of head 3.3±3.4 vs. 2.1±2.2, p<0.001. However, the radiation to the patient doubled from 15.4±24.1 to 28.9±81.1, p=0.04.

**Conclusions:**

The use of a pelvic lead shield during radial angiography reduced operator radiation exposure at multiple measurement sites. However there was an increased exposure to the patient. This balance has to be further investigated and taken into account before the widespread use of this method.

# Sub article: Poster #031: Histone Deacetylases (HDACs) are Potent Inducers of Calcification

**DOI:** 10.5041/RMMJ.10184

**Published:** 2015-01-29

**Authors:** Alon Abend, Lilac Caspi, Izhak Kehat

**Affiliations:** 1 The Ruth & Bruce Rappaport Faculty of Medicine, Technion-Israel Institute of Technology, Haifa, Israel; 2 Department of Cardiology, Rambam Health Care Campus, Haifa, Israel

## Abstract

**Introduction:**

Vascular and valvular calcifications are a form of extra-skeletal ossification. They affect the majority of the population, yet their pathogenesis is still unknown. Recent studies have shown that this highly regulated process involves the differentiation of mature vascular cells into osteochondrogenic cells and recapitulates embryonic endochondral ossification.

Histone deacetylases (HDACs) are enzymes that are known to play a major role in chondrocyte and osteoblast differentiation and maturation. These HDACs can be inhibited by small molecules, which have already been approved and/or are in clinical trials as therapeutic cancer drugs or anti-epileptic agents. The role of HDACs in extra-skeletal ossification is still unknown.

**Methods:**

In order to assess the role of HDACs in vascular calcifications *in vitro*, we used two different differentiation systems:

1. MC3T3-E1 cell system (these cells have similar gene expression profile to heart valve cells).
2. MOVAS cell system (vascular aortic smooth muscle cells).

Under specific culture conditions these cells can be induced to differentiate and mineralize into osteoblasts. We analyzed the expression levels of different HDACs during bone differentiation using qRT-PCR.

**Results:**

Our analysis showed that the expression levels of HDAC 4 were up-regulated, while HDACs 1, 3 and 7 levels did not significantly change. Next, we induced differentiation into osteoblasts in the presence of several HDAC inhibitors (HDACI): TSA, SAHA and TMP195. These compounds are known to selectively inhibit class I or II mammalian HDACs. We observed that HDACI strongly inhibited bone differentiation. This was documented by the considerable decrease in alizarin red calcium staining and in alkaline phosphatase staining, and by the almost complete abolition of the bone marker genes Runx2, Osterix, Osteocalcin, BMP2 and Col1A1 expression, using qRT-PCR.

**Discussion:**

In conclusion HDACIs are potent inhibitors of bone differentiation in the MC3T3-E1 and MOVAS cell model system of valvular and vascular cells. Further experiments are being conduct *in vivo* to determine the effect of TSA and SAHA on valvular and vascular ossification.

# Sub article: Poster #032: Genome Organization in Cardiac Hypertrophy: A Window into Cardiac Adaptation and Disease Progression

**DOI:** 10.5041/RMMJ.10184

**Published:** 2015-01-29

**Authors:** Tal Golan Lagziel, Izhak Kehat

**Affiliations:** 1 Department of Physiology, The Ruth & Bruce Rappaport Faculty of Medicine, Technion-Israel Institute of Technology, Haifa, Israel; 2 The Clinical Research Institute at Rambam and The Department of Cardiology, Rambam Health Care Campus, Haifa, Israel

## Abstract

**Introduction:**

Hemodynamic overload induces hypertrophy in cardiomyocytes that ultimately leads to cardiac decompensation and heart failure. The activation of several genes, termed the ‘fetal genes’ plays a key role in the process. Transcriptional control by enhancers involves the establishment of physical connections with promoters by genome folding. We hypothesize that the expression of the fetal genes, is governed by distal enhancers and aim to identify them. We further hypothesize that a shift in chromatin folding during hypertrophy allows different enhancers to interact with induced fetal genes promoters.

**Materials and Methods:**

To explore the molecular mechanism of the activation of this coordinated gene program during cardiac hypertrophy we used primary cultures of both quiescence and hypertrophied neonatal rat ventricular myocytes. Hypertrophy is induced by incubating cardiomyocytes under serum-free conditions in the presence of phenylephrine. We used the multiplexed 4C sequencing to detect of long-range interactions of the fetal gene promoters. The main steps of 4C-seq include treating cells with formaldehyde to cross-link protein-DNA interactions, digesting chromatin with a primary restriction enzyme, and religation under dilute conditions. In our 4C-seq application, the known fragment designed to be part of the fetal gene promoter and the unknown fragment is suspected as enhancer. The last step is high-throughput sequencing to determine the frequency of ligation events between genomic regions of interest, and this provided information about the folding of the genome.

**Results:**

We performed multiplexed 4C-seq using the promoters of the ‘fetal genes’ nppb, acta1, myh6, myh7, and cacna1g. We identified multiple in-cis (on the same chromosome as the promoter) and in-trans (on a different chromosome than the promoter) interactions and characterized them. Most of the genome-genome interactions between the ‘fetal gene’ promoters and distal sites were present both during quiescence and during hypertrophy. However about 35%–40% of the interactions differed between the two states. These experiments show that cardiac hypertrophy is consistently associated with changes in both in-cis and in-trans associations.

**Conclusions:**

We identified multiple in-cis and in-trans genome-genome interactions between the ‘fetal gene’ promoters and distal sites and characterized them. We also show for the first time that cardiac hypertrophy is consistently associated with changes in both in-cis and in-trans associations.

# Sub article: Poster #033: Nonsense Mutations in the Sodium Channel and Risk for Severe Cardiac Events in Brugada Syndrome

**DOI:** 10.5041/RMMJ.10184

**Published:** 2015-01-29

**Authors:** Miry Blich, Danna Spears, Raffaella Bloise, Nicola Monteforte, Mirella Memmi, Monther Boulos, Carlo Napolitano, Silvia G. Priori

**Affiliations:** 1 Division of Pacing and Electrophysiology, Department of Cardiology, Rambam Health Care Campus, and The Ruth & Bruce Rappaport Faculty of Medicine, Technion-Israel Institute of Technology, Haifa, Israel;; 2 Division of Cardiology, Toronto General Hospital, Toronto, Ontario, Canada;; 3 Molecular Cardiology, IRCCS Salvatore Maugeri Foundation, Pavia, Italy; 4 Department of Cardiology, University of Pavia, Pavia, Italy

## Abstract

**Introduction:**

Brugada syndrome, an inherited primary electrical heart disease, disposes to sudden cardiac death. Arrhythmic risk stratification in patients with Brugada syndrome remains challenging and new tools are needed. The objective of our work was to investigate whether nonsense mutations in the SCN5A gene, which result in truncated, non-functional protein are associated with a virulent clinical course.

**Materials and Methods:**

The study population was drawn from the database of Fondazione Salvatore Maugeri, IRCCS, Pavia, Italy. Subjects were included if they had a clinical diagnosis of Brugada syndrome and carried a SCN5A mutation. Family members, silent SCN5A mutation carriers were also included.

**Results:**

The study included 360 patients (mean age 38±17 years, 231 males) with Brugada syndrome and family members who carried 141 different SCN5A mutations. During mean follow–up (7.1±3 years), 59 patients experienced cardiac events (16%), 25 had severe cardiac events (7%), 19 experienced cardiac arrest (5%), and 3 died (0.8%). In multivariate logistic regression analysis, nonsense mutation was a significant risk factor for severe events (95%CI 1.01–8.95, OR=3, p=0.04), cardiac arrest (95%CI 1.08–10.7, OR=3.4, p=0.03), and a risk factor for cardiac arrest as the first manifestation of the disease (95%CI 1.08–12.2, OR=3.6, p=0.03). The increased risk in patients with nonsense mutation was independent of type 1 spontaneous pattern. The Kaplan-Meier curve plotted for first event cardiac arrest free survival revealed a significant difference between patients with and without nonsense mutation (95% CI 0.96–8.6, HR=2.8, p=0.05).

**Conclusions:**

In Brugada syndrome, patients with nonsense mutations were associated with higher risk for severe cardiac events, cardiac arrest, and higher risk to have cardiac arrest as the first manifestation of the disease, as compared to the Brugada syndrome patients without nonsense mutations. Genetic data might be an additional tool for risk stratification in BrS.

# Sub article: Poster #045: Regulation of Cardiac Growth by RhoJ

**DOI:** 10.5041/RMMJ.10184

**Published:** 2015-01-29

**Authors:** Ravit Cohen-Segev, Izhak Kehat

**Affiliations:** 1 The Ruth & Bruce Rappaport Faculty of Medicine, Technion-Israel Institute of Technology, Haifa, Israel; 2 The Clinical Research Institute at Rambam and Department of Cardiology, Rambam Health Care Campus, Haifa, Israel

## Abstract

**Introduction:**

Cardiac hypertrophy is the heart’s maladaptive response to elevated stress on the heart. Initially, the hypertrophic process normalizes wall tension; however, prolonged hypertrophy is associated with arrhythmias and development of heart failure. Despite extensive research, the molecular mechanisms involved in cardiac hypertrophy remain uncertain. The three best characterized Rho GTPases in the realm of cardiac hypertrophy are RhoA, Rac1, and Cdc42. While RhoA and Rac1 exerted *in vivo* and *in vitro* prohypertrophic effects, Cdc42 reduced hypertrophic growth in both mice with a specific cardiac knockout of Cdc42 and in cultured myocytes. A Cdc42 homologue, RhoJ, is enriched in endothelial cells and in the heart. Here we investigate the potential role of RhoJ as an anti-hypertrophic agent in the heart.

**Materials and Methods:**

To determine the effects of RhoJ on cardiac myocyte hypertrophy we isolated neonatal rat ventricular myocytes and infected them with adenoviral vectors encoding for RhoJ wildtype, RhoJ dominant negative (T17N mutant), RhoJ constitutive active (Q79L mutant) or lacz (control) and stimulated to cardiac hypertrophy with serum free medium containing phenylephrine. Hypertrophy was determined by qPCR assessment of hypertrophy markers (ACTA1, NPPA, NPPB) and histology.

**Results:**

(Figure 1): Phenylephrine-induced myocytes infected with RhoJ wildtype or RhoJ constitutive active displayed reduced hypertrophy compared to control. Specifically, both RhoJ wild-type and RhoJ constitutive active reduced NppA and NppB levels by ∼50%. Both RhoJ wildtype and RhoJ constitutive active reduced Acta1 levels by 39% and 56%, respectively. In contrast, myocytes infected with RhoJ dominant negative showed no change in cardiac growth.

**Conclusions:**

Our results suggest that RhoJ is a cardiac hypertrophy suppressor and acts directly on cardiomyocytes.

# Sub article: Poster #055: The Influence of Renal Function on the Prognosis of Patients Hospitalized with Acute Decompensated Heart Failure

**DOI:** 10.5041/RMMJ.10184

**Published:** 2015-01-29

**Authors:** Nizar Andria, Ihab Abboud, Geula Klorin, Doron Aronson, Gidon Berger, Zaher S. Azzam

**Affiliations:** 1 Internal Medicine “B”; 2 Heart Institute, Rambam Health Care Campus, Haifa, Israel;; 3 The Ruth & Bruce Rappaport Faculty of Medicine; 4 The Research Institute in Medical Sciences, Technion-Israel Institute of Technology, Haifa, Israel

## Abstract

**Introduction:**

Acute Decompensated Heart Failure (ADHF) is the leading cause of hospital admissions in patients age 65 and above. Identifying risk factors associated with increased morbidity and mortality may improve treatment, and clinical outcomes. Recently, B-Natriuretic Peptide (BNP), a protein secreted by the ventricular myocardium in response to an increased mechanical load and wall stretch, is being used as a diagnostic and prognostic marker in heart failure. The decrease in renal perfusion in patients with heart failure is due to the decrease in contraction and cardiac output. As a result, nephrons reduce the glomerular filtration and increase the re-absorption of salt and fluid, leading to a decrease in excretion of urea in the urine, and increased blood urea nitrogen (BUN).

**Methods and Results:**

This analytical, observational and retrospective study, collected demographic, laboratory, and echocardiography data from the medical records of 542 ADHF patients who had BNP blood tests, and were hospitalized at Rambam Health Care Campus, regardless of the reason for admission. Seventy-five percent of the patients were over 70 years old. The mortality rate was the highest in the fourth quartile of BUN.

**Conclusions:**

Blood urea nitrogen is an independent predictor of increased morbidity and mortality in patients with decompensated heart failure.

# Sub article: Poster #019: Direct Role of Expanded Human Endothelial Progenitor Cells in Vasculogenesis

**DOI:** 10.5041/RMMJ.10184

**Published:** 2015-01-29

**Authors:** Hadar Zigdon Giladi, Gal Michaeli-Geller, Eli E. Machtei

**Affiliations:** 1 Department of Periodontology Rambam Medical Center, Haifa, Israel; 2 The Ruth & Bruce Rappaport Faculty of Medicine at the Technion-Israel Institute of Technology, Haifa, Israel

## Abstract

**Introduction:**

Successful bone tissue engineering relies on early neovascularization in order to supply oxygen and nutrition to the graft. The aim of this study was to enhance neovascularization at bone regeneration sites, by local transplantation of human blood derived endothelial progenitor cells (hEPC).

**Materials and Methods:**

Human blood derived endothelial progenitor cells were isolated from adults, expanded and characterized. Expanded hEPC (5×105 cells) were mixed βTCP and transplanted in guided bone regeneration (GBR) model that was established by fixing a gold dome to nude rat calvarium (n=12). Domes filled with βTCP alone served as control (n=12). Rats were sacrificed after 3 or 5 months. Histomorphometry was used to analyze blood-vessel density (Bv/mm2) and immuohistochemistry was used to trace the transplanted cells in the newly regenerated bone.

**Results:**

Expanded hEPC presented a polygonal morphology, formed homogenous monolayer cells and replicated rapidly. Two days after seeding on Matrigel, cells spontaneously formed capillary-like structures. The FACS analysis demonstrated that >95% of the cells were positive for CD31 (endothelial marker), but negative to CD14, ruling out their monocytic origin. Local transplantation of hEPC significantly increased vascularization and stimulated bone formation. Three months post transplantation, blood vessel density was elevated by ∼700% in the hEPC transplanted group, as compared to the control group (1.08±0.29 vs. 7.52±1.2; Bv/mm2; p≤0.0001; control vs. hEPC, respectively). At 5 months, blood vessel density was still significantly higher in the hEPC transplanted group (1.06±0.19 vs. 9.52±0.69; Bv/mm2; p≤0.0001; control vs. hEPC, respectively). Transplanted hEPC were localized in the newly formed bone using immuno-staining with human-specific CD31 antibodies that do not react with rat CD31. Transplanted cells were found lining blood vessel walls in the newly regenerated tissue.

**Conclusion:**

Transplanted hEPC increased neovascularization at bone regeneration sites. The presence of hEPC in vessels walls implies on their direct role in vasculogenesis.

# Sub article: Poster #060: Simplified Busulfan TDM: An Opportunity to Refine Dose Personalization

**DOI:** 10.5041/RMMJ.10184

**Published:** 2015-01-29

**Authors:** Daniel Kurnik, Edna Efrati, Yedidia Bentur, Norberto Krivoy, Dorit Fink, Inna Scherb, Gil Ring, Zvi Teitelbaum

**Affiliations:** Clinical Toxicology and Pharmacology Section, Rambam Health Care Campus, Haifa, Israel

## Abstract

**Introduction:**

Busulfan (BU) is commonly used in conditioning regimens for hematopoietic stem cell transplantation. As a narrow-therapeutic-index drug with highly variable pharmacokinetics (PK), its therapeutic drug monitoring (TDM) is recommended. The area under the BU plasma concentration (C_pl_)-time curve (AUC) serves as a measure of exposure, and sub- and supra-therapeutic AUCs are associated with graft failure and toxicity, respectively. A relatively expensive and burdensome intense sampling strategy (ISS) of 9–10 blood samples is traditionally used (including by us). Limited sampling strategies (LSS) of 4–5 samples have been reported but have not been tested in Israeli patients. Our aim was to test the validity of LSS in this patient population.

**Methods:**

Our database containing PK BU data (analysis of ∼1800 samples collected by ISS, ∼200 intravenously-dosed patients) was used to calculate several PK parameters for each patient: AUC_ISS_ was calculated by the trapezoidal rule (TR) using C_pl_ measured separately in all 9 ISS samples. AUC_combo_ was estimated by analyzing one single sample composed of aliquots from these ISS samples (aliquot volumes defined by an algorithm previously developed in our laboratory). LSS parameters: AUC_5_, AUC_4_ and AUC_3_ were estimated by TR and multiple linear regressions (MLR) using C_pl_ of 5, 4 or 3 blood samples. These were used to estimate statistics such as percentage of individual parameters deviating from AUC_ISS_ by > 10% (PER_DEV_) and AUC_ISS_/parameter ^±^ SD.

**Results:**

The results are shown in Table 1.

**Conclusions:**

Deviation by >10% of AUC_5_ from AUC_ISS_ in only 5–6% of patients in this preliminary analysis suggests that a refined and reliable LSS (an ongoing project) for Israeli patients is feasible. Characterization of patients suitable for LSS will contribute to BU TDM personalization. Limited sampling strategies will reduce patient discomfort, clinical staff workload, and TDM costs. These will allow repeated TDM after dose adjustments, further improving personalized treatment. Improvement of the “combo” method may offer a rapid laboratory response for dose adjustments.

# Sub article: Poster #061: Comparison of Everolimus QMS Immunoassay on Architect CI4100 and LC-MS/MS: Lack of Agreement in Organ Transplanted Patients

**DOI:** 10.5041/RMMJ.10184

**Published:** 2015-01-29

**Authors:** Edna Efrati, Daniel Kurnik, Erica Hoffer, Inna Scherb, Marina Karasik, Gil Ring, Yedidia Bentur

**Affiliations:** 1 Toxicology, Pharmacology and Pharmacogenetics Laboratory; 2 Israel Poison Information Center; 3 Clinical Pharmacology Unit, Rambam Health Care Campus, Haifa, Israel; 4 The Ruth & Bruce Rappaport Faculty of Medicine, Technion-Israel Institute of Technology, Haifa, Israel

## Abstract

**Introduction:**

Liquid chromatography with mass spectrometry (LC-MS/MS) is the method of choice for the determination of everolimus whole blood concentrations, but is not routinely available. Therefore, immunoassays have been developed for clinical monitoring of everolimus. In previous studies, the QMS immunoassay had a positive bias compared to LC-MS/MS, but was judged acceptable, although clinical agreement (e.g. 95% limits of agreement) was not reported. The objective of this study was to assess whether the agreement between the QMS assay and the reference LC-MS/MS method was clinically acceptable for using them interchangeably in therapeutic everolimus monitoring.

**Materials and Methods:**

Whole blood samples from organ transplanted patients on everolimus therapy were analyzed by both QMS (on Architect ci4100 analyzer) and LC-MS/MS. Paired results were compared using paired Student’s t-test, Bland-Altman plots, and Deming regression analysis. The proportion of falsely supra- and sub-therapeutic results on the QMS assay compared to the reference LC-MS/MS were calculated.

**Results:**

Among 250 samples (169 patients), mean everolimus concentrations determined by LC-MS/MS and QMS assays were 4.8±2.1 and 6.3±2.1 ng/mL, respectively (p<0.001), with 95% lines of agreement between −2.1 to 5.2 ng/mL, a range corresponding to 152% of the mean concentration. When stratified by type of transplant, a similar positive bias was found in each subgroup (all p < 0.014). 69% of the samples yielding supra-therapeutic concentrations on the QMS assay were within the therapeutic range on the LC-MS/MS.

**Conclusion:**

The everolimus QMS immunoassay, using the Architect ci4100 analyzer, had a significant positive bias compared to LC-MS/MS, with a wide range between the limits of agreement. The lack of agreement may result in inadequate everolimus dose adjustments, suggesting that the QMS assay cannot be used interchangeably with the LC-MS/MS method for therapeutic everolimus monitoring in organ transplanted patients.

# Sub article: Poster #075: Tooth Survival and Probing Depth Changes in Periodontal Patients: Long-term Follow-up

**DOI:** 10.5041/RMMJ.10184

**Published:** 2015-01-29

**Authors:** Michael Saminsky, Michal Halperin-Sternfeld, Eli E. Mactei, Jacob Horwitz

**Affiliations:** Department of Periodontology, Rambam Health Care Campus, Haifa Israel.

## Abstract

**Introduction:**

We performed a retrospective assessment of the tooth survival rate and its association with patient and oral variables in patients who were followed for up to 18 years.

**Materials and Methods:**

A private periodontal office provided anonymous data for their patients. Patient data were divided into three groups: baseline (T_0_), reevaluation after cause-related therapy (T_Re_), and reevaluation at the last recorded visit 8 to 18 years later (T_F_). Inclusion criteria were receipt of periodontal therapy and supportive periodontal therapy (SPT). General health, plaque scores (PI), probing depth (PD), and bleeding on probing (BOP) at six points/tooth, extractions, and the number of SPT visits were also obtained at T_0_, T_Re_, and T_F_. For each patient, mean PI, PD, and BOP indices, and SPT/year were calculated at different time points. Teeth were classified as multi-rooted for molars and single-rooted for others. Descriptive statistics and Cox regression analysis were performed for assessing the putative factors affecting tooth survival.

**Results:**

The inclusion criteria were met by 50 patients. There were 1301 teeth between all patients (mean 26±4 teeth/patient). There were 20 extractions before T_Re_ and 129 after T_Re_. Of these, 96 were extracted for periodontal reasons. The annual extraction rate per patient was 0.16. The Proportional Hazards Model indicated that PD>7mm at T_Re_ (HR=17.7, 95%CI 8.6, 36.6), over 60 years of age (HR=3.3, 95%CI 1.5, 7.2), multi-rooted teeth (HR= 1.9, 95%CI 1.2, 3.1), and SPT<3 times per year (HR=1.8, 95%CI 1.1, 2.9), correlated with increased tooth loss during follow-up (P<0.05, Cox regression analysis). A continuous, statistically significant reduction was observed in mean PD among teeth that survived the follow-up period (4.3±1.8, 3.5±1.4, 3.2±1.3, at T_0_, T_Re_, and T_F_ respectively; p<0.001, repeated measures test).

**Conclusion:**

Active periodontal treatment followed by regular SPT results in relatively low tooth loss rates and a continuous reduction in probing depth. Probing depth after the initial phase of therapy, over 60 years old at admittance, multi-rooted tooth type, and infrequent SPT were found to be strong predictors for long-term tooth survival among periodontally maintained patients.

# Sub article: Poster #001: The Effect of Cholesterol on Development of Human Breast Cancer Cells in a Mouse Model

**DOI:** 10.5041/RMMJ.10184

**Published:** 2015-01-29

**Authors:** Eyal J. Scheinman, Sarit Ben-Shmuel, Ran Rostoker, Avishay Caspi, Zila Shen-Orr, Derek LeRoith

**Affiliations:** 1 The Ruth & Bruce Rappaport Faculty of Medicine, Technion–IIT, Haifa, Israel; 2 Diabetes and Metabolism Clinical Research Center of Excellence, Clinical Research Institute at Rambam (CRIR), Rambam Health Care Campus, Haifa, Israel

## Abstract

**Introduction:**

Dyslipidemia has been associated with an increased risk for developing cancer. To explore the role of dyslipidemia in breast cancer growth and metastasis, we used the apolipo-protein E (ApoE) knockout mice (ApoE −/−), which exhibit marked dyslipidemia, with elevated circulating cholesterol and triglyceride levels in the setting of normal glucose homeostasis and insulin sensitivity. We previously showed that Mvt-1 mouse mammary cancer cells developed larger tumors and more metastasis in ApoE −/− compared to WT mice. Here we wanted to extend our research with the human mammary cancer cell line MDA-MB-231.

**Materials and Methods:**

ApoE −/− mice were crossed with immunodeficient Rag1 −/− mice. The control Rag1 −/− and Rag/ApoE −/− mice were fed a high fat diet (HFD) for 10 weeks after which the human mammary cancer cell line MDA-MB-231 were injected to the fourth mammary fat pad. Mice were followed for their weight gain and tumor volume.

**Results:**

Rag/ApoE −/− mice gained more weight when fed a HFD compared with Rag/WT mice, ensuring these mice retained their ApoE −/− phenotype. Rag/ApoE −/− mice injected with these cells into the fourth mammary fat pad developed larger tumors compared to Rag/WT mice when fed a HFD.

**Conclusions:**

These results suggest that hypercholesterolemia enhances the growth of human breast cancer and may explain the association seen in patients and their responsiveness to statin therapy.

# Sub article: Poster #018: “An ounce of prevention is worth a pound of cure:” Cotreatment with GnRH-agonist Before and In Parallel to Gonadotoxic Chemotherapy Significantly Preserves Fertility and Increases Pregnancy Rate in Addition to Cyclic Ovarian Function

**DOI:** 10.5041/RMMJ.10184

**Published:** 2015-01-29

**Authors:** Hilly Zur, Ronit Leiba, Eldad J Dann, Tsilla Zuckerman, Naomi Schultz, Alexandra Balbir-Gurman, Zeev Blumenfeld

**Affiliations:** 1 Reproductive Endocrinology, Department of Obstetrics and Gynecology; 2 Quality Control; 3 Hematology; 4 Rheumatology, Rambam Health Care Campus, The Ruth & Bruce Rappaport Faculty of Medicine, Technion-Israel Institute of Technology, Haifa, Israel

## Abstract

**Introduction:**

Several recent meta-analyses including over 10 RCT concluded that GnRHa co-treatment along chemotherapy significantly decreased premature ovarian failure (POF) rate. However, cyclic ovarian function is not equivalent to fertility (pregnancies). Therefore, we evaluated the pregnancy rate (PR) after exposure to gonadotoxic chemotherapy+GnRHa vs controls.

**Methods:**

GnRH-a was administered to 281 patients in parallel with gonadotoxic chemotherapy, and compared to 173 patients of a comparative age, who were similarly treated, but without GnRH-a (control group). Patients who had not visited our clinic for more than a year were interviewed to verify the PR and outcome. The study was approved by the Institutional Review Board (Helsinki) Committee.

**Results:**

Less than 12% of the GnRHa group developed POF, vs 50% in controls (P<0.05). The remaining resumed cyclic ovarian function; 81 patients conceived 161 times and were delivered of 119 healthy neonates. In the control group 30 patients spontaneously conceived 49 times (P<0.05). In the GnRHa group, 88% resumed cyclic ovarian function (COF) vs only 50% of the controls; the rest suffered POF (P=0.003). Of the survivors in the GnRHa group 62% conceived, vs 42% of the controls (P=0.033). Spontaneous pregnancies occurred in 57.7% of the survivors in the GnRHa group (up to 6 pregnancies/patient), vs 34.9% of the controls (P=0.009). The age at chemotherapy for those who spontaneously conceived was 14–38 years in the GnRHa group, vs. 14–28 years in the control group, suggesting a possible prolongation of the “Fertile window” by 10 years. One patient in the GnRHa group spontaneously conceived three times and was delivered of three healthy neonates, despite two stem cell transplantations (SCT) 11 years apart. GnRH-a cotreatment significantly decreased POF rate, not only in conventional chemotherapy, but also in lymphoma patients undergoing aggressive conditioning before SCT.

**Conclusions:**

GnRHa co-treatment in parallel to chemotherapy is beneficial in minimizing POF rate and increasing pregnancy rate. Therefore, young women facing gonadotoxic chemotherapy should be offered the options of fertility preservation by GnRHa in addition to IVF and cryopreservation of embrya, ova, and ovarian tissue.

# Sub article: Poster #053: Global Adipose Tissue Gene Expression in Acromegaly

**DOI:** 10.5041/RMMJ.10184

**Published:** 2015-01-29

**Authors:** Irit Hochberg, Quynh T. Tran, Ariel R. Barkan, Alan R. Saltiel, William F. Chandler, Dave Bridges

**Affiliations:** 1 Endocrinology Institute, Diabetes and Metabolism, Rambam Health Care Campus, Haifa, Israel;; 2 Life Sciences Institute, University of Michigan, Ann Arbor, MI;; 3 Department of Preventive Medicine, University of Tennessee Health Science Center, Memphis, TN;; 4 Department of Internal Medicine, University of Michigan, Ann Arbor, MI, USA;; 5 Department of Neurosurgery, University of Tennessee Health Science Center, Memphis, TN, USA;; 6 Department of Physiology, University of Tennessee Health Science Center, Memphis, TN, USA; 7 Children’s Foundation Research Institute, Le Bonheur Children’s Hospital, Memphis, TN, USA.

## Abstract

**Introduction:**

Acromegaly, excess growth hormone (GH) production secondary to a pituitary adenoma, has important metabolic effects. The two most significant effects of GH on metabolism in adipose tissue are insulin resistance and lipolysis. The objective of this study was to determine the effect of chronic excess growth hormone in acromegaly on gene expression in adipose tissue in humans.

**Materials and Methods:**

To study the effect of chronic excess growth hormone on adipose tissue we performed RNA sequencing in adipose tissue biopsies from patients with acromegaly (n=9) or non-functioning pituitary adenomas (n=11). The patients underwent preoperative clinical and metabolic profiling including assessment of HOMA-IR. Explants of adipose tissue were assayed *ex vivo* for lipolysis and ceramide levels.

**Results:**

Patients with acromegaly had higher glucose, higher insulin levels and higher HOMAIR score. There was a trend for enhanced *ex vivo* adipose tissue lipolysis in acromegaly patients. We observed several expected transcriptional changes (IGF1, IGFBP3, SOCS2) as well as several novel transcriptional changes, some of which may be important for GH signal regulation and the effect of GH on growth and proliferation. Several transcripts could potentially be important in GH-induced metabolic changes. We have identified specific transcripts that could contribute to enhanced lipolysis. Higher expression of fatty acid desaturases could contribute to insulin resistance. Ceramides were not different between the two groups.

**Conclusions:**

We have identified the acromegaly gene expression signature in human adipose tissue. The significance of altered expression of specific transcripts will enhance our understanding of the metabolic and proliferative changes associated with acromegaly.

# Sub article: Poster #076: Safety of Zoledronic Acid in Post-menopausal Women with Osteoporosis: A Single Center Observational One-year Study

**DOI:** 10.5041/RMMJ.10184

**Published:** 2015-01-29

**Authors:** Elena. Segal, Marina Nodelman, Sophia Ish-Shalom

**Affiliations:** 1 The Endocrine Institute, Rambam Health Care Campus, Haifa, Israel; 2 The Ruth & Bruce Rappaport Faculty of Medicine, Technion-Israel Institute of Technology, Haifa, Israel

## Abstract

**Introduction:**

In clinical trials, treatment with Zoledronic acid (ZOL) was reported to induce acute phase reaction in about 15% of patients after the first infusion. This study was aimed at evaluating the incidence of adverse events (AE) when using ZOL in the regular clinical setting.

**Patients and Methods:**

The study included 215 consecutive female patients with an average age of 69.7±9.59, (45–100); 151 (70%) had been previously treated with Alendronate 65 pts (43%); Raloxifene 9 (5.96%); Risedronate −31 (20.5%); Teriparatide 23 (15.2%); Livial-1 (0.7%); calcium supplements133 (61.9%); Vitamin D 147 (68%) All patients received IV ZOL 5 mg; of these, 52 received a second infusion of ZOL. Patients were provided with a detailed form to complete and were asked to report adverse events over the ten days following ZOL infusion.

**Results:**

Adverse events were reported in 136 (63.3%) of the patients after the first ZOL dose, and in 32 (61.5%) after the second dose. The AEs experienced were: fever 69 (50.7%) and 10 (31.3%), p=0.024; bone/joint pain/headache 129 (94.8%) and 0, p<0.0001; weakness 109 (80.1%) 20 (62.5%), p=0.05; muscles pain 106 (77.9%) and 10 (31.3%), p=0.04; and uveitis 2 (1.47%) and 0, after the first and second doses of ZOL, respectively. The duration of AEs was 2–5 days, with no correlation to prior treatments. It is worth noting that previous factures were reported in 114 (53%) patients. Of these, there were 5 (2.3%) new fractures: 3 to the hip; 2 of the ramus pubis.

**Conclusion:**

The incidence of acute phase reaction in this study was higher than previously reported, with a significant decrease after the second dose of ZOL.

# Sub article: Poster #077: Bone Density and Vitamin D Status in Liver Transplant Patients 12 Years After the First Assessment

**DOI:** 10.5041/RMMJ.10184

**Published:** 2015-01-29

**Authors:** Elena Segal, Yacov Baruch, Rimma Kramsky, Marina Nudelman, Marina Hefez-Kustanovich, Sophia Ish-Shalom

**Affiliations:** 1 Metabolic Bone Diseases Unit, Rambam Health Care Campus, Haifa, Israel;; 2 The Bruce Rappaport Faculty of Medicine, Technion-Israel Institute of Technology, Haifa, Israel; 3 Liver Unit, Rambam Health Care Campus, Haifa, Israel

## Abstract

**Introduction:**

Twelve years ago we evaluated 29 liver transplant patients. Nineteen (65.5%) had decreased bone mass, 11 (37.8%) were osteoporotic, 28 (96.5%) had 25-hydroxyvitamin D (25-OHD) serum levels <20 ng/ml, mean 12.52±3.19, mean parathyroid hormone (PTH) 59.67±29.78 pg/ml. None of the patients were treated with calcium or vitamin D supplements, in spite of their low calcium intake and low or suboptimal 25-OHD serum level. All patients received letters containing their diagnoses and treatment recommendations.

This study aimed at evaluating the metabolic status of the same patients 12 years later.

**Patients and Methods:**

The patient’s charts were re-evaluated. Nineteen patients remained under follow-up at the Liver Unit: aged 58.8±15.8, 9 men and 10 postmenopausal women. Ten patients had died, none of them had fractures. Twelve fractures were reported in the surviving patients following transplantation, 10 (83.3%) within the first year, 2 in the following years, both were traumatic.

**Results:**

Repeat bone mineral density (BMD) results were available for 8 (42%) patients: 2 (25%) were osteoporotic and 6 (75%) were osteopenic. Thirteen patients (68.4%) received 600 mg of elemental calcium, 400–1600 IU of vitamin D daily, 2 (10.5%) received bisphosphonates.

Plasma PTH ranged from 32–71 pg/ml, mean 61.9±26.42; 25-OHD range was 14–22, mean 24.72±9.49 ng/ml; p=0.068. These results represented an increase from their first assessment 12 years ago: Plasma PTH 59.67±29.78 pg/ml; 25-OHD 12.52±3.19 ng/ml; p=0.003.

Since the first evaluation, 20 new post-liver transplant patients were admitted to the Rambam Liver Unit. They were not evaluated in the Bone Metabolism Unit. According to the information retrieved from their charts, 7 underwent BMD evaluation. Of these, one woman was in the osteoporotic range and 6 were osteopenic, with PTH of 82.83±67.01 pg/ml and 25-OHD of 23.95±8.7 ng/ml.

Nine (45%) of the 20 are currently being treated with calcium 600 mg/day and vitamin D, 400–2000 IU/day, and two are receiving oral bisphosphonates. No fractures have been reported by these patients since the time of transplantation.

Overall, the PTH was 71.82±49.64 pg/ml and the 25-OHD was 25.11±9.31ng/ml for the entire group of patients (39).

**Conclusions:**

Osteoporotic fractures in liver transplant patient occurred during the first post-transplantation year. No osteoporotic fractures were observed during subsequent long-term follow-up. Vitamin D deficiency and low calcium intake were actively treated and secondary hyperparathyroidism was not observed during long term follow-up. A BMD follow-up was not routinely performed in all patients. No significant change in BMD was observed during long-term follow-up in the tested group.

# Sub article: Poster #078: Results of Secondary Fracture Prevention Program in Patients with Severe Osteoporosis in Rambam Health Care Campus

**DOI:** 10.5041/RMMJ.10184

**Published:** 2015-01-29

**Authors:** Sofia Ish-Shalom, Elena Segal, Marina Hefetz, Marina Nodelman, Doron Norman

**Affiliations:** 1 Metabolic Bone Diseases Unit, Rambam Health Care Campus, Haifa, Israel;; 2 The Orthopedic Surgery Department, Rambam Health Care Campus, Haifa, Israel;; 3 The Ruth & Bruce Rappaport Faculty of Medicine, Technion-Israel Institute of Technology, Haifa, Israel; 3 Liver Unit, Rambam Health Care Campus, Haifa, Israel

## Abstract

**Introduction:**

The risk of additional osteoporotic fractures in patients who have undergone fragility fractures is at least six times higher than in age and gender-matched adults. The study purpose was to define the anti-osteoporotic treatment status in these high risk patients. Treatment strategies should be directed to provide intensive fracture prevention treatment to patients with osteoporotic fractures

**Patients and Methods:**

A collaborative (orthopedics and bone metabolism) fracture prevention program for patients hospitalized with osteoporotic fractures was started at Rambam Health Care Campus in 2008. All patients hospitalized with fractures were offered fracture-prevention treatment after surgical fracture repair. The study population included 1,647 patients who enrolled in the program between 2008 and 2013: median age 78 (range 23–103); 1,165 (71%) women with a median age of 78 (range 33–100) and 482 (29%) men with a median age of 77 (range 23–103).

**Results:**

Proximal femoral fractures were sustained by 1,115 (68%) of the patients: 769 (66%) women. 346 (72%) men. During the study period 256 (16%) patients died: 152 (13%) women and 104 (22%) men; hip fracture occurred in 212 patients: 123 (16%) women and 89 (26%) men. The relative risk (RR) for death in men following a hip fracture was 1.61 (95% CI 1.26–2.05) as compared to women. The major predictors of death in women were greater age, number of medications, higher creatinine level and lower albumin; in men, the risk factors were greater age, number of medications, and higher creatinine level. Prior to admission 264 (23%) women and 10 (2%) men were treated for osteoporosis, and hip fractures had occurred in 171 (22%) women and 6 (2%) men. After discharge 404 (35%) women and 42 (9%) men received anti-osteoporosis treatment; 247 (32%) of women and 31 (9%) of men received treatment for subsequent hip fractures. While no factors were related to an increased likelihood of men being treated, women were more likely to be treated if they had any previous fractures, previous anti-osteoporosis treatment, higher vitamin D or albumin, and lower creatinine. Treatment distribution for all patients was: 319 (19.3%) oral bisphosphonates; 73 (4.45%) IV bisphosphonates (Zoledronate); 2 (0.12%) pamidronate); 47 (2.87%) teriparatide; and 5 (0.31%) raloxifen.

**Conclusion:**

We conclude that in spite of a marked increase in the rate of patients treated after an index fracture, in a hospital initiated collaborative treatment program, this treatment rate is still unsatisfactory. In Israel, the use of IV bisphosphonates as the first line of therapy after a hip fracture remains quite low, even though it is an officially recognized and available treatment.

# Sub article: Poster #071: Identification of Neutralizing Anti-infliximab Antibodies

**DOI:** 10.5041/RMMJ.10184

**Published:** 2015-01-29

**Authors:** Roni Weisshof, Bella Unger, Alexandra Blatt, Aviva Dahan, Matti Waterman, Shomron Ben-Horin, Yehuda Chowers

**Affiliations:** 1 Department of Gastroenterology, Rambam Health Care Campus, Haifa, Israel; 2 Gastroenterology Department, Sheba Medical Center, Tel Hashomer, Ramat Gan, Israel

## Abstract

25-25

**Introduction:**

About 60% of infliximab (IFX) treated inflammatory bowel disease (IBD) patients develop anti-infliximab antibodies (ATI) with a sizable population suffering from loss of response (LOR). However, these antibodies are heterogeneous, some of which have no therapeutic consequences. Defining their neutralizing potential has marked therapeutic significance.

**Methods:**

A biological assay in which TNF-α induced IL-8 secretion from HT-29 cells followed by application of IFX and control pooled IBD sera, or ATI-containing sera was developed and IL-8 amplification ratio was determined relative to controls. An immune test, in which the IFX concentration was determined before and after its incubation with sera compared to control, was also developed.

**Results:**

(Figure 1): Forty-five ATI positive sera were tested. Of those, 41 sera (91%) contained neutralizing antibodies that inhibited IFX activity in both the biological and immune assays. No difference in detection of the ATI neutralizing effect was noted between assays. Using the immune assay, the mean IFX reduction ratio of 29 sera from patients with clinical LOR was 4.9±3.2, compared with 1.9±1.3 in patients without LOR (P=0.001). Notably, the ATI concentration of both groups was similar (9.5 μg/ml and 10.2 μg/ml respectively p=0.74). Using a cut-off value of 1.65 IFX reduction ratio the sensitivity and specificity for LOR were 86.2% and 66.7%, respectively (p=0.001). Antibody extraction led to loss of the neutralizing effect in the bioassay confirming its specificity. When early double negative (ADA (−) IFX (−))sera from IFX-treated patients with or without subsequent LOR were compared, the median ITBRs were 1.3 and 0.57 respectively (p=0.028).

**Conclusions:**

Two assays for detection of neutralizing ATI were developed and compared. No advantage was found for the more complex bioassay. A predictive potential of the test was identified allowing identification of patients prone to develop immunogenic LOR. These findings may assist in optimizing infliximab therapy in IBD patients.

# Sub article: Poster #074: Involvement of Heparanase in the Pathogenesis of Pancreatitis: Potential Therapeutic Target

**DOI:** 10.5041/RMMJ.10184

**Published:** 2015-01-29

**Authors:** Iyad Khamaysi, Preeti Singh, Neta Ilan, Israel Vlodavsky, Niroz Abu-Saleh, Hoda Awad, Yehuda Chowers, Zaid Abassi

**Affiliations:** 1 Department of Gastroenterology; 3 Research Unit, Rambam Health Care Campus;; 2 Cancer and Vascular Biology; 4 Department of Physiology, The Ruth & Burce Rappaport Faculty of Medicine, Technion–IIT, Haifa, Israel.

## Abstract

**Introduction:**

Despite advances in understanding the etiopathogenesis of acute pancreatitis (AP), the mechanisms underlying this acute inflammatory disease have not been fully determined. In the majority of cases AP a self-limiting process, yet 20% of patients develop a severe form of necrosis with multi-organ complications and a high mortality rate. Heparanase (Hpa), an endoglycosidase which cleaves heparan sulfate, participates in degradation and remodeling of the extracellular matrix. Heparanase is preferentially expressed in human tumors, including pancreatic adenocarcinoma. Besides its role in cancer, Hpa plays an important role in the pathogenesis of several inflammatory disorders, such as lung injury, rheumatoid arthritis, and chronic colitis. The involvement of Hpa in AP pathogenesis has not yet been addressed. Therefore, the current study uses an experimental model to examine whether Hpa is involved in the pathogenesis of cerulein-induced AP in mice.

**Materials and Methods:**

Heparanase over-expressing transgenic mice (*hpa*-TG) and their wild-type (WT) BALB/c mice, and Hpa knockout mice (*hpa*-KO) and their WT C57 mice were intraperitoneally injected with either Cerulein (50mg/kg, 5 times, at 1 hour apart) or vehicle. Pancreatic Hpa activity, edema, and inflammation along blood amylase and lipase levels, were determined 24 hours following pancreatitis induction.

**Results:**

Cerulein-induced pancreatitis in wild type mice was associated with significant rises in the serum levels of amylase and lipase. These increases were characterized by enhancement of Hpa activity and pancreatic inflammation. The elevation in amylase and lipase as well as pancreatic edema/inflammation responses to administration of cerulein were profoundly exaggerated in *hpa*-TG mice. In contrast, when cerulein was injected to *hpa*-KO mice, the severity of pancreatitis was attenuated as compared with their wild type controls. Interestingly, pretreatment with selective Hpa inhibitor reduced significantly the inflammatory response of acute pancreatitis by ameliorating pancreatic edema, amylase, and lipase serum levels.

**Conclusions:**

The *hpa*-TG mice are more susceptible to acute pancreatitis than their WT controls, suggesting a role for Hpa in the pathogenesis of these disease states. The pancreatic-protective effects of Hpa inhibition provide a rational basis for therapeutic application of Hpa inhibitors.

# Sub article: Poster #059: Use of Technology to Preserve Cognitive Function in the Aging Brain

**DOI:** 10.5041/RMMJ.10184

**Published:** 2015-01-29

**Authors:** Tzvi Dwolatzky, Vardit Sarne-Fleischmann, Noam Tractinsky

**Affiliations:** 1 Geriatric Service, Rambam Health Care Campus, Haifa, Israel;; 2 Faculty of Health Sciences, Ben Gurion University of the Negev, Beersheva, Israel; 3 The Faculty of Engineering, Ben Gurion University of the Negev, Beersheva, Israel

## Abstract

**Introduction:**

Aging of the population is associated with a marked rise in the number of older people with cognitive impairment. Pharmacological interventions for cognitive decline have limited efficacy and are, at best, symptomatic. Psychosocial and lifestyle interventions, which aim primarily to relieve cognitive and behavioral symptoms, also promote well-being and provide caregiver support. Increasingly, computerized systems are being designed for the treatment of patients with dementia, including web sites for providing information, computerized tools for monitoring, assistive technology and computer systems offering emotion-oriented treatments in dementia care. We evaluated two computer-supported interventions for patients with cognitive impairment, namely personalized computerized reminiscence therapy and computerized cognitive training.

**Materials and Methods:**

A total of 167 older subjects with cognitive impairment or dementia, residing in the community, were recruited for the study. All underwent a preliminary assessment to determine their cognitive status. Following initial screening 95 suitable subjects were randomly assigned to one of the following three groups: (1) Personal computer-based reminiscence therapy; (2) Computer-based cognitive training; and (3) Control group. All subjects participated in two sessions a week, each of 30-minutes duration, supervised by a mediator, for a period of 3 months, and controls continued with usual activities. Assessments were performed at baseline, at one month, and at 3 months. They included an evaluation of cognitive function using the Neurotrax computerized testing battery, psychological and behavioral well-being using Quality of Life in Alzheimer’s Disease (QoL-AD), Will to Live (WTL) and Neuropsychiatric Inventory (NPI) questionnaires, and caregiver burden using the short version of Zarit Caregiver Burden Interview.

**Results:**

A total of 85 participants completed at least one follow-up evaluation. No differences between groups were found at baseline. For the reminiscence group, beneficial effects were found for the global cognitive score, QoL-AD-patient, and Will To Live. For the cognitive training group modest effects were found for orientation, verbal function, and Go-No-Go reaction time.

**Conclusions:**

This study found positive results for both computer-based reminiscence and cognitive training in older subjects with cognitive impairment. Due to the limited sample size the effects demonstrated were not adequate to draw firm conclusions. We suggest conducting additional randomized controlled trials with greater sample sizes and longer evaluation periods.

# Sub article: Poster #023: Microparticles Characterization in Patients with Acute Leukemia at Diagnosis and After Induction Remission Treatment*

**DOI:** 10.5041/RMMJ.10184

**Published:** 2015-01-29

**Authors:** Tzoran Inna, Benjamin Brenner, Aharon Anat

**Affiliations:** 1 Department of Internal Medicine C, Rambam Health Care Campus, Haifa, Israel;; 2 Hematology Institute, Thrombosis and Haemostasis Unit, Rambam Health Care Campus, Haifa, Israel; 3 The Ruth & Bruce Rappaport Faculty of Medicine, Technion-Israel Institute of Technology, Haifa, Israel

## Abstract

**Introduction:**

Microparticles (MPs) are membrane vesicles shed from various cells and may express antigens that reflect their cellular origin. Tissue factor (TF)-bearing MPs plays a major role in the pathogenesis of the prothrombotic state observed among patients with malignant disease. Acute myeloid leukemia (AML) is characterized by rapid growth of abnormal blast cells. Patients with AML can develop venous thromboembolism despite thrombocytopenia. We hypothesize that circulating MPs may serve as biomarker, reflect the change in blood cell population, and predict the thrombogenic state of AML patients at diagnosis and at remission.

**Patients and Methods:**

Blood samples were collected from healthy controls and from patients with newly diagnosed AML at three time points: diagnosis, at nadir, and at remission achievement. The cell origin of MPs was characterized by specific fluorescent antibodies and analyzed by FACS. The MPs were labeled for various antigens including CD34, CD33 –blast leukemic cells markers. To determine the procoagulant and anticoagulant potential, each sample was labeled with florescent antibodies against TF and tissue factor pathway inhibitor (TFPI). The procoagulant activity of MPs was also evaluated by FXa chromogenic assay.

**Results:**

Forty-two patients with AML were enrolled in the study. Platelet markers were significantly higher in controls compared to rates at diagnosis of AML (30.7% vs. 10.3%; p<0.05), with no change in the markers for activated platelets. CD34 as a blast cell marker was significantly higher in patients at diagnosis as compared to controls (4.4% vs. 1.4%; p<0.001) and to patient MPs at remission (4.4% vs. 1.7%; p<0.05). CD33 as another blast cell marker was also found to be significantly higher in patients at diagnosis as compared to controls (34.9% vs. 15.3%; p<0.05) and to remission (34.9% vs. 15.7%; p<0.01). Procoagulant activity was significantly higher in patients at diagnosis and at remission compared to healthy controls (100.5 AU vs. 58.5 AU; p<0.05, and 135 AU vs. 58.5 AU; p<0.001, respectively).

**Conclusion:**

The MPs of AML patients at diagnosis express blast cell markers and may serve as a biomarker for disease and for remission. Increase of endothelial MPs at AML diagnosis may point to vascular injury, which may also result in an increase of TF and a reduction of TFPI, with an overall increase in patient thrombogenicity. The AML-MPs reflect a patient’s disease and my serve as a “red flag” for a hypercoagulable state.

# Sub article: Poster #025: Heterogeneity of Acute Myeloid Leukemia as a Contributing Factor for Relapse Using Single Cell Analysis Approach

**DOI:** 10.5041/RMMJ.10184

**Published:** 2015-01-29

**Authors:** Shlomit Yehudai-Reshef, Tsila Zuckerman

**Affiliations:** Department of Hematology, Rambam Health Care Campus, Haifa, Israel; 2 The Ruth & Bruce Rappaport Faculty of Medicine, Technion-Israel Institute of Technology, Haifa, Israel

## Abstract

**Introduction:**

Acute Myeloid leukemia (AML) is associated with poor outcome mainly due to relapse. There is substantial evidence that AML, like other cancers, is a heterogeneous disease composed of different subclones. Intraclonal genetic diversity reflects natural selection which may lead to clonal evolution, disease progression, relapse, or metastasis. Delineation of the tumoral genomic landscape is mandatory for demonstrating the presence of heterogeneous leukemic populations. We hypothesized that AML heterogeneity may contribute to relapse through several possible mechanisms such as: clonal evolution, presence of dormant cells escaping treatment and/or abnormal interaction with the micro-environment (ME) supporting leukemic subclones expansion.

**Materials and Methods:**

Most studies use bulk DNA for genotyping the tumor; however, this approach can miss rare subclones which could eventually lead to relapse. Therefore, in order to investigate the role of different subclones in disease progression, we studied AML associated mutations using single cell analysis (SCA).

**Results:**

Using this unique approach, we were able to show that patients, considered negative for the FLT3-ITD AML mutation, when tested by bulk DNA, actually possessed a small fraction of this mutation. The presence of a minor clone carrying FLT3-ITD strongly suggests that this lesion is a common late event in leukemogenesis. Additionally, further clonal evolution was shown in the FLT3 gene by the accumulation of different mutations in the same patient and loss of heterozygosity of wild type allele in 25–100% of tested cases.

Combining single leukemic cell genetic analysis at diagnosis and relapse with clonal hierarchy as determined by the mutation rate in 200 micro-satellites of single cells, we were able to identify the presence of dormant cells that may evade chemotherapy due to their dormancy, as well as the appearance of mutations in mismatch repair genes contributing to relapse.

Subclones derived from single cells were subjected to various combinations of drugs and differential chemosensitivity was observed, further supporting the significance of tumor heterogeneity.

**Conclusions:**

This comprehensive study suggests that relapse mechanisms interplay between intrinsic genetic properties of various clones, together with micro-environmental alterations supporting tumor expansion. Understanding mechanisms of disease relapse may promote targeted therapy and also serve as a model for investigating progression of other cancers.

# Sub article: Poster #049: The Effect of Immunomodulatory Agents on Mantle Cell Lymphoma Cell Survival and Microenvironment Interactions

**DOI:** 10.5041/RMMJ.10184

**Published:** 2015-01-29

**Authors:** Shoham Shivtiel-Arad, Noam Bettman, Irit Avivi, Netanel Horowitz, Noam Benyamini, Tami Katz

**Affiliations:** Department of Hematology and Bone Marrow Transplantation, Rambam Health Care Campus, Haifa, Israel

## Abstract

**Introduction:**

Mantle cell lymphoma (MCL) is a rare disease, characterized by accumulation of malignant B cells in different lymphatic organs. Although MCL remains an incurable disease, the emerging data regarding Lenalidomide (Len) activity and the employment of Rituximab (RTX) therapy appears to significantly contribute to improvement in patient survival. Moreover, combined therapy with these two immunomodulators might result in superior survival. Therefore, we aimed to explore the effect of these drugs on possible alterations in the patient’s immune system in order to gain more insight into their mechanisms of action. In addition, recent data suggest that malignant cell survival is also dependent on the surrounding stromal cells involved in drug resistance and immunotolerant environment. Therefore, it seems highly important to test the effect of different immunomodulatory agents also in the context of the lymphoma microenvironment.

**Materials & Methods:**

Peripheral blood mononuclear cells (PBMCs) obtained from both healthy donors and MCL patients were incubated *in vitro* for 5 days with Len, RTX, and a combination of these drugs. The PBMCs were stained and analyzed by FACS to monitor alteration in activation status of different immune components. To evaluate adhesion molecule expression, the MCL line (Granta 519) was co-cultured with stromal cells obtained from bone marrow (BM. HS-5 line) and lymph nodes (LN. HK cells) in the presence of Len. The VLA4 adhesion molecule level was analyzed by FACS. The MCL line (mino), pre-treated with Len, was tested in a transwell assay to assess cell migration potential through BM vs. LN stromal cells towards SDF-1.

**Results:**

We found that combined treatment (Len+RTX) had a synergistic effect on activating immune cells in the culture. Effector cells like NK cells and T lymphocytes showed increased activation of markers and secretion of cytokines (such as INF-γ). Additionally, we detected reduction in the mechanisms involved in immune inhibition, such as a decrease in regulatory T-cell population and down-regulation in PD1 expression. Next, we characterized various players involved in the interaction between lymphoma cells and their supporting environment, comparing different sources of stromal cells obtained from BM vs. LNs. Co-culture of MCL lines and stromal cell lines revealed the following:

1. Interaction with LN stromal cells, but not BM stroma, induced the expression of adhesion molecules on MCL line regulating their motility and survival signals.
2. The ability of lymphoma cells to migrate through the stromal barrier increased over both stromal cells. However, pre-treatment of MCL cells with Len inhibited cell migration through BM cells, but not through LN stromal cells.

**Conclusions:**

These promising *in vitro* results may indicate a possible advantage in using combined treatment in MCL patients to promote enhanced immune responses leading to an efficient eradication of lymphoma cells. In addition, our co-culture results demonstrate that the interaction between lymphoma and stromal cells induces alteration in cell properties that are environment-dependent, regulated by distinctive interactions in each lymphoma compartment, suggesting a preferential effect on drug therapy and immune response against lymphoma cells.

# Sub article: Poster #004: Prospective Screening for Anti-platelet Antibodies and Immune Platelet Refractoriness in Patients Diagnosed with Acute Myeloid Leukemia

**DOI:** 10.5041/RMMJ.10184

**Published:** 2015-01-29

**Authors:** Roy Lauterbach, Nuhad Haddad, Lilach Bonstein

**Affiliations:** 1 Blood Bank and Platelet Immunology Laboratory, Rambam Health Care Campus, Haifa, Israel; 2 The Department of Hematology and BMT, Rambam Health Care Campus, Haifa, Israel

## Abstract

**Introduction:**

Acute myeloid leukemia (AML) patients are treated with intensive chemotherapeutic protocols, suffer from protracted pancytopenias, and need prolonged transfusion support. Long-term transfusion dependency exposes these patients to alloimmunization to both leukocyte (HLA) and platelet (HPA) antigens, expressed by immune platelet transfusion refractoriness, a major risk factor for bleeding-associated morbidity and mortality. The published incidence of platelet refractoriness in patients with hematologic malignancies ranges between 7% and 34%. There is no data regarding the incidence of platelet refractoriness in the Israeli population. The aims of the study were: to estimate the prevalence of platelet transfusion refractoriness among Israeli AML patients, to define the “timing” of alloimmunization during treatment protocol, and to evaluate weekly antibody screening as a tool to predict the risk for immune transfusion refractoriness.

**Patients and Methods:**

One hundred newly diagnosed AML patients (55 males and 45 females) between 25–60 years of age, from of various ethnic origins were included. The newly diagnosed AML patients were screened for the presence of anti-platelet antibodies and anti-HLA antibodies using flow cytometry. Antibodies were identified by the MAIPA assay. All patients were followed using weekly antibody screening. Clinical parameters of patients were consecutively registered. Patients received platelet transfusions according to the local transfusion policy with continuous PLT increment monitoring. Patient blood counts and platelet transfusion requirements were followed weekly, platelet refractoriness was determined when no increment was documented on two consecutive platelet transfusions.

**Results:**

Platelet refractoriness was found in 50 (50%) patients (21 males and 29 females; 44 patients (44%) developed new anti HLA and/or anti HPA antibodies, 32/44 (73%) were females. Immune platelet refractoriness was defined in 34/50 (68%) refractory patients (26 females and 8 males). For patients with antibodies, 34/44 (77%) developed refractoriness; 13/34 had anti-HPA with (11) or without (2) anti-HLA antibodies. Only anti-HLA antibodies were found in 21/34 patients. Average time to antibody production was 28 days from the beginning of treatment protocol.

**Conclusions:**

Our results demonstrate that anti-HPA antibodies are strongly correlated with the appearance of immune platelet refractoriness and that the prevalence of immune platelet refractoriness in Israeli AML patients is higher than expected according to published data.

# Sub article: Poster #042: The Role of Pregnancy-induced Hormonal Milieu in Lymphoma Progression

**DOI:** 10.5041/RMMJ.10184

**Published:** 2015-01-29

**Authors:** Netanel Avraham Horowitz, Noam Bettman, Ali Abed El Wahab, Irit Avivi, Tami Katz

**Affiliations:** The Department of Hematology and Bone Marrow Transplant, Rambam Health Care Campus, Haifa, Israel

## Abstract

**Introduction:**

Lymphoma is the most common hematological cancer reported during pregnancy. Recent data suggest that unlike lymphoma occurring outside of pregnancy, pregnancy-associated lymphoma is characterized by an excessive involvement of reproductive organs, advanced disease stage at diagnosis and an aggressive course, potentially leading to a high death rate of mothers.

**Objective:**

This study was aimed at determining the role of estrogen and progesterone in lymphoma progression *in vitro.*

**Materials and Methods:**

Several human lymphoma cell lines were analyzed by FACS for expression of estrogen (α & β) and progesterone receptors. Human breast cancer cell line Mfc-7 was used as a positive control. CFSE-stained BL2 cells, were treated with E_2_ (25μM and 100μM) for 24h and 48h. Cells were harvested and analyzed by FACS for determining cell proliferation. Ramos cells were treated with E_2_ (5–25μM) for 72h. Cells were harvested and stained with Annexin-PI for determining live/necrotic/apoptotic cell proportion. The BL2 cells, either seeded alone or co-cultured with HK cells for 24h, were treated with E_2_ (25μM & 100μM) for 24h. The proportion of live, necrotic, and apoptotic cells was also analyzed.

**Results:**

Lymphoma cell lines express both estrogen receptors but not progesterone receptors. Estrogen-treated lymphoma cells demonstrated a reduced proliferation rate in a concentration-dependent manner. Furthermore, apoptosis and necrosis of lymphoma cells were significantly increased with concentration-dependent estrogen treatment. Progesterone had no effect on lymphoma cells proliferation, apoptosis or necrosis. The negative effect of estrogen on lymphoma cells was reversed in the presence of lymph node stromal cells. Estrogen treated lymphoma cells that were cultured on lymph node stromal cells demonstrated significantly less apoptosis and necrosis rates relative to non-cultured cells.

**Conclusion:**

Lymphoma cells express estrogen but not progesterone receptors. Estrogen induces lymphoma cell necrosis and apoptosis and reduces proliferation. Lymph node stromal cells protect lymphoma cells from estrogen mediated effects. The direct effect of estrogen on lymphoma cells does not support lymphoma progression. Other mechanisms should be sought to explain the aggressive nature of lymphoma diagnosed during pregnancy.

# Sub article: Poster #046: Plasma-sHLA-bound Peptides in Acute Myeloid Leukemia as Predictors of Individual Response and Their Application in Immunotherapy

**DOI:** 10.5041/RMMJ.10184

**Published:** 2015-01-29

**Authors:** Chen Itzkovich, Michal Hayun, Eilon Barnea, Arie Admon, Yishai Ofran

**Affiliations:** 1 The Ruth & Bruce Rappaport Faculty of Medicine, Technion-Israel Institute of Technology, Haifa, Israel; 2 Hemato-Oncology Research Lab, Rambam Health Care Campus, Haifa, Israel; 3 Department of Biology, Technion-Israel Institute of Technology, Haifa, Israel

## Abstract

**Introduction:**

Myeloid leukemia cell population is heterogeneous and composed of different immunophenotypically sub-populations. These leukemic sub-populations may also vary in their sensitivity to chemotherapy. During treatment, inherent chemo-resistant sub-populations survive and are becoming a dominant clone.

It has shown that soluble HLA (sHLA) -bound peptides molecules present in the plasma of cancer patient are mostly derived from the disease cells and thus allow the identification of sHLA peptidomes.

Here we analyzed and compared differences in the expression of sHLA peptides between boon marrow (BM) plasma at: diagnosis, during chemotherapy induction and remission, as well as normal healthy population, to find a peptide model which will be used to measure the response of each patient to the treatment and map out disease-specific peptide spectrum, clarify the possible pathogenesis, resistance mechanism, and disease prognosis.

**Materials and Methods:**

Plasma from BM aspirates samples derived from acute myeloid leukemia (AML) patients will be extracted during the follow time points: at diagnosis (D1), fifth day (D5) and day fourteen (D14) from induction therapy. sHLA and peptides purification from plasma: samples are loaded sHLA class I molecules with their bound peptides loaded on pretreated monoclonal antibody W6/32. Elution is done with Trifluoroacetic acid and the sHLA is separated from their peptides on C18 Micro TipColumn with acetonitrile. The peptides fraction is concentrated by vacuum centrifugation until complete evaporation and are examined in liquid chromatography coupled mass spectrometry (LC-MS/MS).

**Results:**

Peptides repertoire bound to sHLA-plasma were identified by LC-MS. The peptides from diagnosis (D1), during chemotherapy treatment (D5) and remission (D14) was compared in eleven AML patients. We found that during chemotherapy (D5) there was an elevation of 25 peptides related to a variety of cancers genes and to proteins involved in different cell biological processes. These peptides were correlated with patients who did not respond to chemotherapy treatment.

**Conclusions:**

In this study we found peptides unique to AML which did not exist in healthy donors, and also found a difference in the appearance of those peptides during chemotherapy treatment compared with pre-treatment. Furthermore, we found a correlation between specific peptides and patient responses to treatment.

# Sub article: Poster #006: Histomorphometric Findings May Help Predict Response To Antiviral Therapy at an Early Fibrosis Grade in Patients with Chronic HCV Infection

**DOI:** 10.5041/RMMJ.10184

**Published:** 2015-01-29

**Authors:** Mayson Darawshi, Amir Klein, Tarek Saadi, Edmond Sabo, Yaacov Baruch

**Affiliations:** 1 Liver Unit; 2 Department of Gastroenterology; 3 Department of Pathology, Rambam Health Care Campus, Haifa, Israel; 4 The Ruth & Bruce Rappaport Faculty of Medicine, Technion-Israel Institute of Technology, Haifa, Israel

## Abstract

**Introduction:**

Hepatitis C virus (HCV) is a leading cause of cirrhosis and hepatocellular carcinoma worldwide. Several viral and host factors related to viral response have been reported. Morphometry is a field that investigates changes in shape, size and orientation of objects and allows for the quantification of these parameters, which can highlight areas with significant differences. This study had two aims: 1. Quantification of histological findings from patients with chronic HCV using computerized morphometrics; and 2. Prediction of response to medical treatment of chronic HCV using baseline histomorphometric findings.

**Patients and Methods:**

Fifty-eight patients with chronic HCV infection with a Metavir score F1 and F2, and followed in our Liver Unit were selected and grouped according to treatment response: sustained viral response (SVR) and non-SVR. Histomorphometric analysis was blinded to patient identification or previous histological information.

Slides were scanned using the dot slide virtual microscopy (Olympus) system. The entire slide was manually scanned and 3–4 representative images were recorded from each one. Each biopsy contained 6–8 representative portal spaces in average. The Imagepro Plus 0.0 (MediaCybernetics, Rockville, MD, USA) software was used to analyze and quantify collagen fibers, inflammatory cells, and liver architecture. MATLAB (MathWorks®, Natick, MA, USA) software was used to analyze fractal and lacunar dimension, giving an indication of the architectural distortion in the liver parenchyma.

**Results:**

Clinical parameters, including age, white blood cell count and hemoglobin concentration, and histomorphometric variables, including the density of collagen fibers, the number of inflammatory cells in the portal space, and textural parameters were found to be statistically significant and could be used together as a formula to predict response to treatment in HCV patients, with 93% sensitivity and 100% specificity.

**Conclusions:**

The histomorphometric method is promising and may contribute to developing an expert guided automatic system predicting response to treatment in chronic HCV patients. Morphometry may be used in the future to investigate liver diseases due to different etiologies.

# Sub article: Poster #010: A Novel Truncating Mutation in *DGKE* Underlies Autosomal Recessive Atypical Hemolytic Uremic with Normo-complementemia and Partial Response to Plasma Infusion

**DOI:** 10.5041/RMMJ.10184

**Published:** 2015-01-29

**Authors:** Shirley Pollack, Israel Eisenstein, Irit Krause, Miriam Davidovits, Ayala Ophir, Daniella Magen

**Affiliations:** 1 Pediatric Nephrology Institute, Rambam Health Care Campus, Haifa, Israel;; 2 Institute of Pediatric Nephrology, Schneider Children’s Medical Center, Petach-Tikva, Israel; 3 Laboratory of Molecular Medicine, The Ruth & Bruce Rappaport Faculty of Medicine, Technion-Israel Institute of Technology, Haifa, Israel

## Abstract

**Introduction:**

Atypical hemolytic-uremic syndrome (aHUS) is a rare thrombotic microangiopathy (TMA), attributed to complement dysregulation. Recently, loss-of-function mutations in Diacylglycerol Kinase, Epsilon (*DGKE*) have been implicated in early-onset aHUS. Since *DGKE* is not an integral component of the complement cascade, the mechanism whereby its disruption induces TMA remains unclear. Moreover, there is conflicting evidence regarding the role of complement-modulating therapy, including plasma infusion, in *DGKE*-associated glomerulopathy. We report the phenotypic and genetic characteristics of a consanguineous Muslim-Arab family with aHUS due to *DGKE* disruption.

**Patients and Methods:**

Three males with early-onset aHUS and 16 of their close relatives were clinically and genetically evaluated. Genetic analysis was performed using whole exome sequencing, followed by conventional Sanger sequencing for mutation verification.

**Results:**

Patients presented at infancy with normocomplementemic aHUS, nephrotic syndrome and hypertension. Renal biopsies demonstrated glomerular migroangiopathy. Patient 1, currently 27 years-old, was not managed with plasma infusions, and developed end stage renal disease (ESRD) at 20 years of age. Patients 2 and 3, currently 8- and 6-years-old, respectively, demonstrated clinical response to plasma exchange/infusions during aHUS relapses, and maintained prolonged remission under prophylactic plasma infusions. Temporary cessation of prophylactic plasma infusions and switch to eculizumab therapy in patient 3, resulted in aHUS relapses, which resolved after re-introduction of monthly plasma infusions. Exome sequencing excluded disruption of all currently known genes involved in aHUS, but revealed a novel homozygous nonsense mutation in *DGKE* (p.337R>X) of all affected individuals, predicted to truncate the catalytic domain of the protein. The p.337R>X mutation was confirmed by Sanger sequencing, segregated with the disease phenotype, and was not identified in public databases or in 300 ethnically-matched control chromosomes.

**Conclusion:**

Our findings confirm that *DGKE* disruption underlies early-onset aHUS associated with a chronic glomerular microangiopathy, which is unresponsive to eculizumab therapy. Our observation of salutary therapeutic and preventive effects of plasma infusion on acute attacks of aHUS, points to consideration of plasma therapy in the management of *DGKE*-associated aHUS. The mechanism whereby plasma infusions may modify *DGKE*-associated aHUS attacks, as well as the long-term effect of plasma therapy on disease progression remain to be established.

# Sub article: Poster #040: Induced Pluripotent Stem Cells as a Model for Telomeric Abnormalities in ICF Type I Syndrome

**DOI:** 10.5041/RMMJ.10184

**Published:** 2015-01-29

**Authors:** Shira Sagie, Erika Ellran, Hagar Katzir, Rony Shaked, Shiran Yehezkel, Alaa Ghanayim, Karl Skorecki, Maty Tzukerman, Sara Selig

**Affiliations:** 1 Molecular Medicine Laboratory, Rambam Health Care Campus, and The Ruth & Bruce Rappaport Faculty of Medicine and Research Institute, Technion-Israel Institute of Technology, Haifa, Israel; 2 The Computer Science Department, Technion-Israel Institute of Technology, Haifa, Israel

## Abstract

**Introduction:**

Human telomeric regions are packaged as constitutive heterochromatin, characterized by extensive subtelomeric DNA methylation and specific histone modifications. Immunodeficiency, centromeric instability, facial anomalies (ICF) syndrome type I patients carry mutations in DNA methyltransferase 3B (DNMT3B) that methylates de novo repetitive sequences during early embryonic development. The fibroblasts of ICF type I patients display hypomethylated subtelomeres, abnormally short telomeres, and premature senescence.

**Materials and Methods:**

In order to study the molecular mechanism by which the failure to de novo methylate subtelomeres results in accelerated telomere shortening, we generated induced pluripotent stem cells (iPSCs) from three ICF type I patients.

**Results:**

Telomeres were elongated in ICF-iPSCs during reprogramming, and the senescence phenotype was abolished despite sustained subtelomeric hypomethylation and high TERRA levels. Fibroblast-like cells isolated from differentiated ICF-iPSCs maintained abnormally high TERRA levels, and telomeres in these cells shortened at an accelerated rate, leading to early senescence, thus recapitulating the telomeric phenotype of the parental fibroblasts.

**Conclusions:**

These findings demonstrate that the abnormal telomere phenotype associated with subtelomeric hypomethylation is overridden in cells expressing telomerase, therefore excluding telomerase inhibition by TERRA as a central mechanism responsible for telomere shortening in ICF syndrome. The data in the current study lend support to the use of ICF-iPSCs for modeling of phenotypic and molecular defects in ICF syndrome and for unraveling the mechanism whereby subtelomeric hypomethylation is linked to accelerated telomeric loss in this syndrome.

# Sub article: Poster #041: The Israeli Experience of the First 300 Panorama™ Tests that Use 19,488 Single Nucleotide Polymorphisms (SNPs) Followed by High-throughput Sequencing for Common Trisomies Risk Assessment

**DOI:** 10.5041/RMMJ.10184

**Published:** 2015-01-29

**Authors:** Hagit N. Baris, Zeev Weiner, Ido Solt, Mordechai Shohat, Doron M. Behar

**Affiliations:** 1 The Genetic Institute; 2 Department of Obstetrics and Gynecology, Rambam Health Care Campus, Haifa, Israel; 3 The Recanati Genetic Institute, Rabin Medical Center, Petach Tikva, Israel

## Abstract

**Background:**

Cell free DNA (cfDNA) has emerged over the last year as an alternative for amniocentesis for diagnosis of the common aneuploidies looking at trisomy 21, 13, 18, sex chromosomes and triploidy.

**Materials and Methods:**

We present our experience of the first 300 Panorama™ tests sent from Israel. This method is based on massively multiplexed PCR amplification of cfDNA isolated from maternal plasma, targeting 19,488 SNPs, followed by high-throughput sequencing. The fetal fraction is determined. The SNP pattern of maternal DNA (from buffy coat) is compared to the SNP pattern of free DNA from maternal plasma, which contains maternal and fetal DNA. Paternal genomic samples, when available, were included in the analysis; in the absence of a paternal sample, the algorithm considers population allele frequencies. Combining the maximum likelihood ratio with *a priori* risk generates a risk score.

**Results:**

The results of the first 300 sequential tests performed in Israel were analyzed. Fifteen samples necessitated redraw, two samples failed analysis. Four samples yielded high risk scores: two cases for trisomy 21, one for Kleifelter syndrome (KS) (47,XXY), and one for trisomy 18. Confirmation of both trisomy 21 and one KS were done by CVS or amniocentesis. The mother of suspected trisomy 18 was not interested in invasive testing in view of normal ultrasound scans and delivered a healthy baby. There are no known false negative results.

**Discussion:**

Panorama™ test is a reliable tool for identification of pregnancies at high risk for fetuses with the common aneuploidies with a high success rate. We recommend confirmation of the diagnosis for high risk scores pregnancies using invasive tests.

# Sub article: Poster #058: Genitopatellar-like Syndrome in an Infant Born to Consanguineous Parents

**DOI:** 10.5041/RMMJ.10184

**Published:** 2015-01-29

**Authors:** Tova Hershkovitz, Alina Kurolap, Adi Mory, Danit Oz-Levi, Hagit N. Baris-Feldman

**Affiliations:** 1 The Genetics Institute, Rambam Health Care Campus, Haifa, Israel; 2 The Department of Pediatrics, Rambam Health Care Campus, Haifa, Israel

## Abstract

**Introduction:**

A 45-day old infant, the first child to parents who are first degree cousins of Muslim origin, was referred to the Genetics Institute at Rambam Health Care Campus due to multiple defects. The infant presented with hypertonicity, seizures, bilateral absent patellae, femoral epiphysiolysis, contractures of the lower extremities and retrognathia. Mild expansion of the lateral cerebral ventricles and mild hydronephrosis were also observed. No genital anomalies were noted. Follow up showed severe psychomotor delay. Genitopatellar syndrome (GPS) was suggested as the primary differential diagnosis.

Genitopatellar syndrome is a rare disorder, characterized by skeletal defects, most prominently hypoplastic or absent patellae, combined with urogenital anomalies, such as hypoplastic scrotum and micropenis in males or clitoral hypertrophy in females. Microcephaly, severe intellectual disability, characteristic craniofacial dysmorphism, and flexion contractures of the lower extremities had also been described. This is an autosomal dominant syndrome that is caused by de novo heterozygous mutation in *KAT6B* gene. Due to a phenotype not entirely consistent with GPS and possible autosomal recessive inheritance due to consanguinity, whole-exome sequencing was the diagnostic method of choice.

**Materials and Methods:**

Following informed consent by the parents, whole-exome sequencing was conducted on the child’s genomic DNA. Initial analysis included inspection of variants found in *KAT6B*, the candidate gene, followed by Sanger sequencing of exons with low coverage. Only after excluding potential pathogenic variations in *KAT6B*, bioinformatic analysis will be directed to other variations uncovered by exome sequencing.

**Results:**

Exome analysis revealed a heterozygous missense variant, caused by a G-to-T alteration in the first nucleotide of exon 16 of *KAT6B* (NM_001256468.1:c.2473G>T;p.Ala825Ser), but it was revoked by Sanger sequencing. No other pathogenic variants were observed in the gene. Since exons 5 and 15 exhibited low coverage, we followed with sequencing that is now pending.

**Conclusions:**

When attempting genetic diagnosis of cases with an unclear clinical presentation, we are facing a dilemma whether to Sanger sequence candidate genes or directly apply whole-exome analysis. The growing availability and lowering prices of whole-exome sequencing are directing the global trend to make it the diagnostic method of choice, both in research and in clinical settings. Nevertheless, a full clinical assessment is critical in guiding data analysis towards the causative variant.

# Sub article: Poster #020: Validation of a New Index for Pain Monitoring - NoL™ - During Surgery and Anesthesia

**DOI:** 10.5041/RMMJ.10184

**Published:** 2015-01-29

**Authors:** Ruth Edry, Vasile Recea, Yuri Dikust, Yeshayahu Katz

**Affiliations:** Department of Anesthesia, Rambam Health Care Campus, Haifa, Israel

## Abstract

**Introduction:**

Patients under anesthesia cannot communicate pain; hence an objective monitoring system is needed to assess intra-operative nociception and guide proper treatment. Based on a combination of physiological parameters, the Nociception Level (NoL) index was developed (0–100 scale). Random Forest Regression, capable of dealing with large numbers of input parameters arising from the complexity of the autonomous nervous system and their time derivatives, was applied to reveal their non-linear relations to the nociceptive state.

**Study Aim:**

The aim of this study was to validate the NoL index during surgery and anesthesia and to compare its performance to other currently used nociception related monitors.

**Patients and Methods:**

The study recruited 69 adults scheduled for surgery under general anesthesia (GA). After induction of GA (Entropy<60), all study subjects were subjected to two identical tetanic stimulations, first without analgesia (TET1) and again after 2mcg/kg fentanyl (TET2). All subjects were subjected to intubation (TP1) under the same analgesic conditions. After intubation study subjects were randomly divided into two groups of remifentanil TCI: 2 and 4 ng/ml. TET1, TET2, intubation (TP1), skin incision/first trocar insertion (TP2), and no pain (TNP) were annotated for analysis. Parameters recorded for analysis were NoL, heart rate (HR), plethysmograph amplitude (PPGA), and surgical plethysmograph index (SPI). Three values were analyzed for each parameter: pre-stimulus, post-stimulus, and reaction median values. Positive study outcomes were a significant reaction to noxious stimuli and an insignificant reaction to non-noxious stimuli; discrimination between noxious and non-noxious stimuli with AUC>0.8; grading of response to stimuli as expected (TET1>TET2; TP1>TP2> TNP); and reflection of different analgesic dosing (p-value<0.00625 after Bonferroni correction).

**Results:**

Fifty-eight subjects were valid for analysis. The NoL changed with statistical significance in response to both TET1 and TET2 (p=0.0006, p=0.0051, respectively). The SPI failed to identify TET1 but changed with statistical significance in response to TET2 (p=0.0025). The HR and PPGA failed to identify both TET1 and TET2. All parameters graded correctly the intensity of response to tetanic stimuli as: tet1>tet2. All parameters identified administration of fentanyl prior to TET2. All parameters reacted with a statistical significance to TP1 and TP2 except for the HR, which failed to respond to TP2. The only parameter to grade correctly TP1>TP2>TNP was the NoL. In ROC analysis for the ability of parameters to discriminate noxious (TP1, TP2) from non-noxious stimuli (TNP) NoL reached an AUC of 0.93 (CI 0.89–0.97), outperforming all other parameters. At a given working point of 90% specificity, NoL reached a sensitivity of 86.67% (CI: 78.64%–92.51%). The NoL post-stimulus median value was the only parameter to reflect a different basal level of remifentanil TCI during noxious stimulus (p<0.0069). The NoL reaction to TP2 reflected the different analgesic states of subjects in the high and low dose groups, with a significant reaction in the low dose group, and an insignificant reaction in the high dose group; PPGA, NIBP and SPI behaved similarly. The HR did not react with statistical significance for both low and high remifentanil doses around TP2.

**Conclusions:**

Study results validated the NoL index, demonstrating its ability to correctly identify the response to various noxious and non-noxious stimuli and to discriminate, with high sensitivity and specificity, between the two entities. Moreover, the NoL index was proved able to grade the response to noxious stimuli as expected by the intensity of stimulus and the analgesic state of the patient. The NoL index was found to be superior in comparison to all other physiological parameters and indices tested in this study (HR, PPGA, NIBP and SPI) and currently in use within the OR to monitor patients under GA, or referenced in the literature as nociception related. Furthermore, the NoL index algorithm, based on artificial intelligence learning, coupled with nonlinear integration of several physiological parameters and their time derivatives, proved to be better adapted for representing the analgesic and nociceptive states of patients under general anesthesia than any other monitor currently used.

# Sub article: Poster #039: Intrapulmonary Percussive Ventilation Superimposed on Pressure-regulated Volume-controlled Ventilation as a Rescue Therapy for Acute Respiratory Failure

**DOI:** 10.5041/RMMJ.10184

**Published:** 2015-01-29

**Authors:** Yaron Bar-Lavie, George Dahoud, Ami Mayo

**Affiliations:** 1 Division of Critical Care Medicine, Rambam Health Care Campus, Haifa, Israel

## Abstract

**Introduction:**

Intrapulmonary Percussive Ventilation (IPV) was introduced to Rambam Health Care Campus in September 2010. It was used as an adjunct to conventional ventilation in patients with severe respiratory failure where standard measures failed to improve their status.

**Materials and Methods:**

In an 18-bed medical-surgical Intensive Care Unit (ICU), IPV was superimposed on Pressure Regulated Volume Controlled Ventilation (PRVC) as a rescue therapy. All patients had severe Acute Respiratory Distress (ARDS) with refractory hypoxemia andor extreme hypercapnia, and failed maximal therapeutic efforts. These included muscle paralysis, Inverse-Ratio ventilation, Nitric Oxide inhalation, and prone position. Both IPV and PRVC were delivered in parallel to the airway via a special connector. The resulting pressure waveform was combined of slow squares representing PRVC with super-imposed rapid short rises representing IPV. The Fraction of Inspired Oxygen (FiO_2_) was set to maintain arterial saturation above 90%. Weaning from IPV was possible when respiratory parameters allowed returning to PRVC ventilation alone.

**Results:**

Over 2.5 years, 81 patients, 51 (63%) males, were placed on IPV. Nitric oxide inhalation was given to 75 (93%) patients; prone position was performed in 28 (35%). Median values (range): Age 50 (14–80); APACHE-II score 28.2 (11–48); ICU days 20.5 (1–163); ventilator days 18 (1–163); IPV days 3 (1–33); admission to IPV interval 4 days (0–35); and pre-IPV lung compliance 26 (10–63). Tracheostomy was performed in 42(52%). SAPS-2 median predicted (all cause) mortality was 53%; survival to ICU discharge, 43 patients (46.9% mortality). Immediate pre-IPV data and best results within the first 24 hours of IPV were prospectively collected and retrospectively analyzed (Table 1).

**Conclusions:**

Combining IPV with PRVC improved oxygenation and CO_2_ removal in these extremely ill patients with severe, refractory ARDS. Hemodynamic status was improved, as witnessed by increased blood pressure and urine flow. In order to evaluate the effect of combined IPV-PRVC ventilation on the outcome of patients with acute respiratory failure, a prospective randomized study is needed.

# Sub article: Poster #054: The Prognostic Value of B-Natriretic Peptide (BNP) Levels in Patients with Sepsis

**DOI:** 10.5041/RMMJ.10184

**Published:** 2015-01-29

**Authors:** Johad Khoury, Majd Arow, Geula Klorin, Ami Neuberger, Doron Aronson, Zaher S. Azzam, Gidon Berger

**Affiliations:** 1 Internal Medicine “B”; 2 Heart Institute, Rambam Health Care Campus, Haifa, Israel; 3 The Ruth & Bruce Rappaport Faculty of Medicine; 4 Research Institute in Medical Sciences, Technion-Israel Institute of Technology, Haifa, Israel

## Abstract

**Background:**

Sepsis is a clinical syndrome that complicates severe infection. Systemic inflammatory response syndrome (SIRS) is an identical clinical syndrome that complicates a noninfectious insult (e.g., acute pancreatitis, pulmonary contusion). Current theories about the onset and progression of sepsis and SIRS focus on dysregulation of the inflammatory response, including uncontrolled release of proinflammatory mediators with subsequent multiple organ dysfunction syndrome (MODS), which is the cause of the high mortality associated with these syndromes.

B-type natriuretic peptide (BNP) is a neuro-hormone that has been isolated first in the porcine brain and later in human ventricular cardiomyocytes. Both BNP and N-terminal pro-BNP are used for the early diagnosis of heart failure (HF) in patients presenting to the emergency department with dyspnea. Recently, elevated BNP levels have been measured in patients with septic shock and have been attributed to myocardial dysfunction due to sepsis. Because BNP synthesis is also induced by endotoxin and inflammatory mediators, the mechanisms leading to elevated BNP levels in patients with sepsis remain unclear. Little is known concerning N-terminal pro-BNP levels in patients with critical illness, especially with sepsis.

**Patients and Methods:**

We prospectively studied 259 patients with sepsis in the absence of heart failure. The BNP levels were obtained for all patients. The relationship between BNP and clinical outcomes was tested using multivariable analysis models.

**Results:**

Eighty-two patients died during the 90-day follow-up (31.66%), 53 died in the current hospitalization (20.5%), and 80 patients were re-admitted (30.9%). By bivariable logistic regression analysis of demographic data, risk factors and laboratory tests, a statistically significant correlation was found between BNP mortality and morbidity. According to multivariable analysis models, there was a clear increase in mortality and morbidity in the population of patients who had elevated BNP values.

**Conclusion:**

We have shown in a population of hospitalized patients with SEPSIS that BNP is a strong independent predictor of greater morbidity and mortality.

# Sub article: Poster #011: Long-Term Hemodialysis Therapy in Neonates and Infants with End-Stage Renal Disease: Experience and Outcome

**DOI:** 10.5041/RMMJ.10184

**Published:** 2015-01-29

**Authors:** Shirley Pollack, Israel Eisenstein, Mahdi Tarabeih, Hadas Shasha-Lavski, Daniella Magen, Israel Zelikovic

**Affiliations:** 1 Division of Pediatric Nephrology, Meyer Children’s Hospital, Rambam Health Care Campus; 2 The Ruth & Bruce Rappaport Faculty of Medicine, Technion-Israel Institute of Technology, Haifa, Israel

## Abstract

**Introduction:**

Peritoneal dialysis (PD) is the preferred mode of renal replacement therapy (RRT) in infants with end stage renal disease (ESRD). Hemodialysis (HD) is seldom used in neonates and infants because of its major complications in the very young.

**Patients and Methods:**

We analyzed demographic, clinical, laboratory and imaging data on all infants younger than 12 months with ESRD who received HD therapy in our Pediatric Dialysis Unit between January 1997 and June 2013.

**Results:**

Eighteen infants (M:F 6/12; Arabs/Jews 14/4) with ESRD (median age, 94 days; median weight, 4.06 kg) received HD through a central venous catheter (CVC) for a total of 16,292 days. Seven (39%) were neonates (<1 month of age; group 1) and 11 (61%) were infants (1–12 months; group 2) who received HD for a cumulative 7141 and 9151 days, respectively. In 6 of the patients, the initial mode of RRT was PD for 1–3 months. Five (28%) of the patients had serious comorbidities. Thirty-eight CVCs were inserted (34 angiographically). There were five episodes of CVC infection—a rate of 0.3/1000 CVC days. Median catheter survival time was 320 days. Five (28%) of the infants underwent renal transplantation and 10-year graft survival was 80%. Seven (39%) of the patients died. Most infants had good oral intake and only 4 (22%) required gastric tube. Thirteen (72%) of the infants displayed normal growth. Eight (44%) of the patients (5 in group 1) had delayed psychomotor development.

**Conclusions:**

Long term HD in neonates and infants with ESRD is technically feasible, can be implemented without major complications, carries a very low rate of CVC infection and malfunction, and results in good nutrition, growth and relatively good survival. Future efforts should aim to improve neurodevelopmental outcome and lower mortality rate in these infants.

# Sub article: Poster #015: Acute Hemodialysis therapy in Neonates with Inborn Errors of Metabolism

**DOI:** 10.5041/RMMJ.10184

**Published:** 2015-01-29

**Authors:** Israel Eisenstein, Mahdi Tarabeih, Shirley Pollack, Michael Halberthal, Ellie Hershman, Ori Attias, Amir Hadash, Hanna Mandel, Gad Bar-Joseph, Daniella Magen, Israel Zelikovic

**Affiliations:** 1 Division of Pediatric Nephrology, Meyer Children’s Hospital, Rambam Health Care Campus, Haifa, Israel;; 2 The Ruth & Bruce Rappaport Faculty of Medicine, Technion-Israel Institute of Technology; Haifa, Israel;; 3 Pediatric Intensive Care Unit, Meyer Children’s Hospital, Rambam Health Care Campus, Haifa, Israel; 4 Pediatric Metabolic Unit, Meyer Children’s Hospital, Rambam Health Care Campus, Haifa, Israel

## Abstract

**Introduction:**

Inborn errors of metabolism (IEOM) such as urea cycle defects (UCD) and organic acidemias (OA) can cause rapid, devastating and irreversible damage to the neonatal brain if not promptly treated. The most efficient therapy for IEOM-induced life-threatening metabolic crisis is hemodialysis (HD), which rapidly removes accumulated toxic compounds from the circulation. Data on the use of HD in IEOM, in particular in the neonatal period, is scarce.

**Objectives:**

This study summarizes our experience with acute HD in neonates with IEOM-induced metabolic/clinical crisis and describes the outcome of these neonates.

**Patients and Methods:**

We analyzed the demographic, clinical, and biochemical data of all neonates with IEOM who were admitted to our Pediatric Intensive Care Unit between January 2004 and September 2013, and presenting with a metabolic crisis necessitating HD therapy.

**Results:**

Sixteen neonates (M:F 8/8) with IEOM (6, UCD; 6, maple syrup urine disease (MSUD); 2, mitochondrial cytopathies (MC), and 2, other OA) had uneventful deliveries and were admitted because of metabolic/neurological deterioration starting at 48h–14 days of age. Median age and weight of the infants at initiation of HD was 6.7 days and 2944 g, respectively. Hemodialysis was performed through double-lumen, 6.5-French (Gambro, Lund, Sweden) acute catheter inserted blindly or under sonographic guidance into the internal jugular vein; AK200 (Gambro) dialysis machine and tubing, and FXpaed (Fresenius, Bad Homburg, Germany) filters were used. A total of 34 dialysis sessions (3 hours each; at least two sessions per patient) were performed. The first HD session markedly decreased (91.5%) mean ammonia level in UCD and MC patients from 1071 to 86 μmol/L (nl: 53–90), and markedly decreased (88%) mean leucine level in MSUD patients from 2381 to 284 μmol/L (nl: 00–240). The second dialysis session decreased the rebounded mean ammonia and leucine levels from 433 μmol/L and 1064 μmol/L to 69 μmol/L and 136 μmol/L, respectively. The HD procedure was free of complications, resulted in marked clinical improvement in 11 patients, and enabled the initiation of the appropriate dietary/pharmacological therapy in all patients. Thirteen (81.2%) of the infants survived, and three (18.7%) succumbed to their metabolic disease in the neonatal period.

**Conclusions:**

Hemodialysis in neonates with IEOM is safe, very efficient, and life-saving when performed by skilled personnel and in the appropriate setting.

# Sub article: Poster #005: ADHD in the Aging Population

**DOI:** 10.5041/RMMJ.10184

**Published:** 2015-01-29

**Authors:** Tali Fisher, Noga Adler, Gili Cochavi-Peretz, Yonit Tavor, Rachel Ben-Hayun, Judith Aharon-Peretz

**Affiliations:** Cognitive Neurology Unit, Rambam Health Care Campus, Haifa, Israel

## Abstract

**Introduction:**

During midlife and aging, subjective reports of cognitive problems become common. Conditions such as age associated cognitive impairment, mild cognitive impairment and dementia, increase in prevalence and are the diagnoses most often decided on. Background medical conditions and risk factors are often regarded as contributing to cognitive decline. The contribution of prior undiagnosed attention deficit hyperactivity disorder (ADHD) is seldom taken into account. The aim of the current study is to examine whether childhood or adult ADHD should be considered relevant in the differential diagnosis of cognitive complaints during midlife and aging.

**Patients and Methods:**

Neuropsychological evaluation consisted of the Conners’ Adult ADHD Rating Scale-SL, Beck Depression Inventory, Sleep Quality Questionnaire and the following cognitive tests: logical memory subscale (LM-WMS), California Verbal Learning Test (short form), CANTAB (PAL, IED), and the Test of Variables Attention (T.O.V.A.). The study was conducted at the Cognitive Neurology Clinic, at Rambam Health Care Campus and granted the approval of the local IRB (Helsinki committee).

Participants included 36 people, aged 50–70, diagnosed with probable ADHD (pADHD) and a control group of 29. The pADHD group included 12 individuals self-referred due to complaints of cognitive decline or memory impairment, previously undiagnosed with ADHD (ADHD-A) but with lifelong symptomatology of ADHD and fulfilling ADHD criteria, and 24 individuals who are the parents of diagnosed ADHD children and reporting ADHD symptoms (ADHD-B), but without complaints regarding recent cognitive decline.

**Results:**

The ADHD-A patients were impaired on attention parameters while memory and executive functions were intact. The ADHD-B patients did not present measurable attention or other neuropsychological deficits as compared to the control group. Neither group fulfilled criteria for mild cognitive impairment or dementia.

**Conclusions:**

Attention deficit hyperactivity disorder should be considered as a new/additional entity in the differential diagnosis of subjective cognitive complaints among middle-aged and older persons. Recognition of the specific cognitive and behavioral profiles of patients with ADHD should contribute to the ability to reach optimal differentiation from pre-dementia conditions in order to tailor appropriate therapies. The pathophysiology and future trajectory of the emerging ADHD symptomatology in older patients fulfilling lifelong ADHD symptomatology remains to be clarified. When examining older adults, ADHD should be considered as a differential diagnosis.

# Sub article: Poster #014: The Impact of Intertwined Peripheral and Brain Molecular Changes in Rat’s Behavioral Diversity: The Role of Branched Chain Amino Acid and Tryptophan Metabolism

**DOI:** 10.5041/RMMJ.10184

**Published:** 2015-01-29

**Authors:** Eyal Asor, Shiri Stempler, Avi Avital, Ehud Klein, Eytan Ruppin, Dorit Ben-Shachar

**Affiliations:** 1 Psychobiology Laboratory, Department of Psychiatry, Rambam Health Care Campus, Haifa, Israel;; 2 The Ruth & Bruce Rappaport Faculty of Medicine, Technion-Israel Institute of Technology, Haifa, Israel;; 3 The Sackler School of Medicine, Tel Aviv University, Tel Aviv, Israel; 4 Behavioral Neuroscience Lab, The Emek Medical Center, Afula, Israel

## Abstract

**Introduction:**

It is currently accepted that complex behavior and mental disorders result from a combination of biological susceptibility and exposure to environmental stimuli. Previously we showed that transient early in life interference with the expression of multiple genes by mithramycin followed by exposure to chronic stress, led to a “daring” and novelty seeking behavior in rats. In this study we used these rats to explore the molecular changes that may contribute to the behavioral alteration.

**Materials and Methods:**

Monoamines and amino acids levels were analyzed by HPLC in brain and serum. The RNA was extracted from the right prefrontal cortex for whole genome cDNA array analysis. Various *in silico* analyses were performed by Genome Studio Software (Illumina Inc., San Diego, CA), Ingenuity Pathways Analysis (IPA) and metabolic pathway analysis (iMAT and GSMMs). Candidate genes were validated by qRTPCR and Western blotting.

**Results:**

Data from the *in silico* analyses pointed at brain branched chain amino acids (BCAA) in mithramycin treated rats exposed to chronic stress (MTR+Stress). An HPLC analysis of brain amino acids showed no change in BCAA levels, yet a significant decrease was observed in tryptophan, whose brain levels highly depend on those of BCAA. Indeed, mRNA and protein levels of the BCAA and tryptophan transporter, large neutral amino acid transporter (LAT1) were significantly reduced. The latter was associated with reduced serum tryptophan/BCAA ratio. No change was observed in serotonin or its metabolite 5-HIAA as well as in kynurenine levels, checkpoints of two metabolic pathways of brain tryptophan. Finally, the increase in serum insulin, which plays a role in the regulation of serum BCAA levels following stress, was nulled in the MTR+Stress group. Significant high correlations were found between anxiety levels and the tryptophan levels as well as between T-maze errors and LAT1 mRNA levels.

**Conclusion:**

This study suggests that gene and environmental factors modulate both peripheral and central physiological processes, which converge and reciprocally interact to induce specific behavioral patterns Peripheral intervention can serve as a simple and novel add-on treatment for behavioral abnormalities.

# Sub article: Poster #016: Schizophrenia-derived Induced Pluripotent Stem Cells as a Platform to Study Apoptosis

**DOI:** 10.5041/RMMJ.10184

**Published:** 2015-01-29

**Authors:** Odile Robicsek, Ehud Klein, Dorit Ben-Shachar

**Affiliations:** 1 Psychobiology Laboratory, Department of Psychiatry, Rambam Health Care Campus; and the Ruth & Bruce Rappaport Faculty of Medicine; 2 The Ruth & Bruce Rappaport Research Institute, Technion-Israel Institute of Technology, Haifa Israel.

## Abstract

**Introduction:**

Dysregulation of apoptosis has been increasingly studied as a potential causative mechanism in schizophrenia; however, the data are still controversial. Although multiple pathways can induce apoptosis, the mitochondrial pathway has been most frequently implicated in CNS apoptosis. The apoptotic prototypical interaction occurs in the mitochondrial membrane between pro-apoptotic Bax and anti-apoptotic Bcl-2, finally leading to DNA fragmentation (the hallmark of active apoptosis). Previous studies found an impaired mitochondrial function in schizophrenia. We hypothesize that transfer of isolated active normal mitochondria (IAN-MIT) into patient cells may improve their impaired apoptosis.

**Materials and Methods:**

An attractive model for studying apoptosis in schizophrenia is to reprogram somatic cells into induced pluripotent stem cells (iPSCs) and to study their differentiation into neurons. We analyzed spontaneous and H_2_O_2_-induced Bcl-2/Bax ratio and DNA fragmentation levels in patient-derived iPSCs and their differentiated glutamatergic neurons from two healthy controls and two patients with schizophrenia. We first studied the apoptosis parameters. We then tried to correct the impaired apoptosis by transferring IAN-MIT into the cells.

**Results:**

In iPSCs, the spontaneous Bcl-2/Bax ratio was significantly higher in patients as compared to the controls (P<0.001). However, patient-iPSCs and controls showed similar sensitivity to induced apoptosis by H_2_O_2_, as the Bcl-2/Bax ratio was reduced by 67.42±7.36% in both groups. Spontaneous DNA fragmentation as observed by the TUNEL reaction was about four-fold lower (P<0.006) in patient-iPSCs than in control cells, while their sensitivity to H_2_O_2_ was higher by six-fold. The spontaneous Bcl-2/Bax ratio was higher (30% P<0.029) in patient differentiated neurons as compared with controls, similar to our findings in iPSCs. However, patient neurons showed lower sensitivity to induction of apoptosis by H_2_O_2_, as Bcl-2/Bax ratio was reduced by 93± 4.36% in control cells, whereas only by 56±1.98% in patient neurons. Spontaneous DNA fragmentation was about five-fold lower (P<0.021) in patient differentiated neurons than in control cells. Despite the similar sensitivity of control and patient neurons to H_2_O_2_, DNA fragmentation in the presence of H_2_O_2_ was significantly lower in patient neurons (P<0.009). The transfer of IAN-MIT had no significant effect on spontaneous or H_2_O_2_-induced Bcl-2/BAX ratio and DNA fragmentation in patient neurons.

**Conclusions:**

Our results point to an impaired apoptotic process in schizophrenia patient-derived cells, which further supports the impaired neurodevelopment hypothesis in schizophrenia.

# Sub article: Poster #017: Accuracy of Admission Diagnoses by Neurology Department Physicians: Comparison with Discharge Diagnoses

**DOI:** 10.5041/RMMJ.10184

**Published:** 2015-01-29

**Authors:** Tayir Alon, Moshe Herskovitz

**Affiliations:** 1 Department of Neurology, Rambam Health Care Campus, Haifa, Israel

## Abstract

**Introduction:**

A neurology department patient population is composed partially of patients with what should be a ‘clear cut’ diagnosis, receiving treatment and/or going through procedures aimed at secondary prevention, and of patients who are admitted with a complaint or one or more symptoms requiring diagnostic procedures and the appropriate treatment. We evaluated the initial diagnostic accuracy and the diagnostic yield in a 29-bed neurology department at an academic tertiary hospital.

**Materials and Methods:**

All patients admitted and discharged at the neurology department during the month of August 2014 were included; excluded were patients hospitalized in the EEG Video Telemetry Unit. The admission diagnoses were compared with the discharge diagnoses for agreement.

**Results:**

In all, 121 patients (62 males, 59 females, average age 57.3 years) were admitted and discharged during the study. A total of 124 consecutive admission and discharge documents were analyzed (three patients were admitted twice). The overall degree of agreement between initial and discharge diagnoses was 61.2%. Of the 48 changed diagnoses 7 (14.5%) of the cases were due to incorrect classification of the primary diagnosis at admission or discharge (for example three cases were coded as ‘headache’ instead of ‘meningitis’ on admission, although diagnosis of meningitis was made in the emergency department), and 36 (75%) were attributed to diagnostic work performed during hospitalization (a 48-year-old man suffering from convulsions was found to have a symptomatic cavernoma; a 79-year-old man admitted for diplopia was diagnosed with myasthenia gravis, etc.). For 5 (10.4%) of the cases, there was no diagnosis noted in the admission documents. Average duration of hospital stay for all study patients was 4 days.

**Discussion:**

A correct admission diagnosis of neurologic patients is crucial as it determines initial treatment and subsequent patient management. Nevertheless, the main role of many of neurology department hospitalizations is evaluation of symptoms and syndromes using specific specialized clinical skills, laboratory tests, and imaging, to arrive at the correct diagnosis. Emergency department assessment enabled a correct diagnosis in most cases. However, many cases were impossible to resolve without further investigation. Study of this patient series showed that despite the high patient turn-over and relatively short in-house stay, neurology department hospitalizations have a high diagnostic yield.

# Sub article: Poster #038: Early Connectivity Decline in High-order Cognitive Networks*

**DOI:** 10.5041/RMMJ.10184

**Published:** 2015-01-29

**Authors:** Tali Siman-Tov, Noam Bosak, Elliot Sprecher, Ayelet Eran, Judith Aharon-Peretz, Itamar Kahn

**Affiliations:** 1 Cognitive Neurology Institute, Rambam Health Care Campus, Haifa, Israel;; 2 Department of Neuroscience, The Ruth & Bruce Rappaport Faculty of Medicine and Research Institute, Technion-Israel Institute of Technology, Haifa, Israel;; 3 Laboratory of Clinical Neurophysiology, The Ruth & Bruce Rappaport Faculty of Medicine, Technion-Israel Institute of Technology, Haifa, Israel and Department of Neurology, Rambam Health Care Campus, Haifa, Israel; 4 Department of Diagnostic Imaging, Rambam Health Care Campus, Haifa, Israel

## Abstract

**Introduction:**

Aging has long been suggested to be accompanied by cognitive decline, even in the absence of dementia or other neurological insults. Decreases in gray and white matter volume and integrity parameters have been postulated to underlie this decline. More recently, late life cognitive impairment has also been linked to decreased functional connectivity within large-scale brain networks, particularly the default mode network (DMN). However, the exact time course of connectivity reduction along the adult lifespan and network-specific differences have not been addressed.

**Patients and Methods:**

We studied resting-state functional connectivity MRI (rs-fcMRI) data of 845 healthy subjects aged 21 to 83 years. Functional and anatomical data were obtained from the online dataset of the International Neuroimaging Data-sharing Initiative. Seed-based analysis was applied to analyze four high-order cognitive networks (DMN, salience network, dorsal attention network and fronto-parietal control network), and three primary sensory and motor networks. Node pair correlation matrices were computed for three age groups: young (Y, 21–40 yrs), middle-aged (M, 41–60 yrs) and old (O, 61–83 yrs). Difference matrices were calculated for the Y vs. M and M vs. O groups. Additionally, averaged *z(r)* values of node pair correlations within each network for each participant were analyzed by a mixed model repeated measures ANCOVA.

**Results:**

Lifetime decline of functional connectivity in high-order cognitive networks was nonlinear. It was characterized by an early reduction (reaching its nadir at the fifth to sixth decades), followed by a plateau. In contrast, a motor network showed increased connectivity in middle adulthood and a subsequent decline. Moreover, alterations in internetwork interactions were noticeable primarily in the transition between young and middle adulthood.

**Conclusions:**

These results confirm the hypothesis that brain aging involves adaptive reorganization of large-scale brain networks. Moreover, it stresses that most alterations, principally those related to high-order cognitive networks, occur relatively early in the adult lifespan.

# Sub article: Poster #050: Spinal Cord Stimulation Attenuates Temporal Summation in Patients with Neuropathic Pain

**DOI:** 10.5041/RMMJ.10184

**Published:** 2015-01-29

**Authors:** Elon Eisenberg, Yulia Burshtein, Erica Suzan, Roi Treister, Joshua Aviram

**Affiliations:** 1 The Ruth & Bruce Rappaport Faculty of Medicine, Technion-Israel Institute of Technology, Haifa, Israel;; 2 Pain Medicine Institute, Rambam Health Care Campus, Haifa, Israel;; 3 Department of Anesthesiology, Hillel Yaffe Medical Center, Hadera, Israel;; 4 Department of Neurology, Massachusetts General Hospital & Harvard Medical School, Boston, MA, USA; 5 Faculty of Social Welfare and Health Sciences, University of Haifa, Haifa, Israel

## Abstract

**Introduction:**

Spinal cord stimulation (SCS) is often use in the treatment of neuropathic pain, although the precise mechanisms by which it produces analgesia are not fully understood. Evidence from animal studies has shown that electrical stimulation at the dorsal columns attenuated the ‘wind up’ phenomenon in dorsal horn neurons in nerve-injured rats. The present study was aimed to test the effect of SCS on temporal summation (TS), the human clinical correlate of the ‘wind up’ phenomenon, in patients with radicular leg pain.

**Methods:**

Eighteen patients with SCS implants were tested both 30 minutes following SCS activation (‘ON’) and 2 hours after turning it off (‘OFF’), in a random order. Temporal summation was evaluated in the most painful site in the affected leg and in a corresponding area in the contra lateral leg, by applying tonic painful heat stimulus (46.5°; 120 seconds) and simultaneous recording of the perceived heat pain intensity. Patients were also requested to report their clinical pain intensity (0–100 numerical pain scale) during SCS ‘ON’ and ‘OFF’. Wilcoxon Signed Ranks test was used in the comparisons between SCS ‘ON’ and ‘OFF’.

**Results:**

The SCS activation significantly attenuated clinical pain intensity (from 66±18 to 27±31, p<0.001). In the non-painful leg SCS activation failed to produce an effect on TS (24±20 vs. 21±24 in SCS ‘OFF’ and ‘ON’, respectively; p=0.277). In contrast, a significant decrease in the magnitude of TS in the affected leg was observed in response to SCS activation (from 32±33 to 19±24; p=0.017).

**Conclusions:**

These results suggest that attenuation of TS, which likely represents suppression of hyperexcitability in spinal cord neurons, is a possible mechanism underlying SCS analgesia in patients with neuropathic pain.

# Sub article: Poster #051: Correlative Effect of Remifentanil on Neuropathic Pain and on Temporal Summation Points to Spinal Mechanism of Opioid Analgesia

**DOI:** 10.5041/RMMJ.10184

**Published:** 2015-01-29

**Authors:** Erica Suzan, Ayelet Midbari, May Haddad, Dorit Pud, Elon Eisenberg

**Affiliations:** 1 The Ruth & Bruce Rappaport Faculty of Medicine, Technion-Israel Institute of Technology, Haifa, Israel;; 2 Pain Medicine Institute, Rambam Health Care Campus, Haifa, Israel; 3 Faculty of Social Welfare and Health Sciences, University of Haifa, Haifa, Israel

## Abstract

**Introduction:**

Opioid analgesia is mediated by spinal and supra-spinal mechanisms. At the spinal level opioids attenuate the electrophysiological ‘wind-up’ phenomenon. The aims of the present prospective study were to test the effects of the short-acting μ-agonist remifentanil on temporal summation (TS), the clinical correlate of ‘wind up’ in patients with neuropathic pain (NP) and to correlate it with remifentanil’s analgesic effect on the clinical pain.

**Methods:**

Following obtaining a written informed consent fourteen patients (5F/9M) with chronic NP (sciatica), received an infusion of placebo (normal saline) followed by an infusion of remifentanil (target plasma concentration of 2 ng/ml) for a duration of 40 minutes each, in a single-blinded fashion. Temporal summation, spontaneous clinical pain intensity (0–100 numerical pain scale, NPS), and evoked pain (straight leg raise=SLR) were assessed 20 minutes after the initiation of each infusion. The TS was evaluated by a paradigm of tonic heat stimulation (46.5°) for 120 seconds and pain intensity was simultaneously recorded on a Computerized Visual Analogue Scale (COVAS, 0–100).

**Results:**

The magnitude of TS during saline and remifentanil infusions significantly differed from each other (33.4±29 and 13.1±23.39, respectively; Wilcoxon Signed Ranks test, p=0.033). During remifentanil infusion, compared to placebo, clinical pain was lower (47.5±23.9 and 23.9±18.7, p=0.002) whereas SLR was significantly higher (34o±20.3 and 25.8o±15.6, p=0.004). Significant correlation was found between the difference in TS (remifentanul vs. placebo) and the difference in the intensity of the clinical pain under these two conditions (r=0.538, p=0.039). A similar trend towards a significant correlation between the difference in TS and the difference in SLR was also observed (r= −0.474, p=0.074).

**Conclusions:**

These results suggest that remifentanil reduces TS and chronic NP as evident by improvement in both patients’ reports of pain intensity and in SLR. In addition, the correlation between the reduction in TS and the reduction in NP intensity suggests that opioids exhibit their analgesic effect via reducing excitability at the spinal cord level.

# Sub article: Poster #052: Objective Assessment of Spinal Cord Stimulation Effectiveness on Chronic Radicular Pain

**DOI:** 10.5041/RMMJ.10184

**Published:** 2015-01-29

**Authors:** Nir Ben-Israel, Erica Suzan, Yariv Amos, Galit Zuckerman, Noam Racheli, Mark Kliger, Elon Eisenberg

**Affiliations:** 1 Medasense Biometrics Ltd, Ramat Yishai, Israel;; 2 Pain Medicine Institute, Rambam Health Care Campus, Haifa, Israel; 3 The Ruth & Bruce Rappaort Faculty of Medicine, Technion-Israel Institute of Technology, Haifa, Israel

## Abstract

**Background:**

We recently showed that a combination of multiple autonomic parameters accurately differentiates between experimental pain intensities in healthy subjects. However, this technology has not been tested so far in patients with chronic pain. Spinal cord stimulation (SCS) is widely used in the treatment of pain conditions when conservative or less invasive techniques are ineffective. The present study was aimed to test the ability of the combination of autonomic parameters to objectively detect effectiveness of SCS treatment.

**Methods:**

Thirty-three patients with chronic radicular (neuropathic) pain in one lower extremity and permanent SCS participated in the study. Patients were requested to rate the intensity of their pain on numerical pain scale (NPS, 0–100) twice, at a random order: 30 minutes after turning the SCS on and 2 hours after turning it off. For the purpose of this study, a difference of 15 NPS points or greater between the two ratings (stimulator “on” and “off”) was regarded as an “effective SCS.”

Photoplethysmogram (PPG) and skin conductance (SC) were recorded twice, along with the subjective pain ratings, each time for 120 sec, using the PMD-100™ (Medasense Biometrics, Ramat Yishai, Israel). The following autonomic parameters and their derivatives were extracted: PPG wave amplitude, PPG wave amplitude variation, pulse rate (PR) interval, PR variability and SC fluctuations. The parameters were combined using a non-linear classifier. The accuracy, sensitivity, specificity, positive and negative predicted values (PPV and NPV) of each parameter alone and their combination were calculated. Paired t-test was used for statistical analyses.

**Results:**

Effective SCS was found in 18 patients and ineffective in 15 (ΔNPS: 40±10 vs. 8.5±0, respectively; p<0.05). The combination of the parameters outperformed each of the parameters alone in the detection of the SCS effectiveness, with regards to all classification performance criteria (accuracy of the combination was 85% and that of each parameter ranged from 52% to 70%; sensitivity 94% vs 53–82%; specificity 75% vs 38–73%; PPV 76% vs 64–76%; NPV 92% vs 46–79%). The study is still ongoing.

**Conclusions:**

These findings suggest that autonomic-based multi-parameter assessment may be used for objective measurement of SCS effectiveness.

# Sub article: Poster #073: Pro-nociceptive Pattern of Pain Modulation Characterizes Patients with Painful Diabetic Neuropathy

**DOI:** 10.5041/RMMJ.10184

**Published:** 2015-01-29

**Authors:** Yelena Granovsky, Hadas Nahman-Averbuch, Mogher Khamaisi, David Yarnitsky, Michal Granot

**Affiliations:** 1 The Laboratory of Clinical Neurophysiology, Technion Faculty of Medicine, Haifa, Israel;; 2 Department of Neurology, Rambam Health Care Campus, Haifa, Israel;; 3 Endocrinology, Diabetes and Metabolism Institute and Department of Internal Medicine, Rambam Health Care Campus, Haifa, Israel and The Ruth & Bruce Rappaport Faculty of Medicine, Technion-Israel Institute of Technology, Haifa, Israel; 4 Faculty of Health and Welfare Sciences, Haifa University, Haifa, Israel

## Abstract

**Introduction:**

Pro-nociceptive pain modulation profile (PMP) characterized by less-efficient descending inhibition and enhanced ascending facilitation was observed in various chronic pain syndromes including neuropathic pain. However, the characteristics of a PMP in painful diabetic neuropathy (PDN) have not yet been fully explored. This study aimed to: (1) illuminate the response pattern to experimentally-induced pain for ‘static’ and ‘dynamic’ psychophysical measures in PDN, (2) to investigate the relationships between PDN-related clinical parameters (pain intensity and neuropathy duration) and pain modulation characteristics, and (3) to evaluate the role of anxiety and pain catastrophizing levels on the PMP and clinical pain parameters.

**Materials and Methods:**

Heat pain threshold, pain scores to pinprick mechanical stimulation, mechanical temporal summation, temporal changes along tonic painful heat stimulation and conditioned pain modulation (CPM) were tested at non-affected regions, and compared between 28 PDN patients and 33 healthy controls.

**Results:**

The PDN patients demonstrated less-efficient CPM responses (3.9±17.7 vs. −7.6±18.1; P=0.019), deficient adaptation to the tonic heat stimulation (51.3±14.3 to 50.9±24.1 vs. 60.3±14.1 to 47.3±25.0, numerical pain scale (NPS); P=0.013) and higher scores for the mechanical pinprick pain (25.6±24.6 vs. 13.5±12.6, NPS; P=0.017). Patients also had higher levels of pain catastrophizing (26.9±10.7 vs. 13.8±9.7; p<0.001).

**Conclusions:**

The pro-nociceptive PMP was most evident for patients with higher intensity of ongoing pain and shorter duration of neuropathy. These findings indicate the pro-nociceptive PMP of PDN patients, demonstrating the precise alteration of mechanisms involved in their pain processing. Categorizing neuropathic pain patients based on pain modulation characteristics and neuropathy duration may be considered in determining treatment approach.

# Sub article: Poster #083: Therapeutic Efficacy of Continuous Theta-Burst Magnetic Stimulation in Major Depression: A Double-Blind Sham-Controlled Study

**DOI:** 10.5041/RMMJ.10184

**Published:** 2015-01-29

**Authors:** Andrei Chistyakov, Bella Kreinin, Boris Kaplan, Adel Khatib, Nawaf Darawsheh, Menashe Zaaroor, Ehud Klein

**Affiliations:** 1 Department of Neurosurgery, Rambam Health Care Campus, Haifa, Israel;; 2 The Ruth & Bruce Rappaport Faculty of Medicine, Technion-ITT, Haifa, Israel; 3 Department of Psychiatry, Rambam Health Care Campus, Haifa, Israel

## Abstract

**Background:**

Theta-burst transcranial magnetic stimulation (TBS) has been shown to induce potent and long lasting effects on cortical excitability. In a previous open study, we demonstrated safety, tolerability, and antidepressant properties of continuous TBS (cTBS) in major depression (MD). The present study was aimed to evaluate the therapeutic efficacy of cTBS in depressed patients using a double-blind, sham-controlled design

**Materials and Methods:**

Twenty-nine patients with MD were randomized to receive either active cTBS to the right dorsolateral prefrontal cortex (n=15) or sham cTBS (n=14) for 10 consecutive work days. After the tenth session, patients who received sham TBS were crossed over to active cTBS which consisted of 10 daily sessions. Patients who received active cTBS continued with the same treatment protocol for an additional 10 treatments. Each treatment session consisted of 3600 stimuli at an intensity of 100% of the active motor threshold. Severity of depression was assessed weekly.

**Results:**

Overall, there was no significant difference in the degree of clinical improvement between active and sham cTBS groups. However, in patients whose medication status remained unchanged before the trial (n=8) and in those who were medication-free (n=3), active cTBS resulted in a significantly greater reduction of Hamilton depression scores as compared to sham cTBS.

**Conclusions:**

Our results suggest that the antidepressant effect of cTBS is modest, yet it might be beneficial to patients nonresponsive to ongoing pharmacological treatment. A direct comparison between cTBS and conventional rTMS protocols is warranted.

# Sub article: Poster #002: Effect of Very Early Parent Training on Feeding Interaction and Infant Eating Habits at 12 Months

**DOI:** 10.5041/RMMJ.10184

**Published:** 2015-01-29

**Authors:** Inbal Balog, Oded Pshetatzki, Iris Elad, Yael Latzer, Geila S. Rozen

**Affiliations:** 1 Faculty of Social Welfare and Health Sciences, University of Haifa, Haifa, Israel;; 2 Department of Clinical Nutrition, Rambam Health Care Campus, Haifa, Israel; 3 Eating Disorders Clinic, Rambam Health Care Campus, Haifa, Israel

## Abstract

**Introduction:**

This study examined whether professional training of inexperienced mothers of 4-month-old infants improves feeding relationship and the eating habits of infants at 12 months.

**Patients and Methods:**

First-time mothers were recruited 4–6 months after delivery for a training program (intervention group). Training was in small workshop groups, once a week for the duration of a month. Internet-based support was continued until infants reached 12 months by an experienced pediatric dietitian and social worker. The control group received customary support given at municipal mother-child health clinics. Mealtime interactions were evaluated at 12 months using blinded viewing of videotaped feeding interaction in the home setting by the Chatoor Feeding Scale. Infants eating habits were self-reported using questionnaires.

**Results:**

The training program was completed by 86 mothers. Controls of 42 mother-infant dyads were recruited. Mothers were 30 (+2.6) years old, with 16 (+2.2) years of education, all normative according to personality questionnaires, and without clinical eating disorders. Mealtime interactions according to the Chatoor Feeding Scale revealed statistically significant differences in favor of the intervention group (IG) for four of the five dimensions: dyadic conflict (IG 4.69 vs. 8.38 control), talk and distraction (3.75 vs. 4.9), struggle for control (2.3 vs. 4.88), and maternal non-contingency (1.61 vs. 2.75). This indicated more positive mother-infant interactions during mealtime, and a better response to the infant’s cues. The eating habits self-report indicated IG infants consumed less snacks at 12 months (1.08 vs. 1.38), less sweets (1.44 vs 2.07), and fewer sweet-drinks (1.03 vs 1.27). The intervention group mothers reported lower frequency of vomiting or spiting-up during mealtime (1.89 vs 2.41). The intervention group infants were also less distracted during mealtime (2.83 vs 3.29).

**Conclusion:**

This research evaluated very early maternal training as a means of developing a positive feeding relationship at infancy. Results suggest that parental guidance can support the establishing of a more positive feeding interaction of 12-month-old infants. This may contribute to better and healthier eating habits with preserved internal eating cues based on hunger and satiety. We postulate this may prevent future eating disorders and obesity. Long-term follow-up and variation of groups is necessary to further examine these affects and optimize training programs to better accommodate specific target populations.

# Sub article: Poster #003: The Relationship between Maternal Pathology Associated with Eating Disorders and Observed Feeding Interaction with Their One-year-old Infant

**DOI:** 10.5041/RMMJ.10184

**Published:** 2015-01-29

**Authors:** Inbal Balog, Oded Pshetatzki, Iris Elad, Yael Latzer, Geila S. Rozen

**Affiliations:** 1 Faculty of Social Welfare and Health Sciences, University of Haifa, Haifa, Israel;; 2 Department of Clinical Nutrition, Rambam Health Care Campus, Haifa, Israel; 3 Eating Disorders Clinic, Rambam Health Care Campus, Haifa, Israel

## Abstract

**Introduction:**

Feeding interaction is an essential part of parenting, especially in infancy. Errors in the early stages of feeding such as maternal control of feeding practice by restriction or pressure to eat may cause problems in feeding and eating patterns for infants in the present and future. These feeding interaction errors often originate with a mother’s pathology associated with eating disorders.

The aim of this study was to examine the relationship between maternal pathology associated with eating disorders and the observed feeding interactions with their one-year-old infants. In addition, we examined whether mother-child interaction group counseling for mothers of 4-month-old infants affects this relationship.

**Patients and Methods:**

We performed a cross-sectional study comprised of 128 mothers aged 23–35, after their first birth, and living in Haifa and its surroundings. Eighty-six mothers underwent group counseling on feeding relationship when their infants were between the ages of 4–6 months. The rest of the mothers did not undergo counseling. The feeding relationship was evaluated by videotaping mothers and their 12–month-old infants during mealtime and then coded using the Chatoor questionnaire. In addition, mothers were asked to complete a questionnaire to examine their pathology associated with eating disorders (Eat 26, EDI).

**Results:**

We found an association between the mother’s pathology associated with eating disorders and the feeding interaction with their one year old infants in some of the measurements. The score on the distractions dimension was higher among mothers who reported having more eating psychopathology and with a higher tendency for drive for thinness. It was also found that mothers who reported having more eating psychopathology had a higher tendency for control during mealtime. Counseling mothers about feeding interaction did not affect this relationship.

**Conclusions:**

The tendency for control and use of distractions during mealtime are associated with a maternal pathology associated with eating disorders. This may be one of the mechanisms that contribute to the effect of the intergenerational transmission of eating pathology. Therefore, finding methods of treatment and proper feeding relationship counseling for mothers with pathology associated with eating disorders may reduce and possibly eliminate this risk.

# Sub article: Poster #012: Night Eating Syndrome among Patients with Binge Eating Disorder or Bulimia Nervosa: Dietary Patterns

**DOI:** 10.5041/RMMJ.10184

**Published:** 2015-01-29

**Authors:** Adi Elron Yutal, Orna Tzischinsky, Miri Givon, Sigal Alon, Michal Rozenstain-Hason, Nehama Zuckerman-Levin, Orna Kabakov, Yael Latzer

**Affiliations:** 1 Faculty of Social Welfare and Health Sciences, University of Haifa, Haifa, Israel;; 2 Psychology Department, Emek Yezreel Academic College, Yezreel Valley, Israel; 3 Eating Disorders Clinic, Psychiatric Division, Rambam Health Care Campus, Haifa, Israel

## Abstract

**Introduction:**

Bulimia nervosa (BN) and Binge eating disorder (BED) are two eating disorders (EDs) which involve binge eating behavior as a primary symptom. Night eating syndrome (NES) is conceptualized as a delay in the circadian pattern of food intake, manifested by two core criteria: evening hyperphagia and/or nocturnal ingestions. It is not entirely clear whether NES among persons with BED or BN may represent a separate entity, or a variant of BED or BN, in which the binge episodes occurs mainly in the evening. Specifically, it is unclear whether the frequency of binge episodes, the amount of calories consumed, and the macronutrient composition are similar between subjects with BED or BN with and without NES.

**Patients and Methods:**

This cross-sectional study included 56 women, aged 18–60, recruited from the outpatient ED clinic of Rambam Health Care Campus, Haifa, Israel. The participants completed food diaries, a demographic questionnaire, the Eating Disorder Examination-Questionnaire, and the Beck Depression Inventory. A linear regression model was fitted for each dependent variable. Potential confounders were included in each model. For each model in which the study group variable was the only significant variable, an independent samples t-test was performed.

**Results:**

Of 56 participants, 33 (58.9%) were diagnosed with BED or BN (BE), and 23 (41.1%) were diagnosed with BED or BN, in addition to NES (BE+NES). The BE+NES group reported a significantly higher frequency of binge days (3.34±2.34 vs. 1.85±2.06, p=0.016) and binge episodes (4.55±4.39 vs. 2.23±2.71, p=0.024) during the week, as well as higher energy consumption (2,643.5±1,374.9 vs. 1,868.6±851.3, p=0.014) and fat consumption (36.9%±4.3% vs. 32.2%±4.8% of daily intake, p=0.001) than the BE group.

**Conclusions:**

Our findings indicated that patients who have NES in addition to BED or BN have higher levels of eating pathology than patients who have BED or BN alone. In light of these results, we suggest that NES among patients with BED or BN may not represent just a variant of BED or BN, but rather a separate entity, which leads to a more severe disorder.

# Sub article: Poster #021: Lower Uterine Segment Thickness and Cervical Length may Indicate the Etiology of Preterm Deliveries in Twin Gestations

**DOI:** 10.5041/RMMJ.10184

**Published:** 2015-01-29

**Authors:** Yuval Ginsberg, Israel Goldstein, Nizar Khatib, Naama Fargo, Zeev Weiner

**Affiliations:** Department of Obstetrics and Gynecology, Rambam Health Care Campus, Haifa, Israel

## Abstract

**Objectives:**

This study evaluated the lower uterine segment (LUS) and cervical length (CL), measured at 16–25 weeks, as predictors for preterm birth in twin pregnancies.

**Patients and Methods:**

Both LUS thickness and CL were measured in women with twin gestation between 16–25 weeks of gestation. Demographic data, time, and cause of delivery were documented. Etiologies for PTD were divided into premature contractions, premature rupture of membranes (PPROM), and iatrogenic (sever PET, abnormal fetal well-being, IUGR, etc). Statistical analysis was performed to correlate between CL and LUS thickness and the different etiologies of PTD.

**Results:**

Inclusion criteria were met by 243 women with twin gestation; 41% delivered at term (>37 weeks); 26% delivered before 35 weeks; and 8% delivered before 32 weeks of gestation. Overall, both CL and LUS thickness were significantly decreased in women with PTD (Figure 1). Focusing on the different etiologies of PTD revealed that women with PTD caused by premature contractions showed significantly shorter CL with almost no change in the LUS thickness. However, women with PTD caused by PPROM demonstrated almost no change in CL but a significant decrease in the LUS thickness (Figure 1). Women with PTD before 32 weeks of gestation demonstrate similar findings.

**Conclusions:**

The LUS thickness and CL measured during the 2nd trimester of pregnancy are both predictors of PTD in twin pregnancies. Moreover, they may predict different etiologies of PTD which may indicate different preventive treatment modalities.

# Sub article: Poster #022: Risk Factors for an Abnormal Oral Glucose Tolarance Test Six to Twelve Weeks after Delivery in Women with Gestational Diabetes

**DOI:** 10.5041/RMMJ.10184

**Published:** 2015-01-29

**Authors:** Yuval Ginsberg, Naama Fargo, Zeev Weiner

**Affiliations:** Department of Obstetrics and Gynecology, Rambam Health Care Campus, Haifa, Israel

## Abstract

**Objectives:**

The objective of this study was to identify risk factors for obtaining an abnormal oral glucose tolarance test (75 gr OGTT) in women with gestational diabetes mellitus (GDM).

**Materials and Methods:**

Included in this study were 538 patients with GDM that were treated in the HMO high risk clinics (MACCABI). For all participants relevant data was gathered including: maternal age, parity, family history of diabetes, gestational week at diagnosis, spontaneous or ART pregnancy, BMI score at the beginning of pregnancy, at time of diagnosis of GDM, and at the end of pregnancy, fasting plasma glucose levels at the beginning of the pregnancy, the 100 gr OGTT results, GDM treatment (insulin, oral medication, or diet therapy), gestational age and mode of delivery, fetal weight, and complications. The OGTT (75 gr. Glucose) was perfomed 6–12 weeks after delivery. Statistical analysis was performed in order to compare between an abnormal OGTT 75 gr. (over 140 mg/dl) 6–12 weeks post-delivery and the variables presented.

**Results:**

Thirty patients (5.6%) had abnormal 75 gr OGTT results; 508 (94.4%) were normal. Only the fasting plasma glucose level either at the beginning of pregnancy (91.7gr±9.2 vs 95.7gr±8.7; p=0.023) or during the 100 gr OGTT (105.2gr± 40.85 vs 127.5gr±51.3; p=0.002) correlated with abnormal 75 gr OGTT performed 6–12 weeks after delivery. Neither maternal age, BMI, weight gain during pregnancy, maternal or fetal outcome, mode of treatment (Insulin or diet), or other variables assessed in this study correlated with the 75 gr OGTT.

**Conclusion:**

We suggest that the recommendation for early post-delivery OGTT should be limited to gestational diabetic patients with additional risk factors.

# Sub article: Poster #066: Effects of Maternal Hyperoxygenation on Fetal Pulmonary Circulation and on Blood Flow Patterns in the Middle Cerebral and the Umbilical Arteries in Normal and High-risk Pregnancies

**DOI:** 10.5041/RMMJ.10184

**Published:** 2015-01-29

**Authors:** Nizar Khatib, Nadir Ganem, Ron Beloosesky, Israel Goldstein, Israel Thaler, Zeev Weiner

**Affiliations:** Department of Obstetrics & Gynecology. Rambam Medical Center Haifa, Israel

## Abstract

**Introduction:**

The aim of the present study was to compare fetal blood flow patterns in the cerebral and pulmonary arteries in women with low- and high-risk pregnancies, prior to and following maternal hyperoxygenation.

**Materials and Methods:**

Ten pregnant women with normal- and ten women with high-risk pregnancy participated in the study. All had a singleton gestation with mean gestational age of 35.2±3.5 and 35.6±3.4 weeks, respectively. Doppler blood flow velocity waveforms were obtained from the middle cerebral artery (MCA), the main pulmonary artery, and the proximal right pulmonary artery. Pulsatility indices (PI) were calculated for the pulmonary arteries and MCA. Following baseline measurements, each woman received 70% humidified oxygen for ten minutes. Doppler measurements were then repeated.

**Results:**

Maternal hyperoxygenation caused a significant and similar decrease in PI values in the main and the right pulmonary artery in both groups. The PI value in the MCA did not change significantly following maternal hyperoxygenation in the low risk group (pre-O_2_ 1.64±0.31 and post-O_2_ 1.60±0.41). However, in the high risk group it increased significantly from 1.44±0.27 to 1.73±0.51, respectively; p<0.02.

**Conclusion:**

This study shows that in both normal and high risk pregnancies hyperoxygenation abolishes the relative vasoconstriction in fetal pulmonary vessels. While no significant change was observed in the MCA in the low risk group, the relative brain sparing phenomena that was observed in the high risk group was completely abolished following O_2_ in the high risk group. These findings may be useful in monitoring high-risk pregnancies and suggest that maternal hyperoxygenation may be beneficial to the fetus in high-risk pregnancies.

# Sub article: Poster #067: Programming of Offspring Adaptive Immune Response Following Maternal Inflammation

**DOI:** 10.5041/RMMJ.10184

**Published:** 2015-01-29

**Authors:** Nizar Khatib, Zeev Weiner, Yuval Gensberg, Ron Beloosesky

**Affiliations:** 1 Department of Obstetrics and Gynecology, Rambam Health Care Campus, Haifa, Israel

## Abstract

**Introduction:**

Prenatal infection with gram negative bacteria is common during pregnancy. We have shown previously that acute maternal LPS exposure at E18 programs significant decrease the offspring innate immune response. The adaptive immune response is composed of B and T lymphocytes. T helper (CD4+, CD8-) and T Cytotoxic (CD8+, CD4-) compose the majority of T cells, and are essential for adequate immune responses. While T helper cells play an important role in establishing and maximizing the capabilities of the immune system, T Cytotoxic destroy virally infected cells and tumor cells. We sought to determine whether the adaptive immune response is programmed by maternal inflammation.

**Materials and Methods:**

Pregnant Sprague Dawley rats (n=4) at 18 days gestation received intraperitoneal (i.p.) injections of LPS (500 μg/kg) or saline (control). Male and female pups were delivered spontaneously (e21) and allowed to mature until postnatal day 24 (p24). CD+4 and CD+8 lymphocytes, granulocytes, and macrophages expressing Toll-like receptor 4 (TLR4) were characterized in the blood, spleen, and thymus of offspring pups by flow cytometry.

**Results:**

Pups of LPS-treated dams had significantly lower blood T Cytotoxic lymphocytes than pups of saline treated dams, though the same number of T helper lymphocytes. Pups of LPS dams had significantly higher counts of blood CD4+ TLR4+ and CD8+ TLR4+ lymphocytes, TLR4+ granulocytes, and a trend towards higher TLR4+ macrophages. The same pattern was demonstrated in the spleen and thymus.

**Conclusion:**

The decrease in the T Cytotoxic lymphocyte in the Offspring of LPS exposed dams may increase their ability to cope with viral infection and potential tumor development. The increase in CD4+ TLR4 + lymphocytes may aid in anti-inflammatory responses and may explain the decrease in the pro-inflammatory cytokines previously demonstrated in these offspring following exposure to LPS. These results suggest that maternal inflammation during the antenatal period may induce long term sequelae in the offspring innate immune response which may predispose to adult disease.

# Sub article: Poster #068: Maternal Magnesium Sulphate (Mg) Neuroprotection May be Mediated via Placental ACTH and Fetal Brain Leukemia Inhibitory Factor Receptor (LIF-R) Modulation

**DOI:** 10.5041/RMMJ.10184

**Published:** 2015-01-29

**Authors:** Ron Beloosesky, Yuval Ginsberg, Nizar Khatib, Zeev Weiner

**Affiliations:** Department of Obstetrics and Gynecology, Rambam Health Care Campus, Haifa, Israel

## Abstract

**Introduction:**

Maternal magnesium sulphate (Mg) may protect the preterm fetus from inflammation-associated brain injury. Leukemia inhibitory factor (LIF) is a pleiotropic cytokine essential for central nervous system development. Maternal LIF stimulates placental ACTH secretion, which subsequently induces fetal LIF secretion from nRBCs, ultimately promoting fetal brain development. Thus, transmission of maternal LIF signal to the fetus is mediated by placental ACTH. We hypothesized that maternal infection/inflammation may influence placental ACTH/fetal brain LIF signal transduction pathway and these effects may be prevented with Mg.

**Materials and Methods:**

Pregnant rats at day 18 (n=6) received intraperitoneal injections of lipopolysaccharide (LPS) or saline (SAL) at time 0 and randomized to receive subcutaneous Mg or SAL for 2 hours prior to and following LPS or SAL injections. At 4 hours after LPS administration, dams were sacrificed and fetal brain and placenta were collected for determination of ACTH, LIF, and LIF-R protein levels in each group (LPS/SAL, LPS/Mg, SAL/SAL, SAL/Mg). Relative band density was determined after normalization to ß-actin levels.

**Results:**

Administration of LPS/SAL significantly increased ACTH, LIF and LIF-R protein levels in the placenta compared to SAL/SAL group (0.89± 0.04 vs 0.12±0.015 u, 1.0±0.17 vs 0.12+0.02 u, 0.46±0.07 vs 0.1±0.08 u, respectively, p<0.05). Magnesium sulphate treatment to LPS dams (LPS/Mg) significantly decreased placental ACTH and LIF-R compared to LPS dams (0.27± 0.07 vs 0.89±0.04 u, 0.25±0.05 vs 0.46±0.07 u, respectively). In the fetal brain, LPS/SAL significantly increased LIF levels compared to SAL/SAL but did not change LIF-R levels. Magnesium sulphate treatment to LPS dams decreased significantly LIF-R levels compared to LPS dams.

**Conclusion:**

Lipopolysaccharide-simulated infection during pregnancy may attenuate fetal brain development and induce brain injury through increased placental ACTH and brain LIF. Magnesium sulphate may protect the fetus by suppressing both placental secretion of ACTH and fetal brain LIF-R levels.

# Sub article: Poster #069: Therapeutic N-acetyl-cysteine following Initiation of Maternal Inflammation Attenuates Long-term Offspring Cerebral Injury as Evidenced by MRI

**DOI:** 10.5041/RMMJ.10184

**Published:** 2015-01-29

**Authors:** Ron Beloosesky, Hila Sharabi, Nizar Khatib, Yuval Ginsberg, Michael Ross, Mordechai Hallak, Zeev Weiner

**Affiliations:** 1 Division of Obstetrics and Gynecology, Rambam Health Care Campus, Haifa, Israel;; 2 Obstetrics, Gynecology and Reproductive Science Department, Hillel Yafe Medical Center, Hadera, Israel; 3 The Department of Obstetrics and Gynecology, Harbor-UCLA Medical Center, Torrance, CA, USA.

## Abstract

**Introduction:**

Maternal infection/inflammation may induce fetal inflammatory responses that have been associated with long-term offspring cerebral injury. We previously demonstrated that prophylactic N-acetyl-cysteine (NAC), administered prior to and following maternal lipopolysaccharide (LPS), reduced the extent of cerebral injury measured by MRI after birth. This study examines the effect of therapeutic NAC on neonatal brain injury following maternal LPS-induced inflammation, as measured by MRI.

**Materials and Methods:**

Pregnant Sprague Dawley dams (n=5) at 18 days gestation received an intraperitoneal injection of either LPS (500 μg/kg) or saline at time 0. Animals were randomized to receive an intravenous injection (tail vein) of NAC (300 mg/kg) or saline (time +30 minutes). Pups were delivered spontaneously and allowed to mature until postnatal day-25. The male offspring (5–8 per group) were examined by MRI and analyzed using voxel-based analysis. The apparent diffusion coeffient (ADC) was determined using diffusion tensor imaging to assess white and gray matter injury. The extent of injury was analyzed in three dimensions: The medial coefficient (MD), axial diffusivity (AD) and radial diffusivity (RD).

**Results:**

The offspring of LPS-treated dams exhibited significantly increased MD, AD and RD levels in the white and gray matter (e.g., thalamus, amygdale, visual cortex, corpus callosum, and hypothalamus), consistent with cerebral injury. In contrast, the offspring of NAC-treated LPS dams demonstrated, in most regions reduced MD, AD and RD levels, similar to the saline group.

**Conclusion:**

Maternal NAC treatment following maternal inflammation significantly reduced evidence of neonatal brain injury. These studies suggest that maternal NAC therapy may be effective in human deliveries associated with maternal/fetal inflammation such as preterm rupture of the membranes and chorioamnionitis.

# Sub article: Poster #070: New Model, Based on Cervical Length, Predicts Successful Vaginal Birth after Cesarean Section

**DOI:** 10.5041/RMMJ.10184

**Published:** 2015-01-29

**Authors:** Nizar Khatib, Matanes Emad, Ron Beloosesky, Zeev Weiner, Yuval Ginsberg, Nadir Ganem

**Affiliations:** Department of Obstetrics and Gynecology, Rambam Health Care Campus, Haifa, Israel

## Abstract

**Introduction:**

The rate of repeat cesarean delivery (CD) is above 90%. This increase is mainly attributed to a decrease in vaginal births after CD. In the last years several models have been proposed to predict the vaginal birth after cesarean section (VBAC) success rate, none of which included cervical length (CL). The aim of this study is to examine the accuracy of a cervical length model to predict VBAC success.

**Materials and Methods:**

Pregnant women with singleton pregnancies, after 30 week of gestation interested in trail of labor after cesarean section (TOLAC) in our institution from April 2013 through March 2014 were included. Patients underwent transvaginal measurement of CL by one physician. Women in labor or with uterine contractions at presentation were excluded. The CL measurement was recorded and blinded from the attending physician. Clinical and demographic data were collected. After delivery logistic regression was used based on cervical length to predict successful VBAC.

**Results:**

After 30 weeks of gestation (79 patients >36 weeks), 91 pregnant women agreed to participate and were interested in TOLAC. The mean cervical length for women who had successful VBAC was 2.7cm±0.94 compared to 3.6 cm±1.05 for the women who failed VBAC and had repeat CD (p<0.0001). Multivariate logistic regression found that only a short CL and previous vaginal delivery were associated with an increased rate for VBAC. For each reduction of 10 mm in the cervical length, the odds ratio for vaginal delivery was 3.3 (p<0.0001; Table 1). Sub-analysis found that CL was predictive only when measured after 36 weeks of gestation.

**Conclusion:**

Cervical length measurement after 36 weeks of gestation has high predictive accuracy for VBAC. Further larger studies, should set up a combined model that includes cervical length and other parameters, to predict successful VBAC.

# Sub article: Poster #026: Exosomes Secreted by Macrophages Facilitate Chemotherapy Resistance in Pancreatic Ductal Adenocarcinoma*

**DOI:** 10.5041/RMMJ.10184

**Published:** 2015-01-29

**Authors:** Yoav Binenbaum, Jacob T. Cohen, Ziv Gil

**Affiliations:** Applied Cancer Science Laboratory, Clinical Research Institute at Rambam Health Care Campus and the Ruth & Bruch Rappaport Faculty of Medicine, Technion-Israel Institute of Technology, Haifa, Israel

## Abstract

**Introduction:**

Pancreatic ductal adenocarcinoma (PDAC) ranks fourth among cancer-related deaths in the US. Gemcitabine (Gemzar. Eli Lilly) is the drug of choice for PDAC patients, despite its modest efficacy. The disappointing effectiveness of the drug is attributed largely to Gemcitabine resistance, which develops within weeks of initiation of therapy. Our group has recently demonstrated that tumor-associated macrophages cause gemcitabine resistance by inducing the level of cytidine deaminase (CDA), the enzyme that metabolizes Gemcitabine to its inactive form in the PDAC cell. The ligand-receptor interaction that mediated this phenomenon, however, could not be identified.

**Methods:**

Exosomes were collected from conditioned media of IL-4 induced tumor associated macrophages. Exosomes were applied to PDAC cells in the presence of gemcitabine, and cell proliferation was assessed by XTT. Resistance was assessed following exosome treatment with RNase and DNase. Exosomes stained with PKH-67, a membrane incorporating dye, were applied on PDAC cells; internalization to PDAC cells was assessed by confocal microscopy. Exosome RNA content was screened for potential miR targets by qRT-PCR. Finally, Gemcitabine resistance in the presence of exosomes was assessed in PDAC cells treated with antagomiR (miR inhibitor), which acted as a ‘miR-trap.’

**Results:**

Exosome treatment led to Gemcitabine resistance in a dose dependent manner, inducing 28% resistance in comparison to non-treated cells (p<0.05). RNase treatment on exosomes, but not DNase, reversed Gemcitabine resistance (p<0.05, p=0.3 respectively). Stained exosomes were found to be internalized to the PDAC cell, 89% of which were found in the cytoplasm and 11% in the PDAC cell nucleus. Tumor-associated macrophage-derived exosomal RNA content was found to be enriched in specific miRs, as compared to the exosomal content of resting macrophages (p<0.05). AntagomiRs abolished exosome mediated gemcitabine resistance in PDAC cells (p<0.05).

**Discussion:**

Our results demonstrate that gemcitabine resistance is mediated, at least partially, by macrophage-derived exosomes. This mechanism for inter-cellular communication is supported by our finding that Exosomes that are secreted by macrophages are internalized to cancer cells. This inter-cellular communication is mediated by shuttling of miRNA’s between the macrophages and tumor cells.

**Conclusion:**

AntagomiRs augment chemotherapy efficacy and might be a novel treatment for PDAC patients.

# Sub article: Poster #027: Marrow-derived Macrophages Mediate Invasion of Pancreatic Adenocarcinoma by RET Activation

**DOI:** 10.5041/RMMJ.10184

**Published:** 2015-01-29

**Authors:** Moran Amit, Shorook Na’ara, Yoav Binenbaum, Ziv Gil

**Affiliations:** 1 Applied Cancer Research Laboratory and the; 2 Department of Otolaryngology Head and Neck Surgery, Rambam Health Care Campus, Haifa, Israel

## Abstract

**Introduction:**

Perineural invasion of cancer cells (CPNI) is found in most patients with pancreatic adenocarcinomas (PDAs). Although the presence of M2 macrophages in the perineural environment has been associated with shortened survival rates in PDA patients, the mechanism underlying this phenomenon has not been elucidated. It has been suggested that secretion of the RET ligand glial-derived neurotrophic factor (GDNF) by M2 macrophages causes cancerous RET-expressing cells to migrate along affected nerves. This concept, however, has not been demonstrated *in vivo*. This study was aimed at understanding the contribution of macrophages to perineural invasion for *in vivo* models of PDA, and to characterize the role of the GDNF-RET axis in this unique microenvironmental niche.

**Methods:**

Immunohistochemical analysis of PDA specimens of a novel Pdx-1-Cre/KrasG12D /p53R172H (KPC) mouse model, revealed that CD163+ (M2) endoneurial macrophages (EMΦs) predominate the endoneural space of invaded nerves. In a bone marrow transplant (BMT) from *CX3CR*^GFP/+^ mice, a 23-fold increase in the recruitment of GFP+ macrophages to the perineural space was witnessed as compared to GFP-cells, indicating recruitment of bone marrow-derived macrophages to the perineural niche, rather than resident tissue macrophages. A BMT from *CCR2^−/−^* mice lacking macrophage recruitment capacity resulted in a 6-fold decrease of the invasion severity score, and a 2.5-fold decrease in perineural invasion index. Analysis by fluorescence microscopy indicated that EMΦs expressed higher levels of GDNF compared to EMΦs in premalignant lesion and normal controls. In order to differentiate between the contributions of GDNF secreted by nerves to the invasion presses and its secretion from recruited M2 macrophages, we performed a BMT from GDNF*^+/−^* mice. The BMT led to a 1.5-fold improvement in both the invasion severity score and the perineural invasion index. Similarly, mice treated with PP1, a RET receptor inhibitor, caused a 1.5-fold decrease in the invasion severity score, and a 1.3-fold decrease in the perineural invasion index. Finally, xenografts of PDAC cells, in which RET was silenced using siRNA, resulted in a 3.5-fold decrease in the invasion severity score and a 3.2-fold decrease in the perineural invasion index.

**Discussion:**

Taken together, our results suggest a paracrine response between bone marrow-derived tumor-associated EMΦs and PDA cells. This loop probably orchestrates the formation of cancer nerve invasion. Further studies using novel transgenic animal models aimed at specifying the specific molecular signals that induce disease invasion and proliferation in the perineural micro-environment are needed.

# Sub article: Poster #034: The Possible Role of Different Biomarkers in the Prognosis of Head and Neck Sarcoma

**DOI:** 10.5041/RMMJ.10184

**Published:** 2015-01-29

**Authors:** Christine Namuz, Adi Rachmiel, Imad Abu El Naaj, Dekel Shilo, Ofer Ben Izhak, Sharon Akrish, Yoav Leiser

**Affiliations:** 1 Department of Oral and Maxillofacial Surgery; 2 The Oral Caner Research Laboratory, Oral and Maxillo-facial Department; 3 Department of Pathology, Rambam Health Care Campus, Haifa, Israel; 4 The Ruth & Bruce Rappaport Faculty of Medicine, Technion-Israel Institute of Technology, Haifa, Israel

## Abstract

**Introduction:**

Head and neck tumors of mesenchymal origin (mainly sarcomas), have a very poor prognosis and most of the patients are subjected to surgical morbidity and eventually mortality. The complex anatomy of the head and neck usually prevents a surgical resection with clear margins. As a result an increased risk of recurrences is documented.

**Aim:**

In this study we aimed to examine the expression levels of three genes known to be associated with biological development and tumor progression, and to correlate the staining levels of the protein expression with our head and neck sarcoma patient outcomes.

**Materials and Methods:**

A retrospective immunohistochemical study was performed for heparanase, tuftelin, and FANCF in 20 head and neck sarcomas and 20 mesenchymal origin controls.

**Results:**

Heparanase and Tuftelin were found to be overexpressed in head and neck sarcomas compared with control. Almost 70% of the sarcoma tissues were stained positive for heparanase compared to only 35% of the control tissues. For Tuftelin, 50% of the sarcoma tissues were stained positive while only 10.5% of the control samples. No significant difference was detected between the sarcoma and control tissues in the FANCF staining levels; 15% of the sarcoma tissues were stained positive to FANCF and 10.5% of the control tissues. When we tried to correlate the staining levels to patient outcomes (5-year survival rate), Tuftelin overexpression was correlated with poor prognosis of our patients. Heparanase staining levels exhibited no significant benefit in the survival of sarcoma patients. All of the FANCF-positive patients did not survive more than one year following their diagnosis while (61%) of sarcoma FANCF-negative survived 5 years after diagnosis.

**Conclusions:**

Currently, The Oral Cancer Research Laboratory at Rambam is investigating the mechanism of expression, possible roles, and the quantitative PCR analysis of these genes, which may help in the future understanding of head and neck sarcomas. The preliminary results presented in this work highlight the poor prognosis of sarcoma and the importance of large scale studies to help establish molecular markers for these aggressive tumors of head and neck mesenchymal origin.

# Sub article: Poster #037: The Possible Role of Viral Infection in the Etiology of Oral Cancer

**DOI:** 10.5041/RMMJ.10184

**Published:** 2015-01-29

**Authors:** Yoav Leiser, Yasmin Gantous, Dekel Shilo, Ofer Ben Izhak, Sharon Akrish, Imad Abu El Naaj

**Affiliations:** 1 Department of Oral and Maxillofacial Surgery; 2 The Oral Caner Research Laboratory, Oral and Maxillofacial Department, Rambam Health Care Campus, Haifa, Israel;; 3 The Ruth & Bruce Rappaport Faculty of Medicine, Technion-Israel Institute of Technology, Haifa, Israel; 4 Department of Pathology, Rambam Health Care Campus, Haifa, Israel

## Abstract

**Introduction:**

Human papillomavirus (HPV) has been confirmed as the primary etiological factor that transforms cervical epithelia into cancer. The presence of HPV in oral cancers suggests that HPV may play a similar role in transforming the oral epithelia. A high degree of variability in the prevalence of HPV in oral cancers has been found, however, raising questions regarding its role in the transformation and development of oral cancer. The aim of the present study is to analyze, using tissue array technology and PCR (Polymerase Chain Reaction), the possible influence of viral infection on the carcinogenesis of oral carcinomas.

**Materials and Methods:**

The study included 59 biopsies of oral neoplasms. Immunostaining of formalin fixed, paraffin-embedded 5 microns tissue array sections was performed. Slides were incubated with the primary antibody, LMP1 for EBV, pp65 for CMV, and P16 for HPV. Slides were scored as 0=no staining, 1=weak staining, and 2=strong staining, and the specific intracellular localization was recorded. Fresh tissue DNA was extracted (TRIZOL). The PCR Primers for specific viral DNA were fabricated. The total DNA was evaluated using PCR.

**Results:**

The ages of the patients in the experimental group ranged from 38 to 92 years (mean 63 years). A predominant number of the OSCC (Oral Squamous Cell Carcinoma) patients had reported tobacco use (47%). A much smaller portion reported consumption of alcohol (6%). Immunoreactivity for CMV and EBV was not significant, while HPV antibody expression was found in 32% of the cases. In 40% of the OSCC patients, HPV DNA was detected using PCR, While in control normal tissues of the same patient HPV genotype was expressed only in two tissue specimens (9%), one of which had positive margins for OSCC.

**Conclusions:**

A correlation between HPV and OSCC was found in this study. In 40% of the tissues, HPV DNA was expressed exclusively in the tumor tissues and not in the same patients’ normal contra lateral epithelium. These finding support specific HPV infection and not generalized oral epithelium infection as the possible etiological factor for HPV induced oral cancer. We also found that cancer patients who were HPV-positive were significantly younger and had better prognoses than patients who were negative for HPV infection.

# Sub article: Poster #043: The Future Treatment Using Cold Atmospheric Plasma for Cancer

**DOI:** 10.5041/RMMJ.10184

**Published:** 2015-01-29

**Authors:** Jacob T. Cohen, Yoav Binenbaum, Ziv Gil

**Affiliations:** 1 Department of Otolaryngology Head and Neck Surgery, Rambam Health Care Campus, Haifa, Israel; 2 Applied Cancer Research Laboratory, The Clinical Research Institute, Rambam Health Care Campus, Haifa, Israel and The Ruth & Bruce Rappaport Faculty of Medicine, Technion-Israel Institute of Technology, Haifa, Israel

## Abstract

**Introduction:**

Non-Thermal Plasma (NTP), with ion temperature close to room temperature has tremendous applications in biomedical engineering, and can potentially offer a surgical option for cancer cell elimination without influencing the whole tissue. The aim of our study was to evaluate the safety of NTP treatment, and its effect on melanoma cells both *in vitro* and *in vivo*.

**Materials and Methods:**

Our team has developed a prototype of NTP delivery device that can be used both *in vitro* and *in vivo*. *In vitro*, we evaluated NTP effect on the proliferation of melanoma B16-F0 cells by XTT assay. *In vivo*, we treated cutaneous melanoma tumors that were induced in nude-mice. The IVIS Kinetic Optical System (Caliper LifeSciences, Hopkinton, MA, USA) was used in-order to quantify epidermal penetration of melanoma cells. All samples were examined pathologically following H&E staining.

**Results:**

(Figure 1): The NTP treatment lead to a dose-response reduction in melanoma cell proliferation rate *in vitro*. The NTP treatment of 60 and 120 seconds resulted in 68.7% and 83.5% proliferation reduction, respectively (p<0.01). *In vivo*, melanoma flank tumors treated by NTP (n=3) had 45.26% volume reduction as compared to non-treated controls, but the effect was not significant. However, melanoma penetration to the epidermal layer was reduced by 48.2% in the treatment group (120,868 pixels as compared to 62608 pixels, p<0.05). The skin unaffected by melanoma was not harmed by NTP treatment.

**Conclusion:**

We explored the effect of our novel NTP delivery devise on cancer progression, both *in vitro* and *in vivo*. It is envisioned that adaptation of NTP technology for clinical use may provide a new modality for the treatment of solid tumors.

# Sub article: Poster #044: An Orthotopic Mouse Model of Laryngeal Squamous Cell Carcinoma

**DOI:** 10.5041/RMMJ.10184

**Published:** 2015-01-29

**Authors:** Jacob T. Cohen, Ziv Gil, Yoav Binenbaum, Shorook Na’ara, Moran Amit

**Affiliations:** 1 Department of Otolaryngology Head and Neck Surgery, Rambam Health Care Campus, Haifa, Israel; 2 Applied Cancer Research Laboratory, the Clinical Research Institute, Rambam Health Care Campus, and The Ruth & Bruce Rappaport Faculty of Medicine, Technion-Israel Institute of Technology, Haifa, Israel

## Abstract

**Objectives:**

This study aimed at creating a reliable and reproducible orthotopic mouse model of laryngeal malignancy that recapitulates its biologic behavior, local invasiveness, and spread as seen in patients.

**Methods:**

The first mouse Laryngeal Speculum design was hand-crafted of a silicon tube with different shapes attached to an adult ear speculum. The selected prototype was printed using a three dimensional printer. Using this speculum it was possible to view mouse larynxes. Using direct laryngoscopy, human squamous cell carcinoma line FaDu (ATCC® HTB-43™) was implanted in the larynxes (supraglottis and glottis) of nu/nu mice (n=31) (Figure 1). Animals were monitored with direct laryngoscopy and ultrasound for tumor growth and survival. Specimens of larynxes, neck lymphatics, livers, and lungs were removed for histopathologic evaluation to assess tumor extension, thyroid cartilage invasion, and nodal spread.

**Results:**

We performed 31 successful direct mouse laryngoscopies. Supraglottic and glottis tumor uptake was 100% and 25%, respectively. Median survival for the animals with supraglottic tumors was 35 days. Histopathologic evaluation revealed pre-epiglottic extension, paraglottic extension, thyroid cartilage invasion, and lymph node metastasis.

**Conclusions:**

We describe the first orthotopic model for laryngeal cancer. Our model faithfully recapitulates the phenotype and malignant behavior that reproduces its natural biologic behavior as seen in laryngeal cancer patients. This model offers an opportunity to identify and specifically target therapy for larynx squamous cell carcinoma.

# Sub article: Poster #047: The Involvement of Tuftelin Protein in Oral Cancer

**DOI:** 10.5041/RMMJ.10184

**Published:** 2015-01-29

**Authors:** Dekel Shilo, Dan Deutsch, Anat Blumenfeld, Adi Rachmiel, Yoav Leiser

**Affiliations:** 1 Department of Oral and Maxillofacial Surgery, Rambam Health Care Campus, Haifa, Israel;; 2 Dental Research Laboratory, Institute of Dental Sciences, Hebrew University, Hadassah, Faculty of Dental Medicine, Jerusalem, Israel; 3 The Ruth & Bruce Rappaport Faculty of Medicine, Technion-Israel Institute of Technology, Haifa, Israel

## Abstract

**Introduction:**

Tuftelin was originally identified in the enamel extracellular matrix and is expressed in the epithelial ameloblasts at an early stage of amelogenesis. Tuftelin cDNA sequences were detected in morula, embryonic stem cells, and different mesenchymal mineralizing and soft tissues, normal as well as cancerous. Recently we found that during mouse embryonic development, the dynamic spatio-temporal expression pattern of tuftelin seem to shift from the cytoplasm to the nucleus with developmental progression. Tuftelin mRNA and protein were increased during hypoxia (1% O_2_), through induction of HIF1α, in the neuronal PC12 cell model and in mesenchymal stem cells. Down-regulation of HIF1α mRNA in PC12 cells reduced hypoxia-induced tuftelin expression by 89%.

**Materials and Methods:**

Indirect immunohistochemiostry was performed using Histostain^®^-SP kit (Zymed^®^, San Francisco, CA, USA). Polyclonal anti tuftelin antibodies raised against tuftelin synthetic peptides were used. Total RNA was extracted using TRI-reagent. Reverse transcription was performed using high-capacity cDNA reverse transcription kit. Real-time quantitative PCR was performed using TaqMan^®^ Assays-On-Demand (Applied Biosystems^®^, Foster City, CA, USA).

**Results:**

Examination of human oral squamous cell carcinoma (SCC), ameloblastoma, and verrucous carcinoma, revealed strong staining for tuftelin protein in these types of tumors as compared to control tissues. In addition, tuftelin protein staining intensity in head and neck sarcomas was significantly higher in cancer samples as compared to normal, non-cancerous samples from the same individuals. Real-time PCR showed a 24-fold elevation of tuftelin mRNA expression in oral cancer tissues and 77-fold elevation in verrucous carcinoma, as compared to normal, non-cancerous samples from the same individuals.

**Conclusions:**

Our previous results on tuftelin involvement in hypoxia, and its nuclear localization during late embryonic development and post-natally, indicates that tuftelin is involved in the adaptive mechanisms, regulating protective processes, possibly by regulating gene expression. These results can explain tuftelin’s expression in SCC and sracomas of the head and neck. Tuftelin expression in these tumors can also be explained by preliminary results of our lab, in which tuftelin has been shown to be involved in cell proliferation. Tuftelin’s involvement in cancer progression needs to be further investigated. Tuftelin should also be investigated as a possible marker for SCC and sarcomas.

# Sub article: Poster #056: Polymorphous Low Grade Adenocarcinoma: Case Series and Review of Surgical Management

**DOI:** 10.5041/RMMJ.10184

**Published:** 2015-01-29

**Authors:** Amir Wolff, Imad Abu El-Naaj, Yoav Leiser

**Affiliations:** Department of Oral and Maxillofacial Surgery, Rambam Health Care Campus, Haifa, Israel; and the Ruth & Bruce Rappaport Faculty of Medicine, Technion-Israel Institute of Technology, Haifa, Israel

## Abstract

**Purpose:**

The aim of the present study was to review the published data regarding the management of polymorphous low-grade adenocarcinoma (PLGA) and present our experience in the treatment of patients diagnosed with PLGA.

**Materials and Methods:**

We performed a review of the published data of the treatment possibilities for PLGA, and report on a case series of four patients diagnosed with palatal PLGA.

**Results:**

The mean age at diagnosis was 61.75 years. All cases were localized on the hard palate. The male/female ratio was 1:3. One patient had an ulcerative painful lump. In correlation with the published data, most of our patients presented initially with a diagnosis that was not conclusive for the presence of PLGA. The most common initial diagnostic findings were adenoid cystic carcinoma and pleomorphic adenoma. The treatment of choice was surgical excision of the tumor with safe margins of 1.5 cm. One patient had a positive cervical lymph node metastasis and underwent an elective neck dissection followed by radiotherapy. Local recurrence was diagnosed in 1 patient 6 years after the surgical management. Most cases were reconstructed using an obturator, except for 1 patient who underwent reconstruction using the temporalis myofascial flap.

**Conclusions:**

PLGA is a slow-growing, distinct, uncommon neoplasm of the minor salivary glands. Because it is characterized by a diverse morphologic pattern that resembles adenoid cystic carcinoma or pleomorphic adenoma, the initial diagnosis is usually inconclusive. Until a final diagnosis has been made, we believe that the reconstruction possibilities should be mainly obturators to allow a good visualization of the surgical site. The use of microvascular free flaps or rotational flaps should be reserved to patients with clearer pathologic diagnosis.

# Sub article: Poster #007: The Probability of Invasive Fungal Infection among Children with Persistent Febrile Neutropenia and Respiratory Viral Infection

**DOI:** 10.5041/RMMJ.10184

**Published:** 2015-01-29

**Authors:** Avigail Rein, Nira Arad, Moran Amit, Ayelet Ben-Barak, Yael Shachor, Imad Kassis, Myriam Weyl Ben-Arush

**Affiliations:** 1 Pediatric Hematology – Oncology and Bone Marrow Transplantation, Rambam Health Care Campus, Haifa, Israel;; 2 Department of Otolaryngology, Head and Neck Surgery, Rambam Health Care Campus, Haifa Israel; 3 Pediatric Infectious Diseases Unit, Rambam Health Care Campus, Haifa, Israel

## Abstract

**Introduction:**

Febrile neutropenia (FN) is a common complication among children undergoing chemotherapy for hematologic malignancies. A microbial agent is identified in 15–30% of these episodes. Recently, several studies reported a high incidence (25%–60%) of respiratory viral infections (RVI) in children with FN. Implementation of RVI workup in children with FN was suggested, in order to minimize the overuse of antibiotics. However, data on the incidence of RVI in children with persistent FN (PFN) is meager. In this setting, invasive fungal infection (IFI) is a major concern, although most children who present with PFN rarely develop IFI. Yet, most international guidelines indicate empiric initiation of antifungal therapy after 4–7 days of FN.

Based on published data, we hypothesized that RVI is a significant cause of PFN in children with hematologic malignancies, and that IFI is the cause only in minority of cases. We further aimed to investigate whether detection of RVI in these children, would affect their risk of IFI.

**Materials and Methods:**

Charts of children with hematologic malignancies, presented to our department during 2007–2013 with PFN (>38°c, for >96 hours and ≤500ANC/μl) and documented PCR of RVI, were analyzed. Patients were considered IFI positive if they had ‘possible,’ ‘probable,’ or ‘proven’ infection according to revised EORTC criteria. Respiratory viral infectons were detected by PCR and included: RSV, Influenza, Parainfluenza, HMPV, Adenovirus, and HHV-6.

**Results:**

A total of 75 PFN episodes were evaluated. Of these, there were 31 RVI episodes (41.3%). There were 16 possible (21%), 2 probable (2.6%), and 2 proven IFI episodes. There were only 3 episodes of IFI and RVI co-infections. Multivariate analysis revealed that RVI was significantly associated with reduced risk for IFI – O.R of 7.47 (p<0.0082, 95% CI −1.6–50.4). Total days of neutropenia, and antibiotic therapy were independent risk-factors for IFI (p<0.0109; P< 0.0034 respectively).

**Conclusion:**

Children with PFN and RVI have a significantly lower risk for IFI. Further studies are needed in order to establish whether RVI-status can be implemented in the workup algorithm of PFN in children with hematologic malignancies.

# Sub article: Poster #028: Alteration in Tongue Muscle Co-activation during Sleep in Obstructive Sleep Apnea

**DOI:** 10.5041/RMMJ.10184

**Published:** 2015-01-29

**Authors:** Yaniv Dotan, Giora Pillar, Arie Oliven

**Affiliations:** 1 Pulmonary Division, Rambam Health Care Campus, Haifa, Israel;; 2 Sleep Laboratory, Rambam Health Care Campus, Haifa, Israel; 3 Internal Medicine, Bnai Zion Medical Center, Haifa, Israel

## Abstract

**Introduction:**

Obstructive sleep apnea (OSA) is characterized by pharyngeal obstructions during sleep. We have recently demonstrated that during sleep and mild anesthesia, upper airway collapse is not explained solely by sleep-induced decline in pharyngeal dilator muscle activation, as dilator muscles EMG activity often increases during obstructive apneas and hypopneas, but fails to improve airflow. The present study was undertaken to evaluate the hypothesis that failure of intense activation of tongue protrudors to prevent pharyngeal obstruction may be associated with sleep-induced changes in tongue muscle coordination.

**Materials and Methods:**

Esophageal pressure (Pes), airflow, and intramuscular EMG of the genioglossus (GG) and tongue retractors (TR) were recorded in 15 OSA patients. While awake, EMG’s of tongue protrusion and retraction were compared to biceps and triceps EMG flexion and extension. EMG/Pes was assessed while patients breathed through variable resistors, to estimate the magnitude of EMG required to prevent pharyngeal collapse. During sleep, EMG/Pes was evaluated during flow limitation. Pre-arousal peak inspiratory EMG (sleepEMG) was compared to peak EMG at the same Pes during wakefulness (awakeEMG). SleepEMG/awakeEMG >1 indicated a level of EMG expected to be sufficient to prevent pharyngeal collapse.

**Results:**

Patients were 47.7 ± 10.7 years old, with Apnea/Hypopnea Index of 61.8 ± 16.1/hr. During wakefulness, the pattern of antagonist tongue muscle activation differed considerably from that of the arm antagonist muscles, as both GG and TR were activated similarly during all tongue movements. During sleep, the levels of GG-EMG before arousal were significantly larger than those observed during wakefulness at equal Pes (125±69%, p<0.01). TR-EMG levels, however, were significantly lower than those recorded during wakefulness (52±21%, p<0.05).

**Conclusions:**

In OSA patients during sleep, augmentation of GG-EMG parallels respiratory efforts during obstructed breathing, but fails to improve airflow. This mechanical inefficiency is not explained by high inspiratory suction pressures, as GG-EMG may increase to levels by far higher than those required to prevent pharyngeal collapse during wakefulness. Unlike the similar pattern of activation of GG and TR during wakefulness, co-activation of TRs is deficient during sleep. This alteration in the pattern of tongue muscle activation may be involved in the pathogenesis of pharyngeal obstruction during sleep.

# Sub article: Poster #080: SM-art MEDicine: The Guiding Light to Right Medicine

**DOI:** 10.5041/RMMJ.10184

**Published:** 2015-01-29

**Authors:** Ronen Zalts, Yakir Moshe, Ali Yahia, Bella Smolin, Afif Yaccob, Nirit Grushko, Vera Belsky, Liz Ghazi, Matti Waterman, Gidon Berger

**Affiliations:** 1 Division of Internal Medicine, Rambam Health Care Campus, Haifa, Israel; 2 Intel Electronics, Ltd., Haifa, Israel.

## Abstract

**Background:**

SM-art MEDicine is a project of the Division of Internal Medicine at Rambam Health Care Campus. The project was started in the beginning of 2014 as the result of a Rambam-Intel collaboration program and was also inspired by the American Choosing Wisely® campaign. Healthcare worldwide has undergone many changes over the past years, mainly due to the introduction of new technologies, imaging modalities, blood tests, and procedures. This new reality presents a huge challenge to medical teams with regard to intelligently managing the medical diagnosis process, choosing the right diagnostic tests, and perhaps most important, to know when a supporting test is *not* necessary.

Our aim is to add order and logic to this process by making common medical practices within the Division more exact, focused, and clinically based. This supports a few goals such as minimizing waste of medical resources, reducing the number of patient days, reducing the workload of supporting staff, and providing safer patient care.

**Materials and Methods:**

The project is an educational campaign managed by senior doctors of the six internal medicine wards at Rambam Health Care Campus. This forum meets once every three weeks and discusses day to day practice topics (with the supporting literature), together with an external content expert and two Intel engineers. At the end of the meeting the team writes up a set of recommendations that are communicated to the doctors through various channels. The primary purpose of the recommendations is to refine, clarify and shed light on problematic areas in the overall work and management of internal medicine cases mainly pertaining to the uneducated use of hospital resources. The team also implements structural changes to the indicated process. Finally, the project impact is measured by quantifiable data from blood and imaging tests, where applicable.

**Results:**

A significant drops in various tests was observed after the recommendations were published throughout the departments and the structural changes were implemented. There was a 30% reduction (P<0.0001) in BNP tests, a 25% drop (P<0.0001) in Troponin tests, and and LDH testing was reduced by more than 50% (not yet statistically tested).

**Summary:**

The SM-art MEDicine campaign has led to a marked reduction in the administration of diagnostic tests by improving the management of medical investigations and more intelligent use of medical resources.

# Sub article: Poster #029: The Input of Measuring the Drug and Antibody Levels in the Management of Patients with Autoimmune Diseases, Treated with Monoclonal Anti-TNF Agents

**DOI:** 10.5041/RMMJ.10184

**Published:** 2015-01-29

**Authors:** Yolanda Braun-Moscovici, Amir Dagan, Kochava Toledano, Shomron Ben Horin, Doron Markovits, Amer Saffouri, Rema Beshara, Alexander Rozin, Menahem A. Nahir, Yehuda Chowers, Alexandra Balbir-Gurman

**Affiliations:** 1 B. Shine Rheumatology Unit, Rambam Health Care Campus, Haifa, Israel;; 2 The Ruth & Bruce Rappaport Faculty of Medicine, Technion-Israel Institute of Technology, Haifa, Israel;; 3 Department of Gastroenterology, Sheba Medical Center, Rambat Gan, Israel;; 4 Sackler School of Medicine,Tel-Aviv University, Tel Aviv, Israel; 5 Gastroenterology Institute, Rambam Health Care Campus, Haifa, Israel

## Abstract

**Introduction:**

A subset of patients does not react to anti-TNF agents or lose response. Increasing the dose has economic implications and switching to another biological may be suitable for rheumatoid arthritis (RA), but problematic for other autoimmune diseases in which the therapeutic “arsenal” is limited. This study was aimed at assessing the role of measuring the drug and antibody (ab) levels in the management of patients (pts) with autoimmune diseases, treated with monoclonal anti-TNF agents.

**Materials and Methods:**

Trough serum levels and antibodies (abs) to infliximab (INF) and adalimumab (ADA) were measured by ELISA in pts treated at the ambulatory day care of Rheumatology of Rambam Health Care Campus. Anti-TNF abs were identified by anti-human lambda chain ab. Treated pts were classified into responders and non-responders based on disease activity.

**Results:**

One hundred and thirty-one serum tests for INF and 48 for ADA were performed in 101 pts (mean age 50.2 and disease duration 9.9 years). Among responders 51 pts received INF and 16 pts received ADA. Among non-responders 34 pts had no response and 10 pts lost response. Levels of INF and ADA in responders were significantly higher than in non-responders; levels of anti-INFAb and anti-ADA-Ab in responders were significantly lower than in non-responders. High levels of anti-drug Abs predicted treatment discontinuation. In all non-responders with low INF/ADA levels and low anti-INF-Ab/anti-ADA-Ab the shortening of retreatment intervals lead to significant improvement (Table 1).

**Conclusions:**

Assessment of immunogenicity of anti-TNF monoclonal antibodies provided useful information for guiding the therapy in auto-immune diseases with suboptimal clinical response. Patients with low INF/ADA levels and low levels of corresponding Ab may benefit from increasing the drug dose or decreasing of retreatment intervals. In pts with negligible serum levels of INF/ADA and high levels of corresponding Ab the therapy should be switched to a different drug.

# Sub article: Poster #030: Upper Gastrointestinal Bleeding is Associated with Higher Mortality in Systemic Sclerosis

**DOI:** 10.5041/RMMJ.10184

**Published:** 2015-01-29

**Authors:** Yolanda Braun-Moscovici, Doron Markovits, Ian Gralnek, Kochava Toledano, Rema Beshara-Garzoz, Amir Dagan, Alexander Rozin, Yehuda Chowers, Alexandra Balbir-Gurman

**Affiliations:** 1 B. Shine Rheumatology Unit, Rambam Health Care Campus, Haifa, Israel;; 2 Gastrointestinal Institute, Rambam Health Care Campus, Haifa, Israel; 3 The Ruth & Bruce Rappaport Faculty of Medicine, Technion-Israel Institute of Technology, Haifa, Israel

## Abstract

**Background:**

The gastrointestinal tract is involved in nearly all patients with systemic sclerosis (SSc) and is a source of significant morbidity and even mortality.

**Aim:**

This study was aimed at assessing whether there is a correlation between upper gastrointestinal (UGI) endoscopy findings and mortality in SSc patients.

**Patients and Methods:**

The records of 256 SSc patients seen in our rheumathologic clinic between 2003 and 2013 were reviewed. Of these, 140 patients who had at least one detailed upper endoscopy report and at least 6 months follow-up were included in the study. Patient data included demographics, type of SSc, disease duration, modified Rodnan skin score (mRSS), lung, cardiac, renal or musculoskeletal involvement, hemoglobin at endoscopy and type of antibodies. Endoscopic findings that were included in the analysis were esophagitis, ulcerations, tumors, gastric antral vascular ectasia (GAVE), gastric erosions, submucosal hemorrhages and lumenal blood. The statistical methods used included descriptive statistics, T test, bivariable analysis, and cox regression analysis.

**Results:**

Forty-seven patients (16 diffuse SSc) had evidence of GAVE or antral erosions and hemorrhage. The mortality rate in this group during follow-up was 37% vs. 25% in the group of 93 (39 diffuse) SSc patients without GAVE or UGI bleeding (p=0.001). There were no statistical differences between the groups regarding mean age (55) or Hb (10.87 in the group with the UGI bleeding vs 11.77). The mean mRSS score was higher in the group with UGI bleeding 8 vs.5.6 (p=0.019). Mean (median) disease duration was 6.9 (4.5) years in the group with UGI bleeding vs 10.4 (10) (p< 0.001). Esophagitis was found in 90% of patients, despite use of PPI. Co-morbidity of myositis had a negative impact on survival. The mortality hazard ratio (95% CI) for UGI bleeding, myositis and interstitial lung disease were 5.9 (2.7–13.2), 4.9 (2–12.4) and 2.7 (1.3–5.8) respectively.

**Conclusions:**

A diagnosis of GAVE or UGI bleeding on upper endoscopy was associated with higher mortality in our cohort of SSc patients. Myositis was associated also with increased mortality. The long-term survival of patients with GAVE/UGI bleeding was similar to the patients with myositis that were free of such GI complications. The patients with both myositis and GI bleeding had a very poor prognosis.

# Sub article: Poster #013: The Implementation of Robotic Surgeries in Israel

**DOI:** 10.5041/RMMJ.10184

**Published:** 2015-01-29

**Authors:** Emad Matanes, Sari Bulous, Lior Lowenstein

**Affiliations:** Department of Obstetrics and Gynecology, Rambam Health Care Campus, Haifa, Israel; and The Ruth & Bruce Rappaport Faculty of Medicine, Technion-Israel Institute of Technology, Haifa, Israel

## Abstract

**Introduction:**

During the last decade the number of robotic devices and medical procedures utilizing them has increased significantly worldwide.

**Aims:**

This study aimed at evaluating the implementation of robotic surgeries in Israel in various surgical disciplines.

**Materials and Methods:**

A retrospective study was performed by accessing information about the annual purchases of surgical robots, the number of physicians trained for their use, and the number of robotic surgeries performed each year, according to indications of surgery and the disciplines of the operating medical staff. The data were taken from the database of Intuitive Surgical Inc. (Sunnyvale, CA, USA).

**Results:**

Six robots were purchased by six medical centers in Israel between 2008 and 2013. There are currently 150 doctors trained to use the robot in one of the simulators of Intuitive Surgical Inc. Of them, 104 doctors are listed as active robotic surgeons. Most are urologists, gynecologists, or general surgeons. The number of robotic surgeries increased each year in all fields in which it had been implemented. In 2013, 975 robotic surgeries were performed in Israel. Of them, 52% were performed by urologists; 80% of which were radical prostatectomies.

**Conclusion:**

The use of robotic surgery increased considerably in Israel over recent years, in urology, gynecology, general surgery, and otolaryngology. Despite the lack of conclusive evidence of the advantages of robotic surgery over the laparoscopic approach, the market power and the desire to be at the technological forefront is driving many medical centers to purchase surgical robots and to train physicians in their use.

# Sub article: Poster #057: Dimensional Changes Following Lateral Wall Sinus Augmentation without Concomitant Implant Placement Using a Composite Bone Graft

**DOI:** 10.5041/RMMJ.10184

**Published:** 2015-01-29

**Authors:** Eli E. Machtei, Doron Rozitky, Jacob Horwitz

**Affiliations:** 1 Department of Periodontology, School of Graduate Dentistry, Rambam Health Care Campus, Haifa, Israel;; 2 The Ruth & Bruce Rappaport Faculty of Medicine, Technion-Israel Institute of Technology, Haifa, Israel; 3 Division of Periodontology, Department of OMII, Harvard School of Dental Medicine, Boston, MA, USA.

## Abstract

**Introduction:**

Severely atrophic posterior maxilla usually requires lateral wall sinus augmentation to enable proper placement of dental implants. The kinetics of the grafting material resorption or contraction, following lateral wall sinus augmentation, is yet to be fully understood. The aim of the present study is to evaluate changes in sinus dimensions immediately post-op and 1 year later using a composite bone graft of Xenograft and autogenous bone (90:10 ratio) for lateral bone augmentation of the maxillary sinus.

**Materials and Methods:**

Patients that underwent lateral wall sinus augmentation procedure (without concomitant implant placement) were considered for this retrospective analysis if they had three consecutive panoramic radiographs: pre-treatment (T0), immediately after lateral wall sinus augmentation (T1) and approximately one year post-op (T2). Included in the study were 18 subjects, ages 47–69. Surgical protocol included bone grafting with a composite bovine derived Xenograft and autogenous bone graft (9/1 ratio). Digital panoramic radiographs were transferred into measurement software and the following measurements performed: (i) Vertical distance base of the alveolar ridge to the height of the augmented material; (ii) Sagittal area of the grafted area; and (iii) Vertical distance from the base of the ridge to the roof of the maxillary sinus. All measurements were performed by one examiner. Double measurements were performed in nine patients at T2 and the calculated Cronbach’s alpha statistics was high (0.9658–0.9882).

**Results:**

The mean overall sinus sagittal area was 1330±481 mm2 prior to the grafting procedure which decreased to 858.2±436 mm2 (380.7–2134 mm2, range) immediately post-op (p=0.0035) and stayed stable at one year (mean 882.2±384 mm2, p>0.05). Similarly, the overall area of the grafted material at the time of surgery was 468.3±208 mm2 and was only slightly reduced at 1 year (422.2±193 mm2). The mid-sagittal grafts height at T1 (mean 20.67±4.5 mm) was insignificant and minimal reduction at 1 year (mean 19.35 ± 4.9).

**Conclusions:**

The linear dimension of composite BDX/autogenous bone grafts following lateral wall sinus augmentation exhibited minimal changes during the 8–12 months healing period.

# Sub article: Poster #079: Effects of Pneumoperitoneum on Renal Function in Rats with Experimental Diabetes Mellitus as Compared with Normal Animals

**DOI:** 10.5041/RMMJ.10184

**Published:** 2015-01-29

**Authors:** Wisam Abboud, Niroz Abu-Saleh, Hoda Awad, Bishara Bishara, Zaid Abassi

**Affiliations:** 1 Division of Endocrine Surgery and Advanced Laparoscopy; 2 Research Unit, Rambam Health Care Campus and the; 2 Department of Physiology, Rappaport Faculty of Medicine, Technion, Haifa, Israel

## Abstract

**Introduction:**

Diabetes mellitus (DM) is a metabolic disease which may lead to diabetic nephropathy (DN). Pneumoperitoneum is known to induce adverse influence on the normal functions of the heart, lung and kidneys. Clinical and experimental studies in healthy subjects or animal models have shown a decrease in urine flow in laparoscopic surgery as compared with open approach. This study was designed to examine the effects of incremental increases in intra-abdominal pressure (IAP) from 0 to 14 mmHg on and renal function and acute kidney injury (AKI) in rats with DM as compared with healthy animals.

**Materials and Methods:**

Following IAP of 0 (baseline), rats with DM induced by streptozocin (60 mg/kg, intra-abdominally) one week prior to operation or healthy controls were subject to IAP of 7, 10 or 14 mmHg, over 45 min for each pressure, followed by deflation period of 60 min (recovery). Urine flow rate (V), Na+ excretion (UNaV), glomerular filtration rate (GFR), and renal plasma flow (RPF), were determined throughout the experiment. Urinary levels of NGAL, a novel biomarker of AKI, were determined in both groups.

**Results:**

There were no significant changes in V, UNaV, GFR, and RPF during 7 mmHg insufflation. Reductions in these parameters were observed in control rats during 10 and 14 mmHg. Baseline values of renal hemodynamics were significantly lower in diabetic rats. Diabetic rats subjected to 10 and 14 mmHg exhibited aggravated declines in V, UNaV, GFR, and RPF. Elevation of IAP induced a pressure dependent increase in urinary NGAL in control rats. Similarly, pneumoperitoneum provoked an increase in urinary NGAL in diabetic animals; however, this increase was more profound than that obtained in non-diabetic rats.

**Conclusions:**

Adverse renal function/perfusion of IAP of 10 and 14 mmHg were observed in healthy controls and to a larger extent in diabetic animals. Moreover, diabetic rats are more susceptible to the adverse real effects of elevated IAP as was evident by enhanced urinary excretion of NGAL.

# Sub article: Poster #009: Improved Six-month Outcome of Helicopter-evacuated Patients with Severe Head Injuries: 2008–2013

**DOI:** 10.5041/RMMJ.10184

**Published:** 2015-01-29

**Authors:** Leon Levi, Hanny Bahouth, Menashe Zaaroor

**Affiliations:** 1 Department of Neurosurgery, Rambam Health Care Campus, Haifa, Israel; 2 Trauma Unit, Rambam Health Care Campus, Haifa, Israel

## Abstract

**Introduction:**

The trauma service at Rambam Health Care Campus is the busiest in Israel, and handles a particularly heavy load of patients with severe brain injuries (SHI). Since 2007, Magen David Adom (MDA) has used helicopters to supplement transport of casualties to Rambam, thereby reducing the proportion of hospital land referrals to Rambam from 62% (prior to 2008) to 40%.

**Methods:**

From 2008–2013, 1,112 patients were admitted with SHI (comatose and typical CT findings). We recorded pertinent information and verified mortality data at 6-month of injury with correlation to core+CT-IMPACT death probability (based on international data). Three modes of evacuation from the site of injury were assessed: paramedic staffed ambulances (Direct, DTR), helicopter (HEMS), and via referral from another hospital (TRANS). Hospital care was classified as neurosurgical mass lesion evacuation (EM), other neurosurgical surgery (Not-EM), and no neurosurgery performed. The differences between individual IMPACT mortality risk and actual deaths in each subgroup were assessed for significance.

**Results:**

The following distributions were noted: 71% of all SHI patients underwent neurosurgery. Before 2008, only 6% were evacuated by HEMS as compared to 29% today. The mortality of the three groups of patients was significantly different: 31.4% mortality for the EM group as opposed 13.8% for the Not-EM group, and 17% for patients who did not undergo neurosurgery (one half of whom were designated “Do Not Resuscitate”). The modern SHI-specific mortality predictor was a good benchmark. The most significant reduction in mortality was achieved by HEMS evacuation of patients needing neurosurgery—a 34.8% improvement over the predicted mortality for mass lesions, and a 46.2% improvement for patients needing neurosurgical procedures. This represented a marked improvement as compared to other groups. Other differences such as the evacuation distance, speed of transfer, and resuscitative measures were also examined; these data pointed to a marked benefit of HEMS for casualties in distant locations.

**Conclusions:**

Patient transfer by HEMS facilitated advanced care, as determined by the mortality risk factors, resulting in increased survival—without any other marked improvement in medical treatment. The greatest advantage was noted for patients evacuated by helicopter when comparing risk and actual mortality, particularly in comatose patients in urgent need of neurosurgery. Regional improvement is based on local data that can readily support decisions for continuous improvement, and to achieve results higher than the international benchmark.

# Sub article: Poster #008: Novel Peptides Inhibiting Heparanase Procoagulant Activity in a Mouse Tumor Model

**DOI:** 10.5041/RMMJ.10184

**Published:** 2015-01-29

**Authors:** Yonatan Crispel, Elena Axelman, Mifleh Tatour, Inna Kogan, Israel Vlodavsky, Yona Nadir

**Affiliations:** 1 Thrombosis and Hemostasis Unit, Rambam Health Care Campus, The Bruce Rappaport Faculty of Medicine, Technion Haifa, Israel; 2 Cancer and Vascular Biology Research Center, The Bruce Rappaport Faculty of Medicine, Technion, Haifa, Israel

## Abstract

**Background:**

Heparanase is implicated in angiogenesis and tumor progression. We have demonstrated previously that heparanase may also affect the hemostatic system in a nonenzymatic manner. It forms a complex and enhances the activity of the blood coagulation initiator tissue factor (TF). Peptides inhibiting heparanase procoagulant activity, generated by us, were recently demonstrated to be effective in heparanase injected and sepsis mice models. In the present work we studied the peptides effect in a tumor model.

**Methods:**

B16 mouse melanoma, EMT-6 mouse breast cancer, and MDA-231 human breast cancer were injected subcutaneously (s.c.). Inhibitory peptides 5, 6 and 7 were injected s.c. at a dose of 1–3 mg/kg, every other day, starting 4 days after tumor cell injection. The experiments ended after 3 weeks. In order to evaluate the effect on tumor relapse, the tumor was left to grow to 1–1.5 cm, and then extracted following peptides injections for 3 weeks. Tumor, organs, and plasma were studied.

**Results:**

Peptides 5, 6 and 7 inhibited tumor size and vascularization in a dose-dependent manner reaching a 2/3 reduction, compared to control tumors, using the three tumor cell lines. Additionally, survival advantage (P<0.05) and reduced plasma thrombin-antithrombin complex (P<0.05) were observed in the treatment groups. Peptides completely inhibited tumor relapse after extraction of the primary tumor. *In vitro*, peptides did not inhibit tumor cell growth and had no effect on heparanase enzymatic activity.

**Conclusions:**

Peptides inhibiting heparanase procoagulant activity significantly inhibited tumor growth, vascularization, and relapse. The effect of peptides in cancer patients should be further investigated.

# Sub article: Poster #062: Can a Categorical Sub-division of the Standard IPSS Questionnaire be Used as a Tool for Assessing Urinary Outlet Obstruction?

**DOI:** 10.5041/RMMJ.10184

**Published:** 2015-01-29

**Authors:** Ravit Yehiely-Cohen, Omar Massarwi, Dallal Hatib, Ilan Gruenwald

**Affiliations:** 1 Neurourology Unit, Rambam Health Care Campus, Haifa, Israel; 2 The Ruth & Bruce Rappaport Faculty of Medicine, Technion-Israel Institute of Technology, Haifa, Israel

## Abstract

**Introduction:**

Urodynamic studies serve as an objective method for evaluating lower urinary tract function. Urodynamic parameters are the basis for characterization of urinary disorders of both the filling and/or voiding phase. One limitation of urodynamic testing is that the unnatural environment for urination may negatively affect the text results and not accurately represent actual lower urinary tract function. We examined the feasibility of using the International Prostate Symptom Score (IPSS), specifically its sub-categorical questions, as a reliable diagnostic tool for evaluation of voiding disorders caused by bladder outlet obstruction.

**Materials and Methods:**

Eighty-nine adult patients who underwent urodynamic testing completed the IPSS questionnaire, which included four questions regarding bladder emptying (voiding phase) and three questions regarding bladder filling (filling phase). We compared the objective parameters measured during the urodynamic pressure/flow study (the standard measurement for assessing the degree of outflow obstruction) with the degree of voiding difficulty as scored by the IPSS sub-category questions. In an attempt to find a score that accurately reflected a state of obstruction, we correlated the bladder voiding and filling phase questions to the pressure flow measurements.

**Results:**

Out of 89 patients (mean age 49, 30 men, 59 women), 43 (48.3 %) had a higher score in the voiding phase vs the filling phase (ratio score > 1), suggesting that this cohort of patients had more emptying difficulties than storage ones. In contrast, according to the objective urodynamic pressure/flow study only 10 out of 89 (11.2 %) subjects fell under the category of obstructive voiding disorder. The mean total score for the emptying disorder questions in this sub-group was 12.9 (range 5–20), which was of high statistical significance for obstruction (p=0.017). Seventy-nine (88.7 %) of the subjects were not obstructed according to the pressure/flow study; their mean total scores on questions dealing with emptying phase was 7.8 (range 0–20). Statistical analysis showed that a score of 9.27 or higher for the voiding phase, as expressed in the pressure flow study, indicated an obstructive disorder (p=0.05), while a score of 8 and below characterized patients with voiding difficulties but without an obstruction (p<0.05).

**Conclusions:**

Based on our findings, a mean score above 12.9 for the emptying phase questions clearly indicates outflow obstructive disorder as seen in the pressure/flow study, while a score below 8 is typical of patients with no obstructive voiding. Our study indicates that this simple 7-question questionnaire could be easily used as a clinical tool for assessment of urinary outlet obstruction.

# Sub article: Poster #063: Shockwave Treatment for Erectile Dysfunction: Long-Term Efficacy at Two Years

**DOI:** 10.5041/RMMJ.10184

**Published:** 2015-01-29

**Authors:** Ilan Gruenwald, Omar Massarwa, Boaz Appel, Noam Kitrrey, Yoram Vardi

**Affiliations:** 1 Neurourology Unit, Rambam Health Care Campus, Haifa, Israel;; 2 The Ruth & Bruce Rappaport Faculty of Medicine, Technion-Israel Institute of Technology, Haifa, Israel; 3 Sheba Medical Center, Ramat Gan, Israel

## Abstract

**Introduction:**

The beneficial short-term effect of low-intensity shockwave treatment for erectile dysfunction has been previously studied, but its long-term efficacy has not been fully evaluated. The objective of this study is to examine if patients that responded well to shockwave treatment maintain their response after two years.

**Patients and Methods:**

Patients who had responded satisfactorily to shockwave treatment in our department and had a minimal period of two years follow-up were included in the study. For inclusion, their positive initial response was defined according to the improvement in the IIEFEF domain questionnaire (Rosen minimal change clinical improvement), showing a significant improvement in their erectile function at three months post-treatment (second follow-up visit). These men were prospectively observed after 6, 12, 18, and 24 months and their erectile function was evaluated by an interview (face-to-face, telephone, or an e-mail) that included IIEF-EF domain questionnaires and the CGIC (Clinical Global Impression of Change).

**Results:**

Out of 192 treated patients who had surpassed the two-year follow-up, 113 (58.8%) met the inclusion criteria. Their median age was 60 years (27–78); 37 patients (33%) had diabetes mellitus and 36 patients (32%) had cardiovascular disease. Forty-seven patients (42%) had severe ED (IIEF-ED score between 1–10) before starting shockwave therapy. In the entire group, 85%, 66%, 62%, and 52% of the patients maintained their initial success after 6, 12, 18, and 24 months, respectively. The success rate after two years was not significantly different between diabetic and cardiovascular patients, but patients with mild-moderate ED succeeded more than those with severe ED (29% vs. 62%; P<0.01).

**Conclusions:**

The positive effect of shockwave treatment for erectile dysfunction declines gradually over two years in about half of the patients. This downtrend is more profound in severe ED patients. Further research is needed to develop modifications to the treatment protocol that will make the effect of shockwave therapy last longer.

# Sub article: Poster #064: Sexual Dysfunction in Patients with Chronic Pain

**DOI:** 10.5041/RMMJ.10184

**Published:** 2015-01-29

**Authors:** Ilan Gruenwald, Tziki Adler, May Haddad, Elon Eisenberg

**Affiliations:** 1 Neurourology Unit, Rambam Health Care Campus, Haifa, Israel;; 2 The Ruth & Bruce Rappaport Faculty of Medicine, Technion-Israel Institute of Technology, Haifa, Israel; 3 Pain Research Unit, Pain Medicine Institute, Rambam Health Care Campus, Haifa, Israel

## Abstract

**Introduction:**

Chronic pain is associated with disability in various daily activities, which is well documented. In contrast, there is paucity of reports regarding the prevalence and characteristics of sexual dysfunction (SD) in these patients.

The aim of the present study was to evaluate the prevalence and characteristics of SD in a cohort of patients suffering from chronic pain.

**Patients and Methods:**

The study population consisted of patients with chronic pain who agreed to participate in a study on SD. They completed questionnaires assessing demographics, pain-intensity (Visual Analogue Scale=VAS), pain qualities (Short-form McGill Pain questionnaire=SFMPQ), disability (Ostwestry Disability Index= ODI), depression (Beck depression inventory= BDI) and SD, including the Female Sexual Function Index (FSFI) and the International Index of Erectile Function (IIEF) for men. Chi Square and T-Test were used to compare between different sub-groups within the cohort. Spearman’s coefficient test was used to test correlations between pain and SD parameters.

**Results:**

Four-hundred and four out of 709 responding patients (58%) reported pain related SD, 249 (61.6%) were men and 155 (38.4%) women. No differences in age, weight, years of marriage, gender, and education were found between those with and without SD. In contrast, patients with SD exhibited significantly higher scores in the following pain and disability questionnaires [mean±STD, (median)]; VAS: 70±23 (72) vs. 50±27 (47), p<0.001; SF-MPQ: 16.5±10 (15) vs. 12.3±8 (10), p<0.001; ODI: 23.5±8.6 (24) vs. 16.4±8.5 (15), p<0.001. Patients with SD consumed significantly higher doses of pain medications than those without SD. Sexual dysfunction in women (FSFI) was 50.7 (out of 90 points) and in men (IIEF)-15, indicating moderate SD in both genders. On both numerical (0–100) and categorical (0–4=no to extreme SD) self-report scales of SD severity, women scored significantly higher than men (73.8±24.2 vs. 67.8±22.9; p=0.024 and 3.1±0.9 vs. 2.8±0.9; p=0.016 respectively). A significant correlation was found between pain intensity and severity of SD (r=0.349, p<0.001).

**Conclusions:**

A significant number of patients with chronic pain suffer from SD. Pain intensity correlated to SD severity. Although SD in this population is more prevalent in men, its self-reported severity is higher in women. These findings highlight the importance of increased awareness to SD in patients with chronic pain.

# Sub article: Poster #065: Is Genital Sensation Affected by Pelvic Organ Prolapse?

**DOI:** 10.5041/RMMJ.10184

**Published:** 2015-01-29

**Authors:** Ilan Gruenwald, Irena Gertman, Lior Lowenstein

**Affiliations:** 1 Neurourology Unit, Rambam Health Care Campus, Haifa, Israel;; 2 The Ruth & Bruce Rappaport Faculty of Medicine, Technion-Israel Institute of Technology, Haifa, Israel; 2 Department of Gynecology, Rambam Health Care Campus, Haifa, Israel

## Abstract

**Introduction:**

Reports in the literature show that pelvic organ prolapse (POP) is one of the confounding factors for female sexual function, probably due to anatomical and functional aspects. In this study we aimed to identify if there is any sensory factor involved that may cause female sexual dysfunction by comparing vaginal and clitoral sensory thresholds in women with POP to women with normal pelvic anatomy.

**Materials and Methods:**

Quantitative sensory thresholds for warm, cold, and vibratory sensetions were measured at the vagina and clitoral area of women with pelvic organ prolapse (POPQ stage II or higher, study group) and women without prolapse (POPQ stage I, control group). The quantitative sensory tests were performed with a thermal and vibration Genito-Sensory Analyzer (GSA; Medoc Ltd., Israel). Testing was done at the distal third of the anterior and posterior vaginal wall and at the clitoral area. Warm stimuli were used to evaluate C-fibers; cold stimuli for A-beta, A-delta, and C fibers; and vibratory stimuli for A-delta fibers. Independent student and Chi square of association were used for analysis of continuous and categorical parameters, respectively.

**Results:**

Overall, 67 women; 22 with median POP-Q stage III (range: II–IV) and 45 with POP-Q stage I, participated in the study. There were no statistically significant differences between the two groups in the characteristics examined (health status, medical history, and age), other than hypertension and parity; the former was more common in women with prolapse than in the control group (43% vs 13%), and the latter was higher (median: 3 vs. 2, P<0.001). In all regions examined, mean thresholds for vibratory and warm stimuli were significantly higher and mean thresholds for cold stimuli significantly lower in the group with prolapse.

**Conclusions:**

Women with pelvic organ prolapse exhibited lower sensitivity in the genital area to vibratory and thermal stimuli than did women without prolapse. Our findings suggest that a neuropathic sensory deficit in the area of the genitalia may be associated with prolapse. Investigation of possible correlations between the differences in stimulatory thresholds and clinical symptoms is needed, as well as the effect of these findings on sexual function and satisfaction.
